# Rational peptide design for regulating liquid–liquid phase separation on the basis of residue–residue contact energy

**DOI:** 10.1038/s41598-022-17829-1

**Published:** 2022-08-12

**Authors:** Kiyoto Kamagata, Maulana Ariefai, Hiroto Takahashi, Atsumi Hando, Dwiky Rendra Graha Subekti, Keisuke Ikeda, Atsushi Hirano, Tomoshi Kameda

**Affiliations:** 1 Institute of Multidisciplinary Research for Advanced Materials, Tohoku University, Katahira 2-1-1, Aoba-ku, Sendai, 980-8577 Japan; 2 Department of Chemistry, Faculty of Science, Tohoku University, Sendai, 980-8578 Japan; 3 Graduate School of Life Sciences, Tohoku University, Sendai, 980-8577 Japan; 4 Department of Biointerface Chemistry, Faculty of Pharmaceutical Sciences, University of Toyama, 2630 Sugitani, Toyama, 930-0194 Japan; 5 Nanomaterials Research Institute, National Institute of Advanced Industrial Science and Technology (AIST), Tsukuba, Ibaraki 305-8565 Japan; 6 Artificial Intelligence Research Center, National Institute of Advanced Industrial Science and Technology (AIST), Koto, Tokyo 135-0064 Japan

**Keywords:** Biophysics, Intrinsically disordered proteins

## Abstract

Since liquid–liquid phase separation (LLPS) of proteins is governed by their intrinsically disordered regions (IDRs), it can be controlled by LLPS-regulators that bind to the IDRs. The artificial design of LLPS-regulators based on this mechanism can be leveraged in biological and therapeutic applications. However, the fabrication of artificial LLPS-regulators remains challenging. Peptides are promising candidates for artificial LLPS-regulators because of their ability to potentially bind to IDRs complementarily. In this study, we provide a rational peptide design methodology for targeting IDRs based on residue–residue contact energy obtained using molecular dynamics (MD) simulations. This methodology provides rational peptide sequences that function as LLPS regulators. The peptides designed with the MD-based contact energy showed dissociation constants of 35–280 nM for the N-terminal IDR of the tumor suppressor p53, which are significantly lower than the dissociation constants of peptides designed with the conventional 3D structure-based energy, demonstrating the validity of the present peptide design methodology. Importantly, all of the designed peptides enhanced p53 droplet formation. The droplet-forming peptides were converted to droplet-deforming peptides by fusing maltose-binding protein (a soluble tag) to the designed peptides. Thus, the present peptide design methodology for targeting IDRs is useful for regulating droplet formation.

## Introduction

Membraneless organelles, such as stress granules and nucleoli, are liquid droplets formed by liquid–liquid phase separation (LLPS)^1–5^. LLPS-related proteins, such as FUS^6^, LAF-1^7^, and p53^8^, form liquid condensates, which generate or regulate various biological functions at levels that cannot be achieved by the dilute bulk phase alone. Liquid-like droplets are stabilized by multivalent intermolecular interactions between amino acid residue pairs of intrinsically disordered regions (IDRs) of proteins, such as cation–π, hydrophobic, π–π, and electrostatic interactions^6,8–13^. In addition, the molecular uptake into the droplets is governed by cation–π and electrostatic interactions^6,14^. Despite extensive knowledge, the establishment of an artificial LLPS-regulator design that targets IDRs remains a challenge. Artificial LLPS-regulators could be used for various applications, including the biological investigation of LLPS function, the high-efficiency production of chemicals via droplet-based enzymatic systems, and the development of aggregation suppressors as drug candidates in aggregation-associated diseases.

Peptides are LLPS-regulator candidates. Peptides potentially have an affinity for the IDRs of target proteins, which originates from their ability to enable their flexible fitting to any conformation of the IDRs on the basis of a combination of 20 amino acid residues with different characteristics. We previously reported on a designed peptide that targets a disordered region of p53 and regulates its function^15^. It has also been shown that artificial peptides can promote liquid droplets of FUS^16^. As such, peptide design is a promising approach for the targeting of IDRs and regulating of LLPS behavior. However, the theoretical sequence space of peptides is extremely large; for example, 20^16^ candidates are possible for a 16-residue peptide. Hence, an experimental screening of peptide candidates will be impossible. Therefore, there is a need to establish a computer-based method for peptide design.

In a previous study, our peptide design resulted in a peptide sequence that binds to the IDR of p53^15^, which was based on 3D structure-based contact energy of residue pairs, as proposed by Miyazawa and Jernigan^17,18^. 3D structure-based energy has also been used in the simulations of protein folding^19–23^ and peptide aggregation^24^ as well as in the prediction of protein structure^25,26^. The 3D structure-based contact energy was calculated based on the contact frequency of residue–residue pairs in the available protein 3D structures^17,18^. Accordingly, the 3D structure-based contact energy reflects the structural restrictions originating from the folded proteins. In contrast to the folded proteins, IDRs are structurally flexible; therefore, 3D structure-based contact energy may not necessarily be appropriate for the design of a peptide targeting IDRs. A newly defined contact energy is thus needed to successfully describe the interactions between IDRs and IDR-targeting peptides.

In this study, we present a rational design methodology to obtain a peptide that can bind to an IDR using only the IDR sequence information. This methodology is based on the contact energies of residue pairs obtained using molecular dynamics (MD) simulations. Using this methodology, we designed peptides targeting the N-terminal IDR of p53, which participates in liquid droplet formation^8^. We describe how the newly defined contact energy improves design quality. Furthermore, we examined the action of the designed peptides on the p53 droplets.

## Results

### MD simulations provide the contact energy for amino acid residue pairs without backbone steric constraints

**Figure 1:**
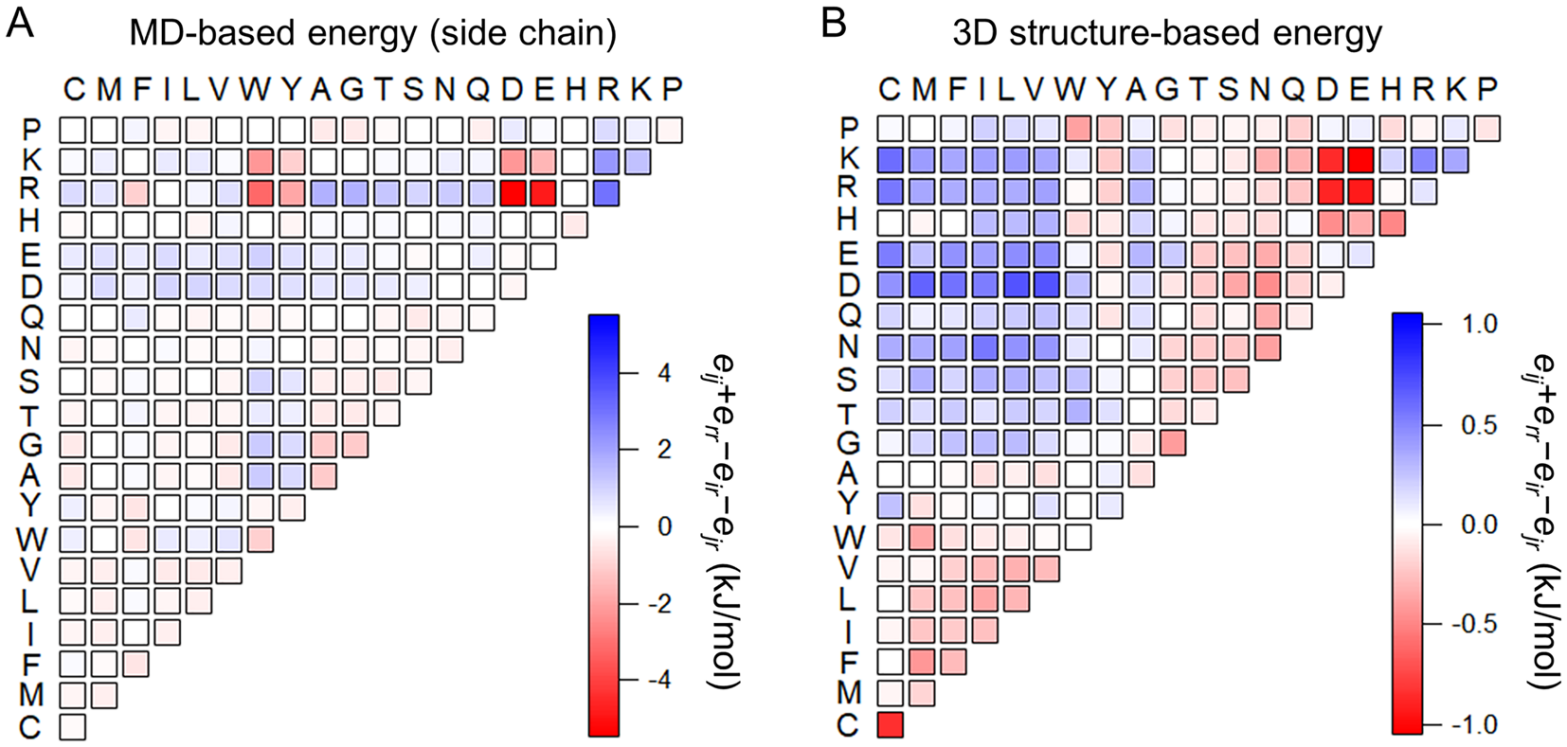
Comparison between MD-based energy and 3D structure-based energy for residue pairs. (**A**) MD-based relative contact energy matrix for side chains. (**B**) 3D structure-based relative contact energy matrix obtained by Miyazawa and Jernigan^18^.

To establish a general methodology for designing peptide sequences targeting disordered proteins, we calculated the contact energy (binding free energy) between residue pairs, namely two amino acids without backbone groups (amino and carboxyl groups), using MD simulations. This energy is referred to as MD-based energy (side chain) here. The MD-based energy (side chain) for a pair of residues *i* and *j*, *e*_*ij*_, was calculated using a previously described method^27^ (see Supplementary Table S1 and “Materials and methods”). Figure 1A shows the relative contact energy matrix for the MD-based energy (side chain), *e*_*ij*_ + *e*_*rr*_ − *e*_*ir*_ − *e*_*jr*_, where *r* represents the average amino acid residue, as defined by Miyazawa and Jernigan^18^ (see “Materials and methods”). Positively charged residues (R and K) interact strongly with negatively charged residues (D and E) via attractive electrostatic interactions and with aromatic residues (Y, W, and F) through cation–π or hydrophobic interactions. Note that the relative contact energy matrix for the MD-based energy of whole amino acids having backbone groups (amino and carboxyl groups) showed a similar pattern to that of the MD-based energy (side chain) with a strong correlation (*r* = 0.76; Supplementary Fig. S1A), which demonstrates that backbone groups marginally affect residue–residue interactions. Thus, in this study, the MD-based energy (side chain) was adopted to establish a general methodology for designing peptide sequences targeting disordered proteins (hereafter referred to as MD-based energy).

For comparison with the MD-based energy, we also calculated the relative contact energy of the 3D structure-based energy (Fig. 1B)^18^. The 3D structure-based energy is weakly correlated with the MD-based energy (*r* = 0.48), which is reasonable given that the 3D structure-based energy calculation is based on the folded protein structures. Specifically, the 3D structure-based energy provides the residue–residue contact energy reflecting the backbone steric constraint in the folded proteins, in contrast to the MD-based energy. In this comparison, both energies confirm significantly favorable (attractive) interactions between positively charged residues (R and K) and negatively charged residues (D and E) (Fig. 1A,B). The MD-based energy indicates that the R residue has favorable interactions with aromatic residues (F, W, and Y), which is attributable to cation–π interaction (Fig. 1A,B). Based on this result, it is reasonable that the cation–π interaction is more dominant for disordered proteins than for folded proteins; in fact, cation–π interaction was reported to contribute to the LLPS of disordered proteins^6,28,29^. In addition, the 3D structure-based energy indicates moderately favorable interactions between hydrophobic residues (M, I, L, V, and F), which play a role in stabilization of the folded structures by forming hydrophobic packing in the interior. Furthermore, the 3D structure-based energy indicates a unique attractive interaction between C residues, which reflects the disulfide bond formation often observed in folded structures. Overall, the MD-based energy would more accurately predict residue–residue interactions of proteins and peptides with high degrees of freedom (flexibility), such as IDRs, than 3D structure-based energy.

### Reliability of force fields

To ensure the reliability of the MD simulation, we compared the binding free energy data between different force fields. Binding free energy data were obtained using the AMBER99SB force field. The data were significantly correlated with those obtained in other force fields: CHARMM22^30,31^ (*r* = 0.87; Supplementary Fig. S1B,C) and OPLS-AA/L^32^ (*r* = 0.82; Supplementary Fig. S1D,E). This indicates that our MD simulation results were independent of the force field and general.

### MD-based contact energy provides higher affinity peptides for the p53 N-terminal IDR than 3D structure-based energy

As mentioned above, the MD-based energy should describe more precisely the binding affinity between IDRs with less backbone steric constraints than the 3D structure-based energy. Here, we examined whether a peptide design with MD-based energy improves the affinity of peptides for IDRs compared to that with the 3D structure-based energy used in a previous report^15^. A practical approach involves regulating the phase separation of LLPS-related proteins by the designed peptides; in other words, the droplet-forming IDR of the proteins is a promising target for demonstrating the advantages of the present peptide design. Specifically, a peptide could be designed to target and block the droplet-forming IDR, inhibiting the association between the LLPS-related protein molecules and thus suppressing droplet formation. In another scenario, a designed peptide may bridge multiple LLPS-related protein molecules, promoting droplet formation, as reported previously^16^. In any case, designed peptide can regulate the LLPS of the target proteins.

**Figure 2:**
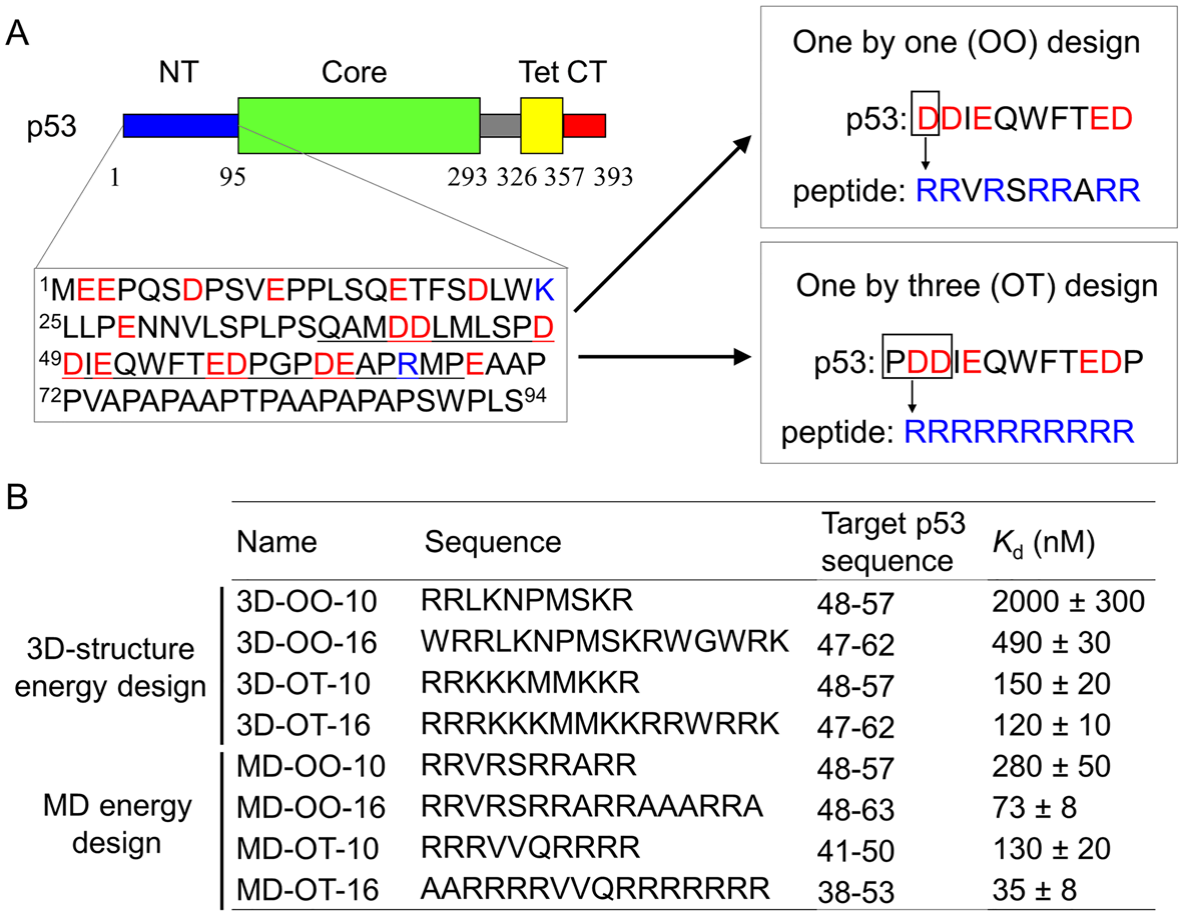
Peptide design scheme and binding properties of the designed peptides to p53 N-terminal IDR. (**A**) Schematic diagram of peptide design targeting p53 N-terminal IDR. NT, Core, Tet, and CT in the primary sequence of p53 represent the N-terminal, core, tetramerization, and C-terminal domains, respectively (top left), where thick and thin bands represent folded and disordered regions, respectively. The NT sequence (1–94) was used for the present peptide design (bottom right). Blue and red characters denote positive and negative charged residues, respectively. The peptide sequences with the highest binding property were designed based on the calculation of one-by-one (OO) or one-by-three (OT) contact energy for each residue of the p53 sequence (right). Ten-residue peptides designed with the MD-based energy are shown as examples. (**B**) Sequence, complimentary target residue number, and dissociation constant (*K*_d_) of designed peptides used in this study. The top and bottom four peptides were designed with the 3D structure-based and MD-based energies, respectively. For example, 3D-OO-10 and MD-OT-16 designate a 10-residue peptide designed with 3D structure-based OO contact energy and a 16-residue peptide designed with the MD-based OT contact energy, respectively. *K*_d_ was determined by titrating peptides against the target p53 N-terminal peptide fragment underlined in (**A**). Errors represent the fitting errors of titration data.

In this study, we chose the N-terminal IDR of p53 as a model target because it participates in droplet formation together with the C-terminal IDR^15^ (Fig. 2A). Using the MD-based and 3D structure-based energies, we designed eight peptide sequences to bind to the p53 N-terminal IDR according to a previously described method^15^ (Fig. 2A,B). Briefly, the peptides were designed to minimize their total contact energy (binding free energy) for one-by-one (OO) residue pairs or one-by-three (OT) residue pairs, including two adjacent target residues (Fig. 2A), over the p53 N-terminal IDR (Supplementary Fig. S2). Our previous study demonstrates that the peptide binder with OT design had higher affinity for the p53 C-terminal IDR than that with OO design^15^. We designed 10- and 16-residue peptides, since peptides with these lengths were reported to regulate p53 function^15^ and FUS droplets^16^. It should be noted, in this methodology, that when candidate peptide sequences were all or almost all composed of the same residues (for example, the peptide designed using MD-based contact energy for the OT residue pairs was found to consist of only R), we adopted the sequence with a binding energy close to the minimum. The reason for this was that such single amino-acid repeats potentially decrease the interaction specificity of the peptides for IDR regions of target proteins due to the increased chance of unexpected interactions with other regions of them (Supplementary Fig. S2). Nevertheless, the designed peptides that were adopted contained many positively charged residues (R and K), complementary to the negatively charged residues of p53 N-terminal IDR (Fig. 2B). The design with the MD-based energy tends to have an R residue rather than a K residue compared to the design with 3D structure-based energy. In addition, the designed peptides contained neutral polar residues (L, N, S, and Q) and non-polar hydrophobic residues (A, P, W, and V), complementary to the non-charged residues of p53 N-terminal IDR (Fig. 2B).

To quantify the target binding ability of the designed peptides, we titrated the designed peptides against the p53 N-terminal peptide fragment (residues 37–65; underline in Fig. 2A) labeled with a fluorescent dye, 5-carboxyfluorescein (FAM), using a fluorometer with fluorescence anisotropy^33^. All titration curves were well fitted with Eqs. (6) and (7) (see “Materials and methods”) based on one-by-one binding (Supplementary Fig. S3). The dissociation constants of the designed peptides (*K*_D_) ranged from 35 ± 8 nM to 2.0 ± 0.2 µM (Fig. 2B). Designed peptides with MD-based energy had 1.2- to 7.1-fold stronger affinity than those with 3D structure-based energy under the same conditions of peptide design (OO or OT) and peptide length. Accordingly, these results indicate that the design methodology with MD-based energy can fabricate stronger peptide binders to the p53 N-terminal peptide than that with 3D structure-based energy.

To confirm the specific targeting of designed peptides, we tested whether the peptides designed to bind to N-terminal IDR of p53 interact to other region of p53 (e.g. C-terminal IDR). The titration measurements demonstrate that all designed peptides did not bind to the C-terminal IDR in similar concentration ranges (Supplementary Fig. S4).

### All designed peptides targeting the N-terminal IDR promote the formation of p53 droplets

**Figure 3:**
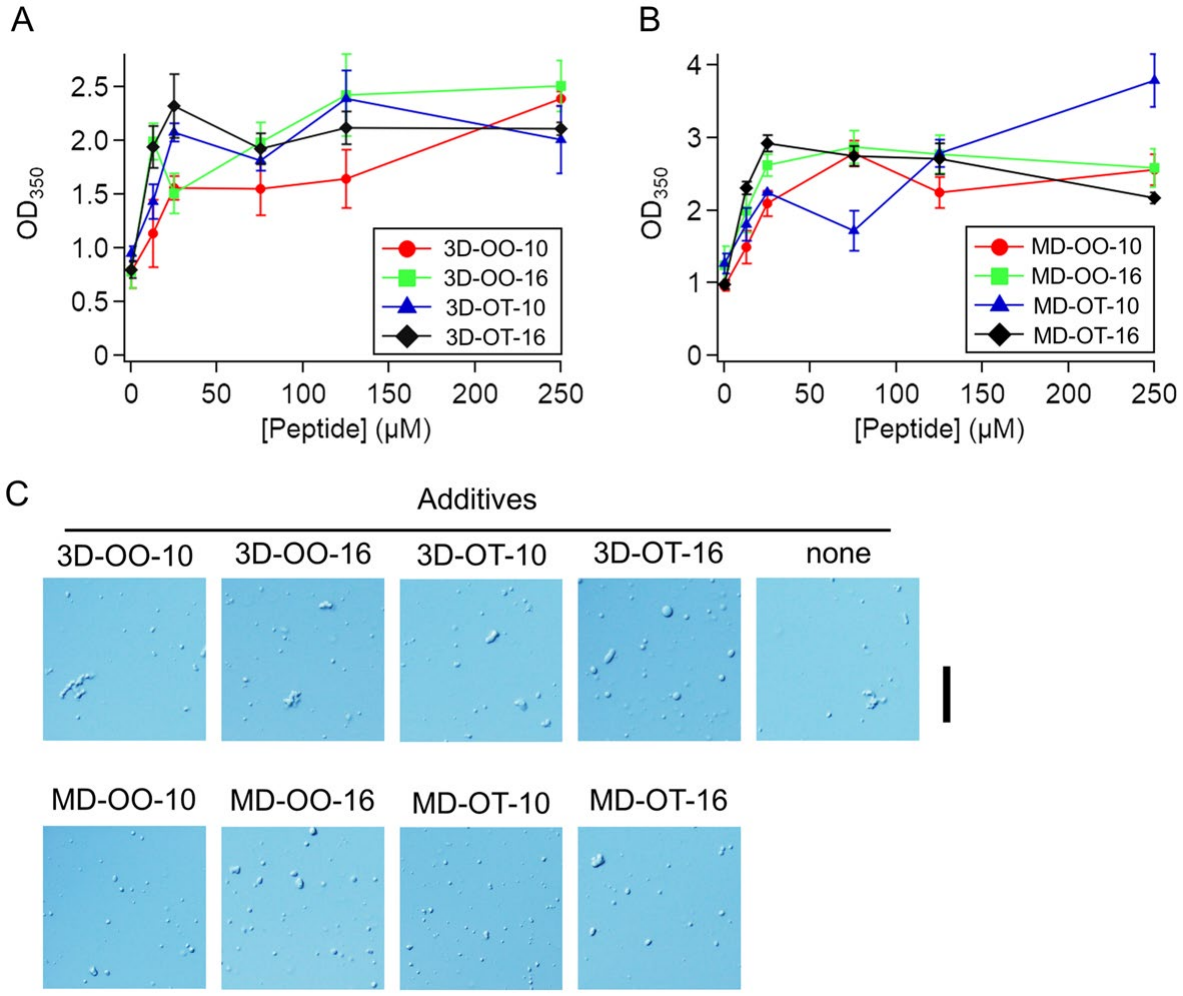
All designed peptides targeting p53 N-terminal IDR promote p53 droplet formation. (**A**) Effect of the 3D structure-based designed peptides targeting the N-terminal IDR as additives on p53 droplet formation detected as OD_350_. (**B**) Effect of the MD-based designed peptides targeting the N-terminal IDR as additives on p53 droplet formation detected as OD_350_. (**C**) DIC images of p53 solutions in the presence and absence of 250 µM designed peptides. “None” denotes the absence of designed peptides as control. Scale bar, 20 µm. In (**A** and **B**), error bars represent standard error (*N* ≥ 3).

We investigated the effects of the designed peptides on p53 droplet formation. p53 forms liquid droplets in 45 mM NaCl at pH 7.0, whereas p53 disperses over solution in 450 mM NaCl at pH 7.5, as previously described^8^. In this study, p53 droplet formation was initiated by the NaCl concentration jump from 450 to 45 mM, as well as a pH jump from 7.5 to 7.0, along with a 10-fold reduction in the p53 concentration to 12.5 µM. Analysis was performed after 5 min of incubation. An increase in light scattering at 350 nm was observed for every sample containing designed peptides at concentrations higher than 25 µM (which corresponds to a 2-fold concentration of p53) (Fig. 3A,B). Imaging of the solutions using a differential interference contrast (DIC) microscope confirmed the formation of micrometer-sized droplets in the presence of 250 µM designed peptides (Fig. 3C). For qualitative analysis, we examined the effect of the designed peptides on the size and shape of the droplets using DIC microscopy (Supplementary Fig. S5A–D). The addition of the designed peptides did not significantly affect the relative distribution of the cross-sectional area and circularity of the droplets. No significant decrease of circularity implies that the designed peptides do not convert from liquid droplets to solid aggregates. In contrast, the number of droplets was increased 1.3–2.3-fold in the presence of 6 designed peptides (Supplementary Fig. S5E). Furthermore, the designed peptides induced p53 droplets below the critical concentration of p53, supporting the promoted droplet formation (Supplementary Fig. S6). The recruitment of designed peptides into droplets supported the interaction between the designed peptides and p53 inside droplets (Supplementary Fig. S7). Taken together, the designed peptides with different affinity for p53 showed similar effects of increasing the number of p53 droplets likely by bridging multiple p53 molecules in droplets and/or by reducing the electrostatic repulsion between p53 molecules.

### The soluble tag converts the designed peptide from an accelerator to a suppressor of the p53 droplet formation

Based on these results, we were motivated to establish a strategy that converts the designed peptides from accelerators to suppressors for droplet formation. Two approaches were proposed: (1) a soluble tag fusion approach, and (2) a reduced interaction approach. The former approach aims to increase the solubility of the designed peptide–target protein complex by attaching a soluble tag to the peptides while maintaining the target-binding ability of the peptides. The latter aims to weaken the affinity of the designed peptides for the target (p53 N-terminal peptide) by reasonably modifying the peptide sequences; specifically, the R residue of the peptides is replaced with a K residue (these peptides are referred to as R-to-K peptides), because the R residue interacts more strongly with the corresponding p53 N-terminal IDR residues, including W and F, than the K residue.

#### Soluble-tag fusion approach

**Figure 4:**
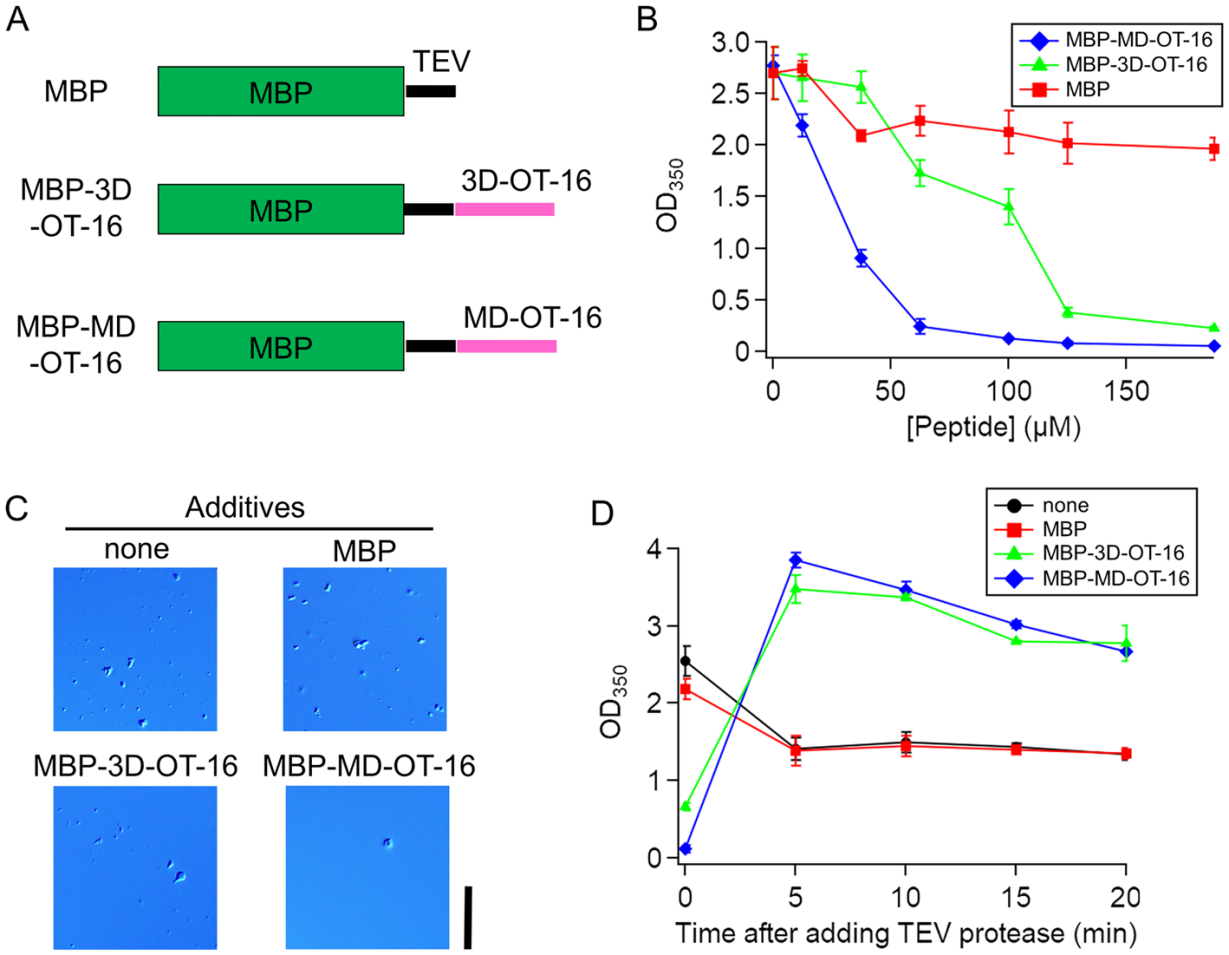
Soluble MBP-tag converts the designed peptide from an accelerator to a suppressor for p53 droplet formation. (**A**) Schematic illustration of primary sequences of the MBP-fused designed peptides and MBP used in this study. TEV represents the TEV protease cleavage sequence. The thick and thin bands correspond to folded and disordered regions, respectively. (**B**) Effect of the MBP-fused designed peptides or MBP as additives on p53 droplet formation detected as OD_350_. (**C**) DIC images of p53 solutions in the presence and absence of 125 µM MBP-fused designed peptides and MBP. Scale bar, 20 µm. These images were taken after 5 min of incubation of the p53 solutions with additives or without additive (none). (**D**) Time course of scattering (OD_350_) from 12.5 µM p53 solutions after incubation in the presence of 125 µM MBP-fused designed peptides or MBP for 5 min and the subsequent addition of TEV protease. The data before adding TEV protease are plotted at 0 min. “None” denotes the absence of MBP-fused designed peptide or MBP additives as control. In (**B** and **D**), error bars represent standard error (*N* = 3).

The maltose binding protein (MBP)-solubility tag was fused to the N-terminal of the designed 16-residue peptides: MBP-3D-OT-16 and MBP-MD-OT-16 (Fig. 4A). MBP-MD-OT-16 decreases the light scattering (OD_350_) of p53 droplets to less than 0.5 at 62.5 µM (blue in Fig. 4B), whereas MBP-3D-OT-16 decreases their light scattering to less than 0.5 at 125 µM (green in Fig. 4B). The high droplet-suppression ability of MBP-MD-OT-16 is attributed to its high affinity for p53 achieved by MD-based energy design. In contrast, MBP itself did not significantly affect light scattering, that is, p53 droplet formation (red in Fig. 4B). Consistent with the scattering data, MBP-MD-OT-16 reduced the droplet number more efficiently than MBP-3D-OT-16. Figure 4C shows the DIC images of the p53 droplet, which indicates that the number of droplets was reduced to 3% in 125 µM MBP-MD-OT-16 and to 36% in 125 µM MBP-3D-OT-16 compared with that in MBP (Supplementary Fig. S8). Furthermore, we confirmed that these MBP-fused designed peptides can collapse the formation of p53 droplets that have been formed (Supplementary Fig. S9). The droplet suppression effect of MBP-MD-OT-16 was thus demonstrated to be greater than that of MBP-3D-OT-16. Thus, the soluble tag fusion approach is useful for converting the designed peptides from droplet accelerators to suppressors.

As described above, the peptides designed with the MD-based energy have the ability to promote droplet formation, whereas the MBP-fused ones have the ability to suppress droplet formation. Accordingly, the removal of the MBP domain of the MBP-fused peptides should lead to the restoration of the droplet-forming effect of the peptides, as shown in Fig. 3. The MBP domain was removed using tobacco etch virus (TEV) protease. Droplet formation of p53, which was suppressed by the MBP-fused peptides, was promoted by the addition of TEV protease within 5 min (Fig. 4D). The DIC images demonstrated that the p53 droplet number was increased 18-fold in 125 µM MBP-MD-OT-16 and 3.0-fold in 125 µM MBP-3D-OT-16 by the addition of TEV protease (Supplementary Fig. S10). Accordingly, the MBP-fused designed peptides function as regulators of droplet formation, which are triggered by TEV protease.

#### Reduced interaction approach

We prepared four R-to-K peptides, which replaced R with K, but did not modify the other residues (Supplementary Fig. S11A). These R-to-K peptides showed 2.3- to 4.8-fold weaker affinity to the p53 N-terminal peptide than the original designed peptides, as seen in the increase in the dissociation constants upon R-to-K replacement (Supplementary Fig. S11B). However, contrary to the above assumption, light scattering from p53 droplets increased upon the addition of R-to-K peptides (Supplementary Fig. S11C). The DIC images demonstrated micrometer-sized p53 droplets with a similar relative distribution of cross-sectional area and similar circularity in the presence of every R-to-K peptide (Supplementary Fig. S11D,E). The p53 droplet number was increased in every R-to-K peptide (Supplementary Fig. S11F). This phenomenon may be ascribed to the bridging of p53 molecules via the K residues of the peptides, thereby promoting droplet formation, as reported previously^16^. In other scenario, the reduced electrostatic repulsion between p53 molecules (− 3.5 of the net charge per monomer) upon the positively-charged peptide binding might enhance droplet formation, since R-to-K replacement does not alter the charge of peptides. Thus, the reduced interaction approach did not attain the conversion to the droplet suppressor in contrast to the soluble tag fusion approach.

## Discussion

We here evaluated the MD-based contact energy for amino acid residue pairs without backbone steric constraints. The MD-based energy is consistent with the experimental data—parameters of solubility of aromatic amino acids in amino acid solvents (PSAS), which partly reflects the interactions between amino acids as a solubility^34^. The MD-based energy showed higher correlations with PSAS than the 3D structure-based energy (Supplementary Table S2). More specifically, in the MD-based energy, attractive interactions between positively charged residues (R and K) and aromatic residues (F, W, and Y) are more favorable than in the 3D structure-based energy, which accounts for the higher correlations with PSAS. The less favorable interactions in the 3D structure-based energy are ascribed to the fact that cation–π interactions between the positive charge and aromatic rings are not abundant in folded protein structures. Here, it should be noted that the R residue showed stronger interactions with aromatic residues than the K residue in the MD-based energy, which is consistent with a previous study^35^. This phenomenon can be attributed not only to cation–π interactions but also to π–π interactions via the π-bonded guanidium group of the R residue^11,36^, which lacks a K residue. Note that the cation–π and π–π interactions are not explicitly included in the force field, but they are implicitly described in terms of the electrostatic and van der Waals interactions^30,37^. The Aromaphilicity index also supports the strong interactions of R residues with F, W, and Y^27^. Accordingly, MD-based energy could successfully describe the intra- or inter-molecular interactions of disordered proteins, as well as the IDRs of proteins.

One potential application of MD-based energy is the design of peptides that bind to the IDRs of target proteins and regulate their functions. As an example, we demonstrated that the designed peptides target the droplet-forming IDR of p53 and regulate its LLPS behavior. The MD-based energy can thus be used to design strong peptide binders to target proteins; the advantage of this methodology is attributable to the cation–π interactions described in the MD-based energy, which are underestimated in the 3D structure-based energy (Fig. 2B). The importance of cation–π interactions was also reported in an experiment-guided design of peptides targeting FUS^16^. Accordingly, the present rational design methodology with MD-based energy would be a good fit especially for R- or aromatic residue-rich regions of target disordered proteins.

In this study, the soluble tag fusion approach for designed peptides showed an effective ability to regulate the LLPS of target proteins. In fact, the soluble MBP tag converted the designed peptide from the accelerator to a suppressor for droplet formation of p53 (Fig. 4). As a result of MBP-tag fusion, the solubility of the designed peptide–target protein complex is enhanced and the association between LLPS-related proteins is sterically hindered, resulting in the collapse of the droplets.

## Conclusion

We provide a rational design methodology for artificial LLPS-regulators based on virtual screening using the MD-based contact energy between amino acid residues. The MD-based contact energy is effective for describing the intermolecular interactions between peptides and disordered proteins, which enables the identification of strong peptide binders to IDRs of LLPS-related proteins. This methodology could be applied to various LLPS-related proteins of interest for biological and therapeutic purposes.

## Materials and methods

### MD simulation

To investigate the binding affinity between all amino acid pairs, MD simulations were conducted using the GROMACS 2016 simulator^38^. Amino acids (whole, not truncated) and amino acid analog molecules similar to the side chain group were used (Supplementary Text 1). Both were described using the AMBER99SB force field^39^ with restrained electrostatic potential (RESP) charges^40^. Water molecules were described using the TIP3P model^41^. Sodium and chloride ion models developed by Joung and Cheatham were used^42^. The system contained two molecules, amino acids or amino acid analogs and 2500–2600 water molecules. When the system contained charged amino acids, and the net charge of the system was not zero, chloride ions or sodium ions were added to the systems to neutralize the net charge of the system. These molecules were placed in a dodecahedron box with 47-Å sides.

MD simulations of amino acid pairs were performed using another force field. Truncated amino acid molecules, similar to the side-chain group, were used, described by the CHARMM22 force field^30,31^ (Supplementary Text 2) and the OPLS-AA/L force field^32^ (Supplementary Text 3). Similarly, the water molecules were described using the TIP3P model modified for CHARMM^30^ and TIP3P model for OPLS-AA/L^41^. The ion parameters for CHARMM were determined according to the model established by Beglov and Roux^43^. The ones for OPLS described by Chandrasekhar et al. were used^44^. All other conditions were the same as those described previously.

Umbrella sampling simulations were performed to determine the binding free energy profiles of these systems, as conducted in previous studies^45–49^. In general, in umbrella sampling simulations, changes in the binding free energy (A(ξ)) along the order parameter (ξ) were acquired by combining the potential mean force (PMF) along ξ. A series of bias potentials was applied to the MD simulation to efficiently sample the entire order parameter range. The relevant order parameter ranges were divided into bins. Each bias potential (wi (ξ)) is assigned to a window. Thus, the MD simulations generate the PMF for the biased system as follows:

1 $$A_{i}^{b} (\xi ) = - \frac{1}{\beta }\ln P_{i}^{b} (\xi )$$

where *β* = 1/k_B_*T*, k_B_ and *T* are the Boltzmann constant and absolute temperature, respectively, and the suffix *b* denotes “biased”. The PMF of an unbiased system in each window is represented by the following equation:

2 $$A_{i}^{u} (\xi ) = - \frac{1}{\beta }\ln P_{i}^{b} (\xi ) - w_{i} (\xi ) + F_{i} ,$$

where *F*_*i*_ is a constant and the suffix *u* denotes “unbiased”.

In the present study, umbrella integration (UI) was used to combine the PMFs of biased systems^50,51^. The unbiased PMF in each UI bin was calculated from the biased PMF derivative using the following equation:

3 $$\frac{{\partial A_{i}^{u} (\xi )}}{\partial \xi } = - \frac{1}{\beta }\frac{{\partial \ln P_{i}^{b} (\xi )}}{\partial \xi } - \frac{{dw_{i} (\partial \xi )}}{d\xi }$$

Kästner and Thiel showed that, given that the restraint potential has a harmonic formula, the following equation can be obtained^50,51^:

4 $$w_{i} (\xi ) = \frac{1}{2}K\left( {\xi - \xi_{i}^{c} } \right)^{2}$$

which is the center of the window. In addition, it is approximated based on a normal distribution. Therefore, Eq. (3) can be rewritten as follows:

5 $$\frac{{\partial A_{i}^{u} (\xi )}}{\partial \xi } = - \frac{1}{\beta }\frac{{\xi - \overline{\xi }_{i}^{b} }}{{\left( {\sigma_{i}^{b} } \right)^{2} }} - K\left( {\xi - \xi_{i}^{c} } \right)$$

which is the mean of the biased simulation in window (*i*), and *σ* is the variance. In this study, we defined the order parameter, *ξ*, as the distance between the center of mass (COM) of amino acids. Umbrella sampling was conducted for *ξ* = 3.0–14.5 Å, which was divided into 24 bins with a window length of 0.5 Å, using a spring constant of *K* ≈ 239 kcal/mol/Å^2^ (≈ 1,000 kJ/mol/Å^2^). 5-ns MD simulations were performed in each window, wherein the last 3 ns of the data were used to determine the binding free energy (corresponding to *e*_*ij*_ for the *i*th and *j*th amino acids in the peptide design section). Four independent simulations were performed for each system. The total simulation time was as follows: 210 (all amino acid pairs) × 24 (bins) × 5 (ns) × 4 (runs) = 100,800 ns.

### Peptide design

Peptides were designed as previously described^15^. Briefly, the relative binding free energy between the *i*th residue of the N-terminal domain and the *j*th residue of the designed peptide was calculated as *e*_*ij*_ + *e*_*rr*_ − *e*_*ir*_ − *e*_*jr*_. For the MD-based design, *e*_*ir*_ (*e*_*jr*_) and *e*_*rr*_ were calculated as $\sum_{j=1}^{20}{e}_{ij}/20$ and $\sum_{i=1}^{20}{e}_{ir}/20$, respectively. For the 3D-based design, *e*_*ir*_ (*e*_*jr*_) and *e*_*rr*_ were obtained from a previous study^18^. We calculated the binding free energy by replacing the *j*th residue of a designed peptide with each of the 20 residues, and then determined the residues that gave the lowest binding free energy. For MD-based design, we used the energy of the side chains, and Ala was selected in the case of either Ala or Gly. This procedure was repeated to obtain the 10-residue or 16-residue peptide sequence, and the total binding energy was calculated by summing the binding energies. A designed peptide with minimal total energy was selected among peptides designed for different initial residues of the N-terminal domain (Supplementary Fig. S2). Since the MD-OT sequences with minimum contact energy were all R residues, we chose the sequence with the binding energy close to the minimum and the variety of residues to some extent (Supplementary Fig. S2). For one-by-three design calculations, we added the binding energy between adjacent residues of the N-terminal peptide and each residue of the designed peptide.

### Samples

We used a thermostable and cysteine-modified mutant of human p53 (C124A, C135V, C141V, W146Y, C182S, V203A, R209P, C229Y, H233Y, Y234F, N235K, Y236F, T253V, N268D, C275A, C277A, and K292C)^33^. We confirmed the affinity of the p53 mutant for DNA^52^, and the expression and purification of p53 were conducted as previously described^33^. For the titration and droplet-regulation experiments, N-terminal peptide (residues 37–65 of human p53) labeled with FAM at the N-terminus and designed peptides were synthesized without caps and obtained with at least 95% purity (Biologica Co.). For peptide uptake measurements, 3D-OT-16 with N-terminal cysteine and MD-OT-16 were synthesized using a standard Fmoc-based solid-phase peptide synthesis. These peptides were labeled with Alexa488 via maleimide and succinimidyl ester chemistry, respectively. For testing non-specific interaction of designed peptides, C-terminal peptide (residues 367–393) of human p53^15^ was also synthesized and labeled with 5-FAM at the N-terminus. The purity and identity of each peptide were verified using HPLC and mass spectrometry (Autoflex-T1, Bruker Daltonics). Genes containing MBP-3D-OT-16, MBP-MD-OT-16, and MBP were prepared from the pTHMT plasmid containing 6 × His-MBP-TEV-FUS (98651; Addgene), where TEV denotes the cleavage sequence of the TEV protease. The MBP-3D-OT-16 and MBP-MD-OT-16 genes were constructed by removing the FUS sequence using NdeI and XhoI and ligating with designed peptide sequences. The MBP gene was constructed by removing the FUS sequence using the KOD-Plus-Mutagenesis Kit (Toyobo). MBP-3D-OT-16, MBP-MD-OT-16, and MBP containing 6 × His at the N-terminus and TEV sequences were expressed and purified as described previously^16^.

### Titration experiments

The fluorescence anisotropy of the fluorescent N-terminal and C-terminal peptide of p53 was measured at 25 °C using a fluorescence spectrometer (FP-6500; JASCO Co., Tokyo, Japan) with an automatic titrator and a home-built autorotating polarizer^33^. Non-labeled peptides were titrated into a solution containing 10 nM FAM-labeled N-terminal peptide or 20 nM FAM-labeled C-terminal peptide, 25 mM HEPES, 0.5 mM EDTA, 1 mM DTT, 2 mM trolox, and 0.2 mg/mL BSA (pH 7.0). The titration curves were fitted using the following equations based on one to one binding model:

6 $${r}_{\text{obs}}={r}_{\text{A}}\frac{\left({c}_{\text{A}}-{c}_{\text{AB}}\right)}{{c}_{\text{A}}}+{r}_{\text{AB}}\frac{{c}_{\text{AB}}}{{c}_{\text{A}}},$$

7 $${c}_{AB}=\frac{\left({c}_{\text{A}}+{c}_{\text{B}}+{K}_{D}\right)-\sqrt{{\left({c}_{\text{A}}+{c}_{\text{B}}+{K}_{D}\right)}^{2}-4{c}_{\text{A}}{c}_{\text{B}}}}{2},$$

where *r*_obs_, *r*_A_, *r*_AB_, *K*_D_, *c*_A_, and *c*_B_ are the observed anisotropy, anisotropy of free molecule A, anisotropy of the complex between molecules A and B, dissociation constant, total concentration of molecule A, and total concentration of molecule B, respectively. The analysis was performed using Igor software.

### Sample solutions for the droplet formation experiments

For the static measurement of p53 droplet regulation, we used solutions containing 12.5 µM p53, 25 mM HEPES, 45 mM NaCl, 0.5 mM EDTA, 1 mM DTT, and various concentrations of designed peptides at pH 7.0. These solutions were prepared by 10-fold dilution of a 125 µM p53 stock solution containing 450 mM NaCl at pH 7.5. After 5 min of incubation at 20 °C, the scattering and DIC measurements were conducted. For kinetic measurements with initial p53 droplet formation and the subsequent addition of additives, we incubated p53 solution containing 20.8 µM p53, 25 mM HEPES, 75 mM NaCl, 0.5 mM EDTA, and 1 mM DTT at pH 7.0 for 5 min at 20 °C. Then, the p53 and additive solutions were mixed resulting in 12.5 µM p53, 100 µM MBP-fused designed peptides or MBP, and 45 mM NaCl, which were incubated for 5 and 10 min at 20 °C. For MBP-tag cleavage measurements, we incubated p53 solution containing 12.5 µM p53, 25 mM HEPES, 45 mM NaCl, 0.5 mM EDTA, 1 mM DTT, and 125 µM MBP-fused designed peptides or MBP at pH 7.0 for 5 min at 20 °C. Then, the p53 and TEV protease solutions were mixed resulting in 11 µM p53, 110 µM MBP-fused designed peptides or MBP, 40 mM NaCl, and 250 µg/mL TEV protease (TurboTEV Protease; Accelagen) and were incubated for 5–20 min at 20 °C.

### Scattering measurements

Scattering from p53 solution with or without designed peptides was measured as OD_350_ at 21 °C using an absorbance spectrometer (Nano Drop One; Thermo Fisher). The optical path length was set to 1 mm. The OD_350_ values were displayed for a path length of 10 mm. No significant increase of OD_350_ was confirmed for only designed peptides.

### DIC microscopy

We used inverted microscopes (IX-73; Olympus, Tokyo, Japan) equipped with a microscopic objective (60×) and a camera (DP73 or DP74), as described previously, with some modifications^8^. The sample solution was cast on a coverslip (Matsunami Glass) and covered with a glass slide (Matsunami Glass). The coverslip and slide glass were cleaned with ethanol and 5 M KOH before use. DIC images were obtained at 21 °C. The cross-sectional area and circularity of the droplets were calculated using ImageJ software^16^.

## Supplementary Information

Supplementary Information.

## Data Availability

All data generated or analyzed during this study are included in this published article and its Supplementary Information.
