# Supplementary material for: Rational peptide design for regulating liquid–liquid phase separation on the basis of residue–residue contact energy
Source: Sci Rep. 2022 Aug 12;12:13718. doi: 10.1038/s41598-022-17829-1 (PMC9374670; doi:10.1038/s41598-022-17829-1)
Supplement: Supplementary file 1 — Supplementary Information. [file 41598_2022_17829_MOESM1_ESM.docx]

Supplementary Information

Rational peptide design for regulating liquid–liquid phase separation on the basis of residue–residue contact energy

Kiyoto Kamagata^1,2,3^*, Maulana Ariefai^1,2^, Hiroto Takahashi^1^, Atsumi Hando^1,3^, Dwiky Rendra Graha Subekti^1^, Keisuke Ikeda^4^, Atsushi Hirano^5^, and Tomoshi Kameda^6^*

**
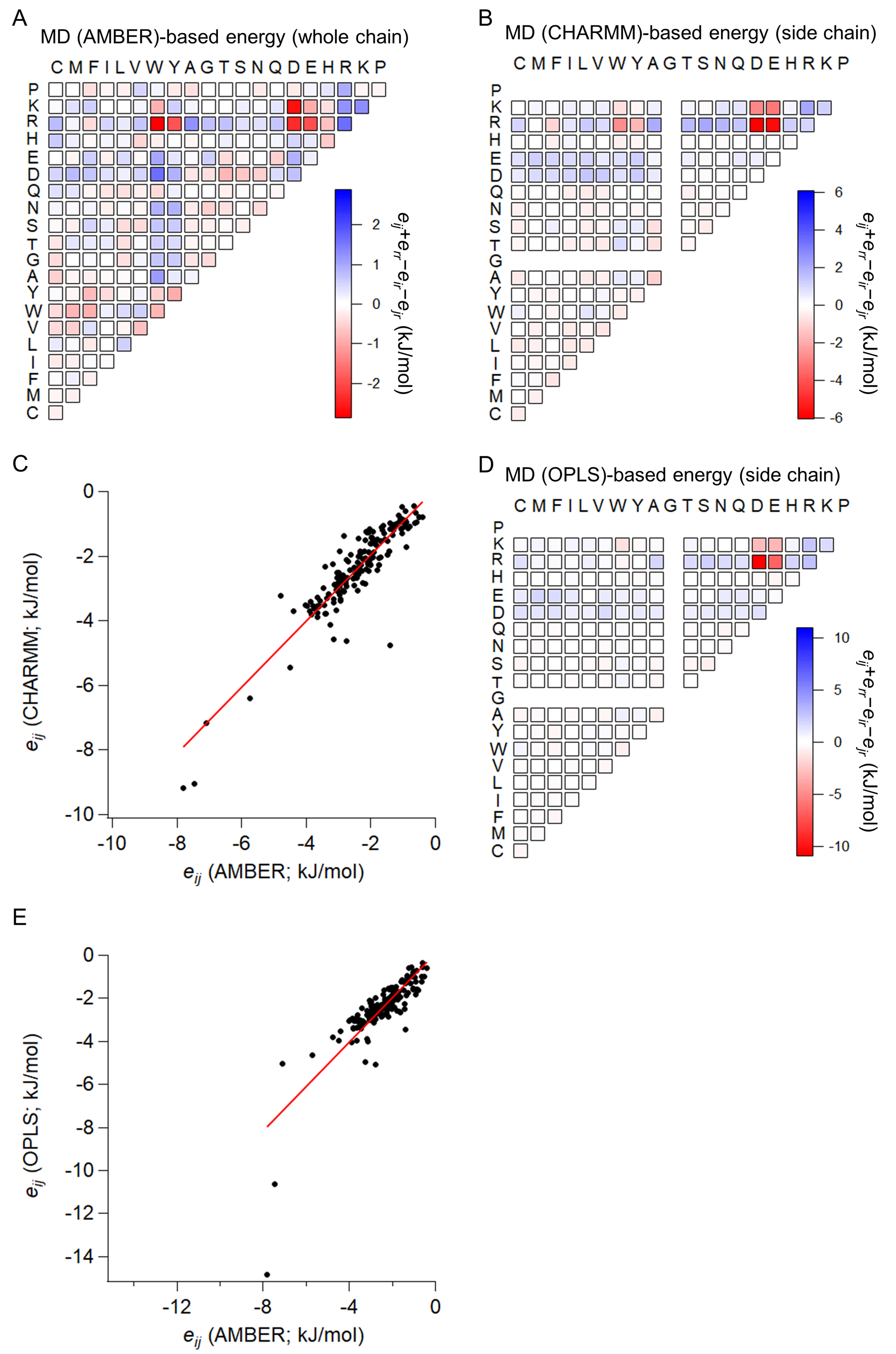
**

**Fig. S1.** (A) MD (AMBER)-based relative contact energy matrix for whole amino acids. (B) MD (CHARMM)-based relative contact energy matrix for side chains. Eighteen residues, except for Pro and Gly residues, were used to calculate *e_i_*_r_, *e*_r_*_j_*, and *e*_rr_. Note that the data for Pro and Gly residues are blank in CHARMM. (C) Comparison of contact energy (*e_ij_*) for side chains between AMBER and CHARMM force fields. The red line denotes the best-fitted line for the data (*r* = 0.87). (D) MD (OPLS)-based relative contact energy matrix for side chains. Eighteen residues, except for Pro and Gly residues, were used to calculate *e_i_*_r_, *e*_r_*_j_*, and *e*_rr_. Note that the data for Pro and Gly residues are blank in OPLS. (E) Comparison of contact energy (*e_ij_*) for side chains between AMBER and OPLS force fields. The red line denotes the best-fitted line for the data (*r* = 0.82).


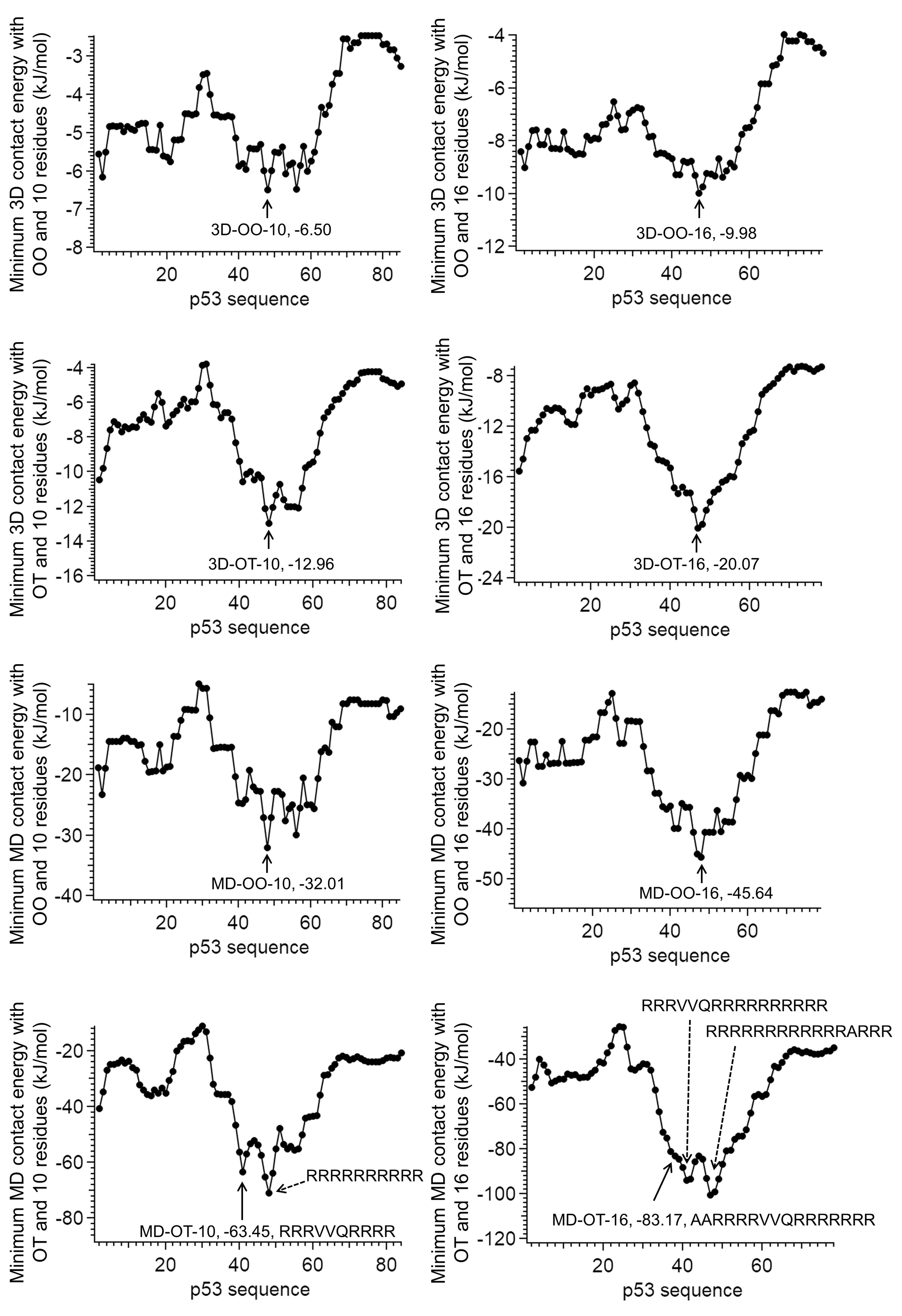


**Fig. S2.** Designed peptides were selected from the candidates targeting partial p53 sequences based on the 3D structure-based (3D) or MD-based (MD) energy considering one-by-one (OO) or one-by-three (OT) interactions. Minimum contact energy (*y*-axis) of the 10-residue or 16-residue peptide, which is complementary to p53, is plotted against the p53 sequence number (*x*-axis). The p53 sequence number (*x*-axis) corresponds to the initial residues of the complementary peptide candidates with minimum contact energy. For example, the plot at *x* = 48 for 3D-OO-10 represents the minimum contact energy (*y* = −6.50) among those of 10-residue peptide candidates (i.e., 20^10^ candidates) targeting 48^th^–57^th^ residues of the p53 sequence, which is based on the 3D structure-based energy considering OO interactions. The solid arrows indicate the designed peptides used in this study. In MD-OT-10, the peptide sequence corresponding to the global minimum of the contact energy landscape was RRRRRRRRRR (*x* = 48, *y* = −71.03). Because the single amino-acid repeats might decrease the interaction specificity of the peptides for the IDR regions of p53 due to the increased chance of unexpected interactions with other regions of p53, we chose the peptide with second minimum (RRRVVQRRRR) (*x* = 41, *y* = −63.45) as a designed peptide. In MD-OT-16, the peptide sequence corresponding to the global minimum of the energy landscape was RRRRRRRRRRRRARRR (*x* = 47, *y* = −100.45), and that corresponding to the second minimum was RRRVVQRRRRRRRRRR (*x* = 41, *y* = −94.12). Considering that the major component percentage (i.e., R in the case of MD-OT-16) should not exceed 70%, we chose AARRRRVVQRRRRRRR (*x* = 38, *y* = −83.17), which was located in the shoulder of the second minimum valley of the energy landscape.


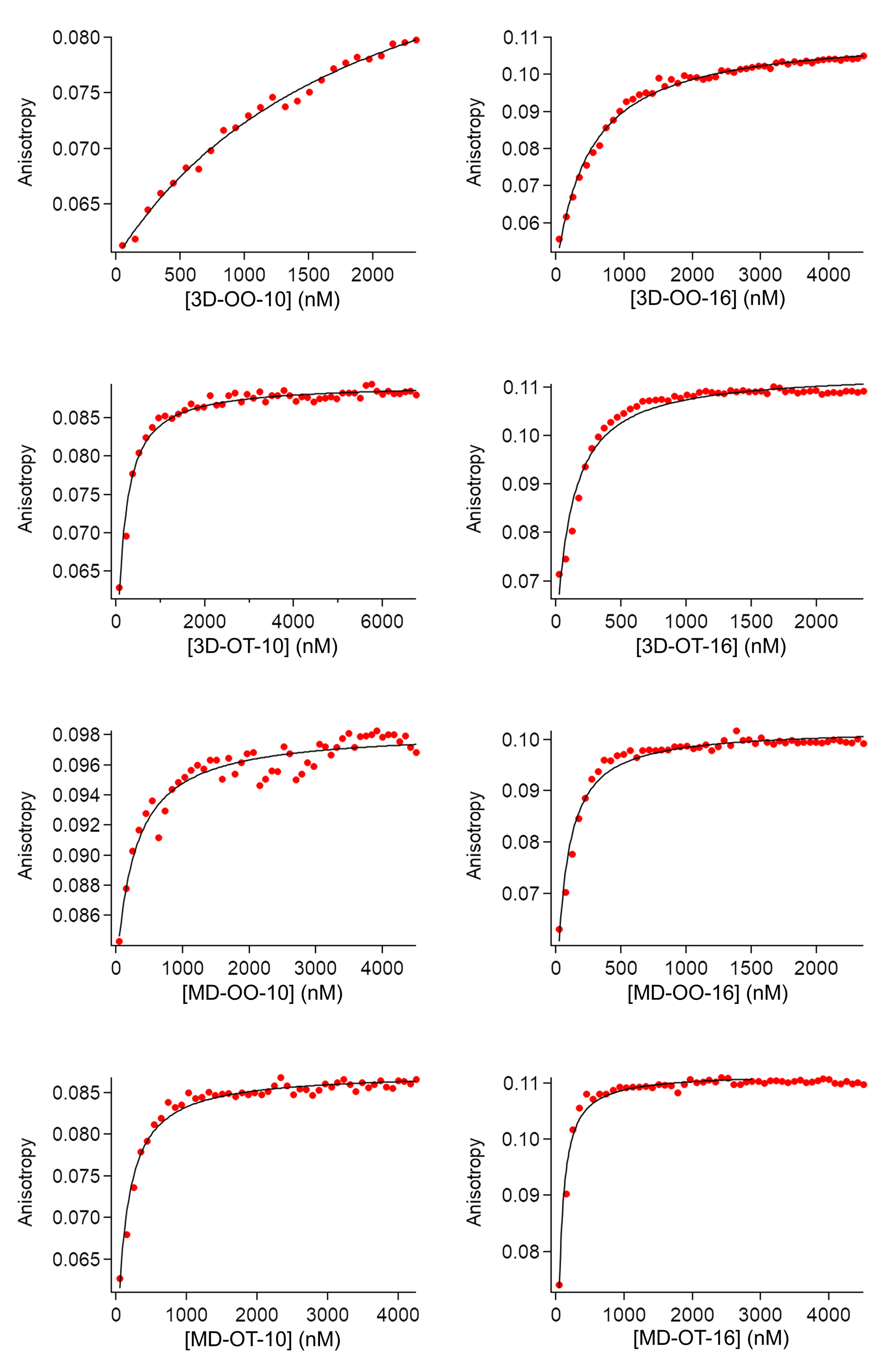


**Fig. S3.** Titration of designed peptides against the N-terminal domain of p53. Fluorescence anisotropy changes of the N-terminal peptide labeled with FAM were monitored upon the addition of the peptides. The black curves denote best fitted curves based on the one-to-one binding model.


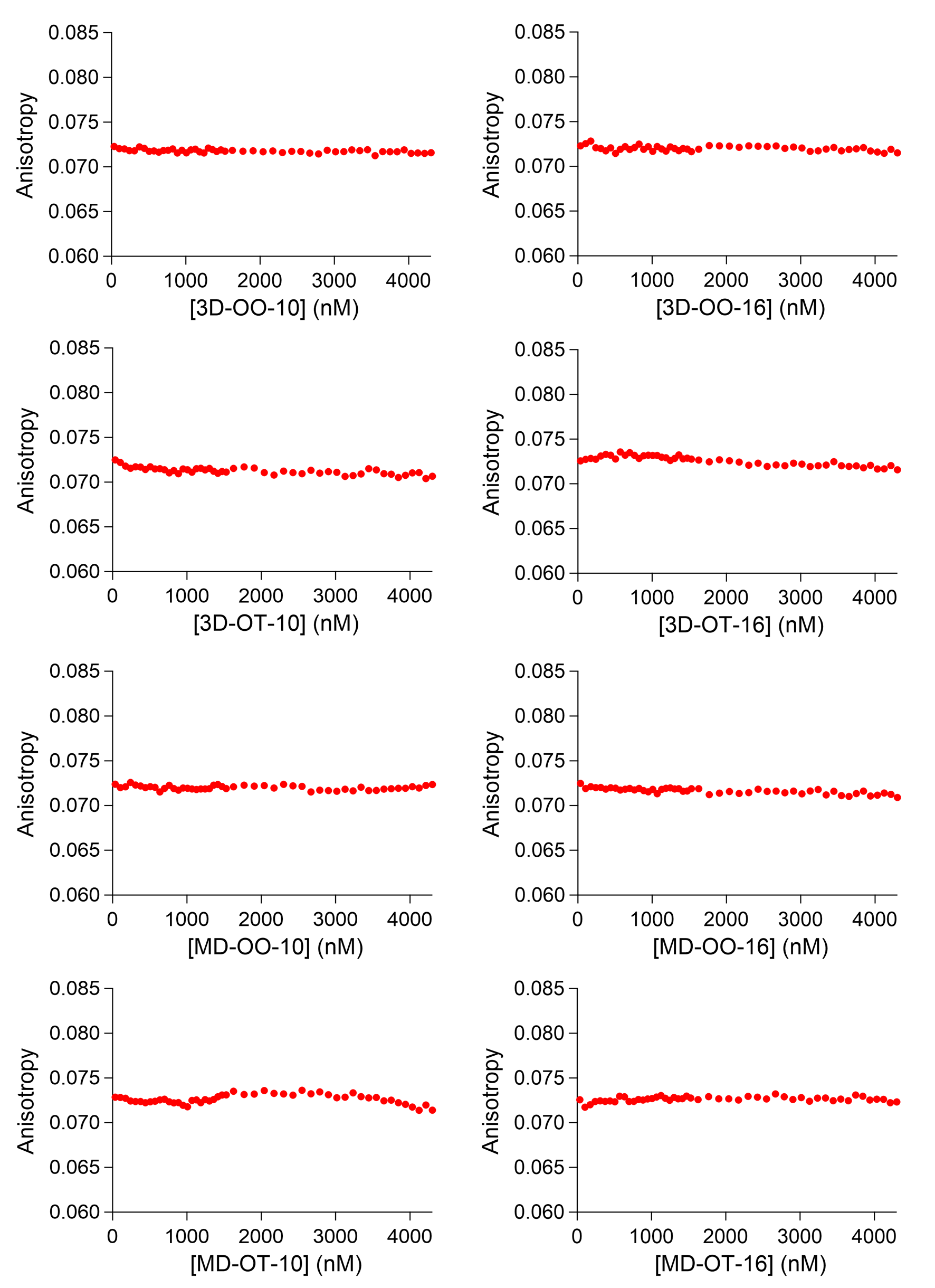


**Fig. S4.** Titration of designed peptides against the C-terminal domain of p53. Fluorescence anisotropy changes of the C-terminal peptide labeled with FAM were monitored upon the addition of the peptides.


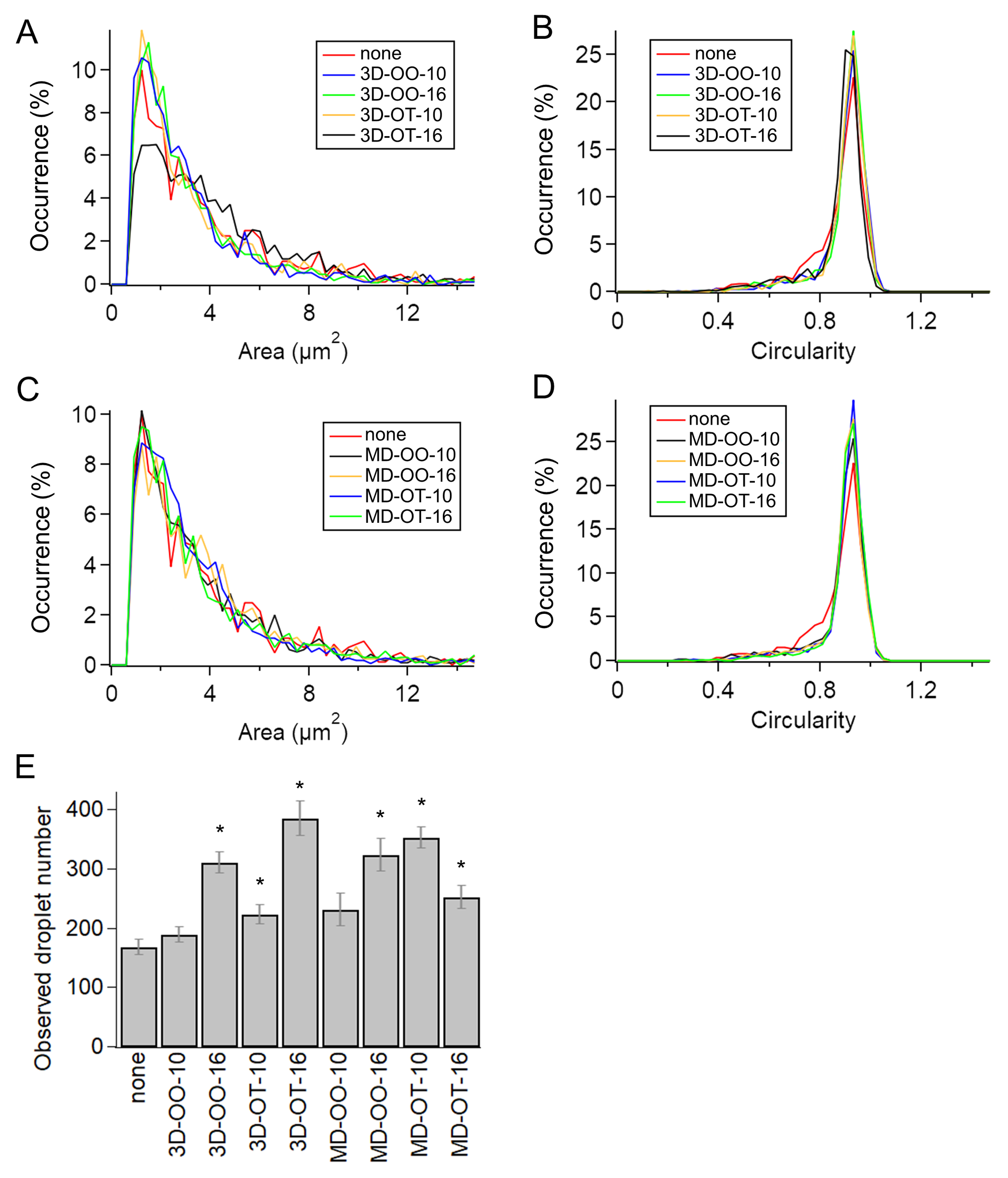


**Fig. S5.** (A-D) Distributions of cross-section area and circularity of individual p53 droplets in the presence and absence of 250 µM designed peptides. (E) Average droplet numbers in DIC images in the presence and absence of 250 µM designed peptides. “None” denotes the absence of designed peptides as a control. Error bars denote standard errors (*N* = 5). Asterisks indicate a significant difference between the presence and absence of designed peptides in two-tailed t test (*p* = 0.0004 for 3D-OO-16, *p* = 0.03 for 3D-OT-10, *p* = 0.0006 for 3D-OT-16, *p* = 0.002 for MD-OO-16, *p* = 0.00007 for MD-OT-10, *p* = 0.008 for MD-OT-16).


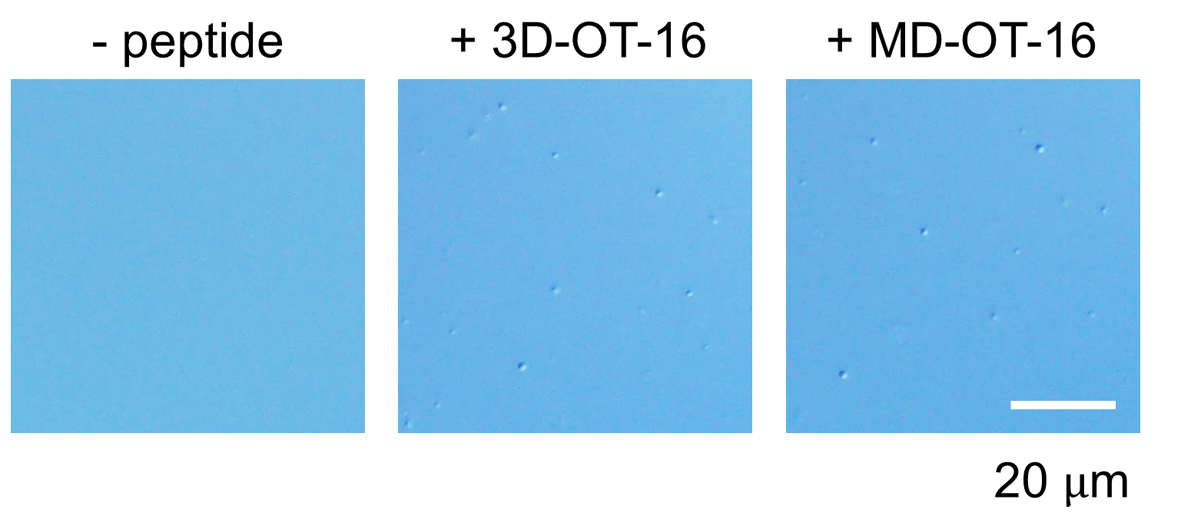


**Fig. S6.** DIC images of p53 below the critical concentration in the presence and absence of designed peptides. The solution contained 25 mM HEPES, 0.5 mM EDTA, 4.5 mM NaCl, 1 mM DTT, 1.25 µM p53, and 0 or 250 µM designed peptides at pH 7.0. The images were taken after 5 min of incubation of the p53 solution at 21℃.


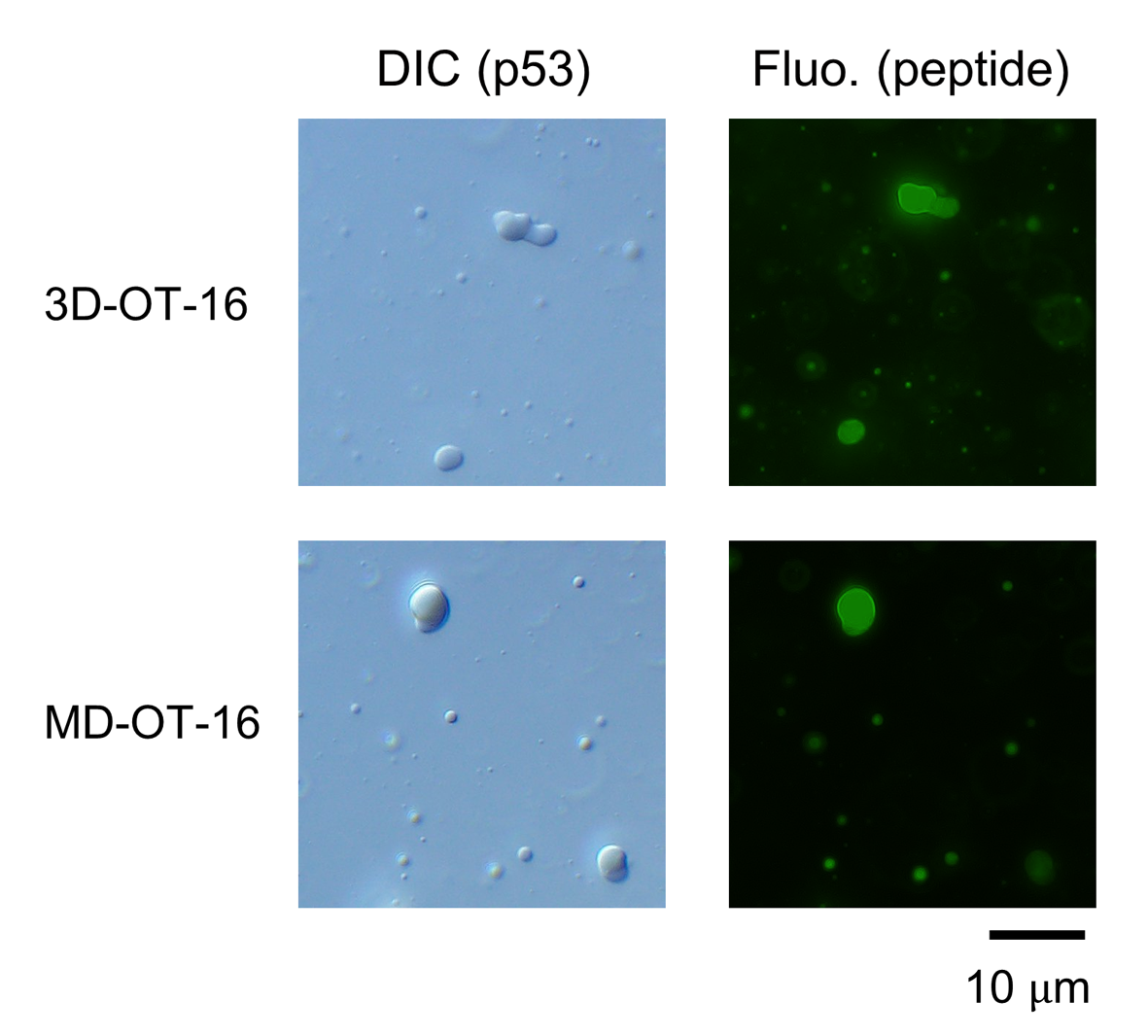


**Fig. S7.** DIC and fluorescence microscopic images of uptake of the designed peptides into p53 droplets. The solution contained 25 mM HEPES, 0.5 mM EDTA, 4.5 mM NaCl, 1 mM DTT, 150 mg/mL dextran (MW 45,000– 65,000; Sigma-Aldrich), 12.5 µM p53, and 100 nM Alexa488-labeled designed peptides at pH 7.0. The data was taken using the inverted microscope (IX-73; Olympus) at 21℃.


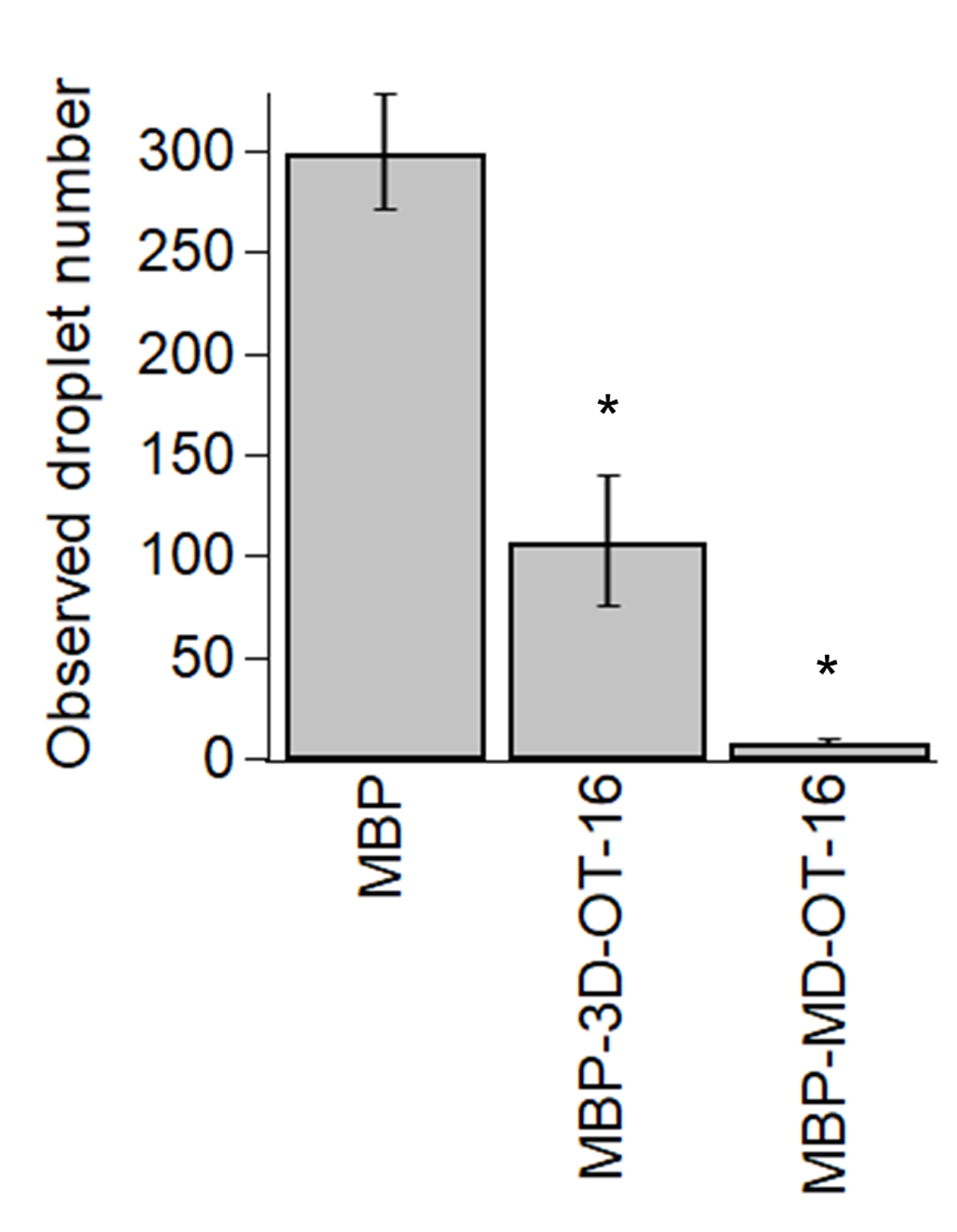


**Fig. S8.** Average droplet numbers in DIC images in the presence of 125 µM MBP or MBP-fused designed peptides. Error bars denote standard errors (*N* = 5). Asterisks indicate a significant difference between MBP and MBP-fused designed peptides in two-tailed t test (*p* = 0.002 for MBP-3D-OT-16, *p* = 0.0005 for MBP-3D-OT-16).

**
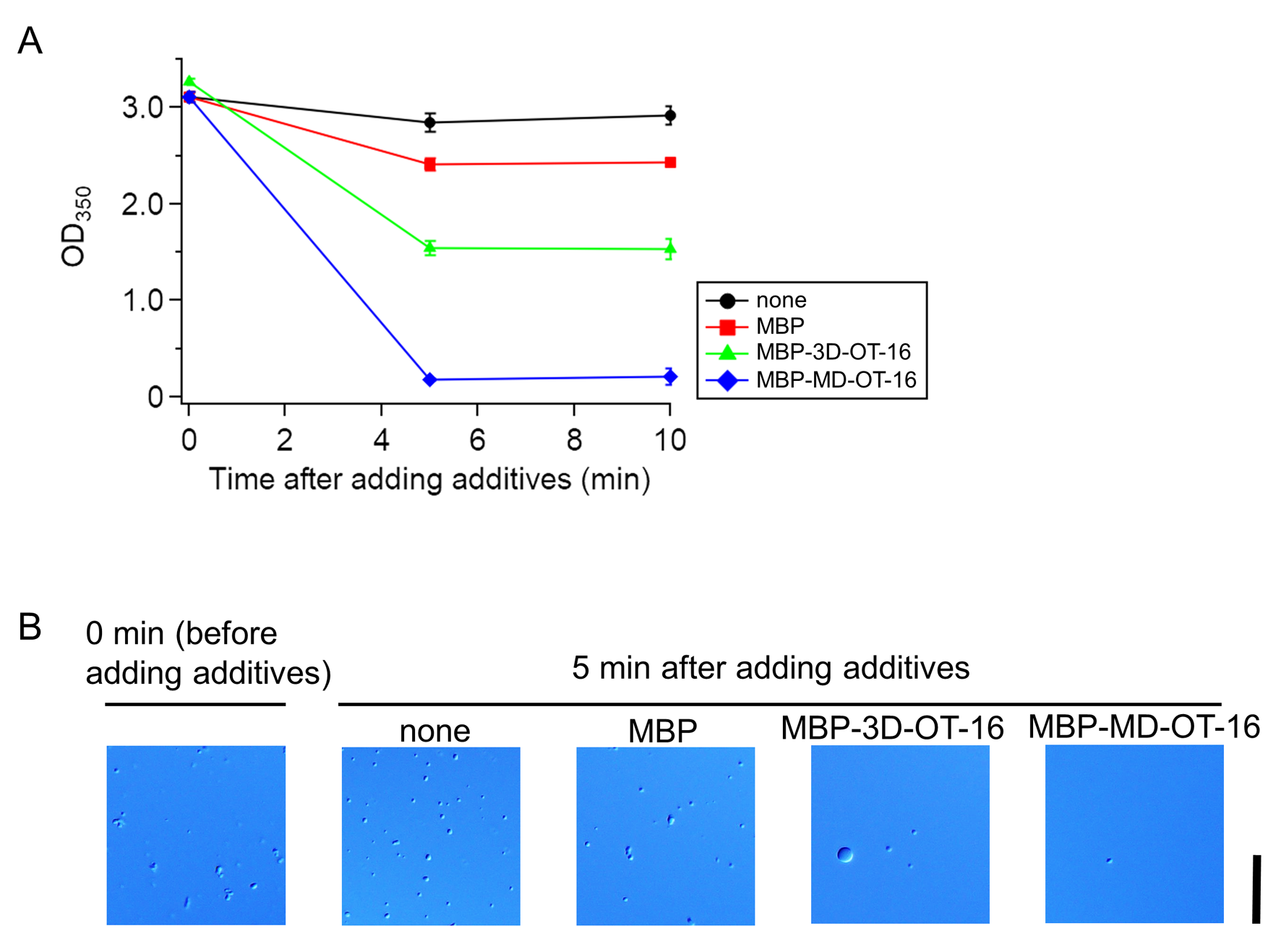
**

**Fig. S9.** (A) Time course of light scattering (OD_350_) from p53 solutions after the addition of 100 µM MBP-fused designed peptides or MBP to the solutions. The original solution containing droplets was prepared by 5 min of incubation of the p53 solutions without additives; the data of which are plotted at 0 min. “None” denotes the absence of MBP-fused designed peptides or MBP additives as a control. The errors are the standard error (*N* ≥ 3). (B) DIC images of p53 solutions after the 5-min incubation in the presence and absence of 100 µM MBP-fused designed peptides or MBP. Scale bar, 20 µm.


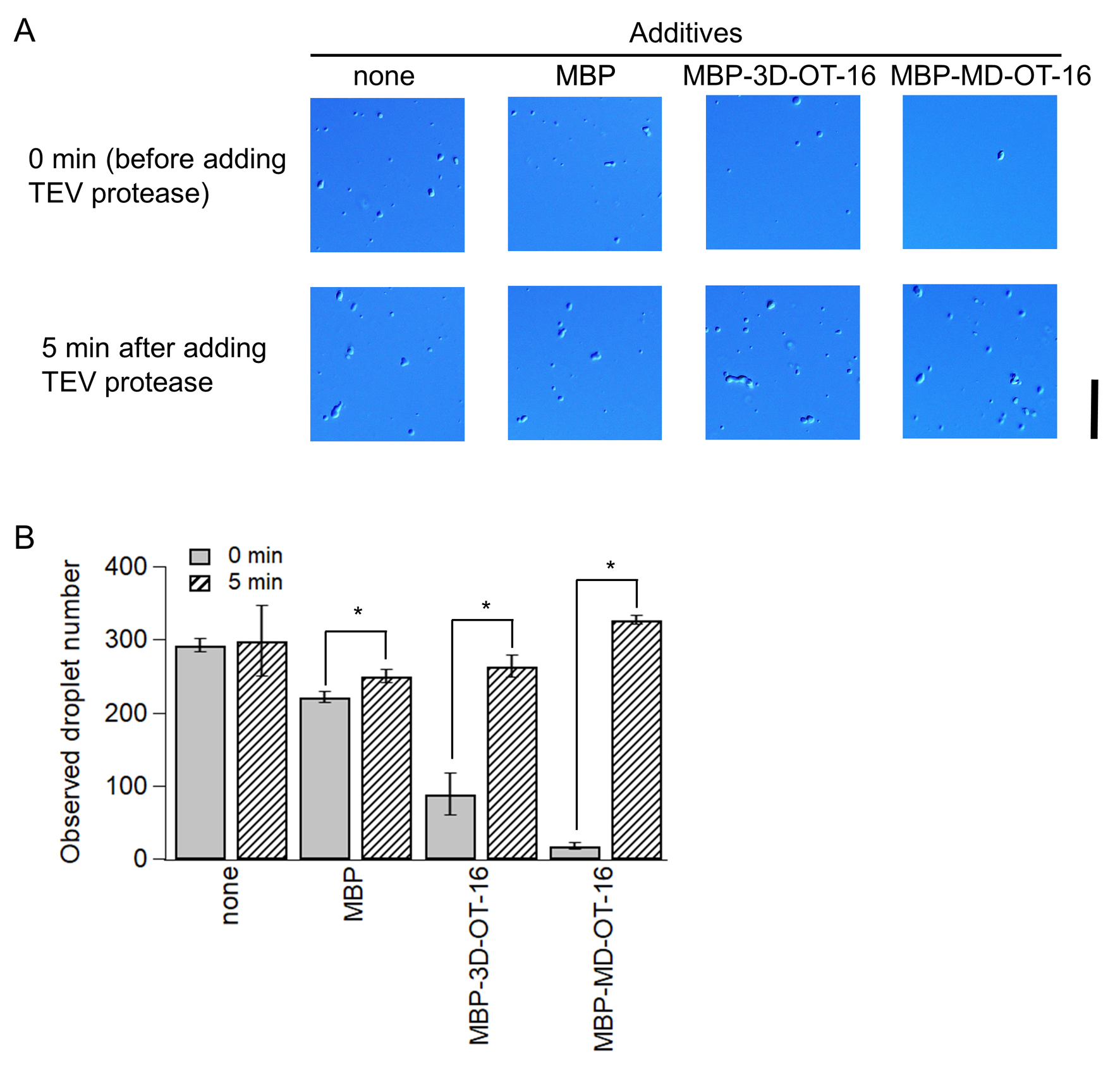


**Fig. S10.** (A) DIC images of p53 solutions after 5 min of incubation in the presence of 125 µM MBP-fused designed peptides or MBP (top) and the subsequent 5 min of incubation with TEV protease (bottom). “None” denotes the absence of MBP-fused designed peptides or MBP additives as a control. Scale bar, 20 µm. (B) Average droplet numbers in DIC images. Error bars denote standard errors (*N* = 5). Asterisks indicate a significant difference in two-tailed t test (*p* = 0.03 for MBP, *p* = 0.002 for MBP-3D-OT-16, *p* < 0.0001 for MBP-3D-OT-16).


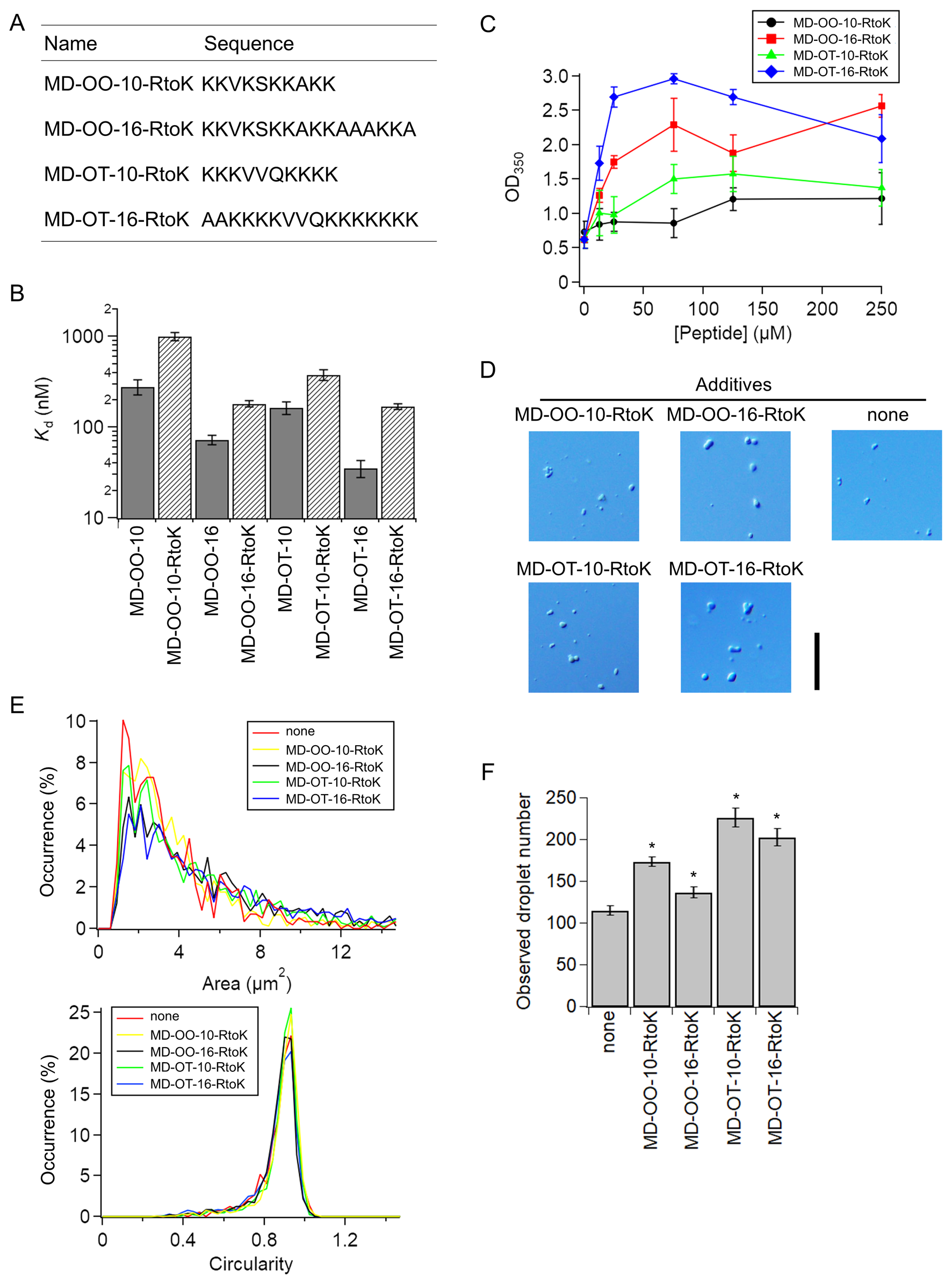


**Fig. S11.** Effect of R-to-K replacement for designed peptides on p53 droplets. (A) Sequence of R-to-K designed peptides used in this study. (B) Dissociation constants (*K*_d_) of the original designed peptides (grey) and the R-to-K designed peptides (shaded) for the p53 N-terminal peptide. *K*_d_ was determined by titrating the designed peptides against the p53 N-terminal peptide. Errors denote the fitting errors. (C) Effect of R-to-K designed peptides as additives on p53 droplet formation detected as OD_350_. The errors are the standard error (*N* ≥ 3). (D) DIC images of p53 solutions in the presence and absence of 250 µM R-to-K designed peptides. (E) Distributions of cross-section area and circularity of individual p53 droplets in the presence and absence of 250 µM R-to-K designed peptides. (F) Average droplet numbers in DIC images. Error bars denote standard errors (*N* = 5 or 6). Asterisks indicate a significant difference between the presence and absence of R-to-K designed peptides in two-tailed t test (*p* < 0.0001 for MD-OO-10-RtoK, *p* = 0.04 for MD-OO-16-RtoK, *p* = 0.0001 for MD-OT-10-RtoK, *p* = 0.0003 for MD-OT-16-RtoK).

**Table S1.** MD-based contact energy (*e_ij_*) for *i*th and *j*th residues (kJ/mol).

|  |  | AMBER | | CHARMM | OPLS |
| --- | --- | --- | --- | --- | --- |
| Residue | Residue | Side chain | Whole amino acid | Side chain | Side chain |
| C | C | −2.17904 | −1.92478 | −2.860153 | −2.85246 |
| C | M | −2.78557 | −1.66434 | −2.815358 | −2.96285 |
| C | F | −2.07131 | −1.69439 | −2.450573 | −2.43347 |
| C | I | −3.07283 | −1.79699 | −2.905789 | −2.61475 |
| C | L | −2.94957 | −1.32278 | −3.324938 | −2.82296 |
| C | V | −2.91358 | −2.07097 | −2.761142 | −2.82501 |
| C | W | −2.29893 | −2.87560 | −2.282286 | −2.05618 |
| C | Y | −2.23920 | −1.90857 | −2.839524 | −2.48621 |
| C | A | −2.44362 | −2.12923 | −2.658902 | −2.72009 |
| C | G | −2.44362 | −2.00976 | −2.658902 | −2.72009 |
| C | T | −2.25201 | −2.28725 | −2.077988 | −2.23997 |
| C | S | −1.74890 | −2.17174 | −2.073543 | −2.0235 |
| C | N | −2.14331 | −2.32682 | −2.408559 | −2.25635 |
| C | Q | −2.02872 | −1.48432 | −2.251309 | −2.48648 |
| C | D | −0.88802 | −1.81920 | −0.946924 | −0.73339 |
| C | E | −0.93326 | −1.58849 | −0.898835 | −1.02025 |
| C | H | −2.35508 | −1.30303 | −2.01908 | −2.42782 |
| C | R | −2.40396 | −1.78557 | −3.079118 | −2.78605 |
| C | K | −1.69258 | −1.71650 | −2.042579 | −1.91749 |
| C | P | −2.41110 | −1.50752 |  |  |
| M | M | −3.38118 | −1.58835 | −3.771733 | −3.23881 |
| M | F | −2.94140 | −1.41570 | −3.380862 | −3.01157 |
| M | I | −3.64020 | −1.55946 | −3.552952 | −3.03941 |
| M | L | −3.65723 | −1.33137 | −3.765397 | −2.88237 |
| M | V | −3.61291 | −1.98223 | −3.56988 | −2.77632 |
| M | W | −3.30376 | −3.10936 | −3.184715 | −3.14086 |
| M | Y | −3.40879 | −1.93880 | −3.774036 | −3.27666 |
| M | A | −2.46192 | −1.56745 | −2.635252 | −2.82107 |
| M | G | −2.46192 | −1.38147 | −2.635252 | −2.82107 |
| M | T | −2.44840 | −1.49344 | −2.377364 | −2.30293 |
| M | S | −2.41442 | −2.12528 | −2.197537 | −1.81004 |
| M | N | −2.54180 | −2.27205 | −2.6956 | −2.77749 |
| M | Q | −2.50619 | −1.38862 | −2.680841 | −2.87285 |
| M | D | −0.91324 | −1.38297 | −1.083361 | −1.21186 |
| M | E | −1.22834 | −1.57434 | −1.013799 | −0.61196 |
| M | H | −2.64525 | −1.55421 | −2.424977 | −2.61601 |
| M | R | −3.15663 | −2.25171 | −4.581517 | −3.88984 |
| M | K | −1.99843 | −1.26644 | −2.276882 | −1.95278 |
| M | P | −2.85824 | −1.40234 |  |  |
| F | F | −3.06682 | −1.98447 | −3.687001 | −3.03503 |
| F | I | −2.96207 | −1.49885 | −3.189209 | −2.96116 |
| F | L | −2.84502 | −1.89611 | −3.074968 | −3.13778 |
| F | V | −2.76432 | −1.30168 | −2.936143 | −2.72131 |
| F | W | −3.56630 | −3.38140 | −3.391992 | −3.08975 |
| F | Y | −3.43917 | −2.77317 | −3.509726 | −3.42549 |
| F | A | −2.01230 | −1.83994 | −2.26711 | −2.15333 |
| F | G | −2.01230 | −1.56865 | −2.26711 | −2.15333 |
| F | T | −1.95279 | −1.79604 | −2.047144 | −1.91305 |
| F | S | −1.84605 | −1.66355 | −1.522229 | −1.53233 |
| F | N | −2.03255 | −2.40241 | −2.218329 | −2.27842 |
| F | Q | −1.79398 | −2.03858 | −2.433432 | −2.59128 |
| F | D | −0.99498 | −1.69530 | −0.94471 | −0.75487 |
| F | E | −1.12033 | −1.09783 | −0.93113 | −0.57167 |
| F | H | −2.37259 | −2.05199 | −2.575089 | −2.45269 |
| F | R | −4.50374 | −3.03696 | −5.438522 | −3.98459 |
| F | K | −2.08061 | −1.30816 | −2.257755 | −2.10079 |
| F | P | −2.29473 | −1.97895 |  |  |
| I | I | −4.01318 | −1.46660 | −3.52907 | −3.05002 |
| I | L | −3.76265 | −1.43020 | −3.635822 | −3.05399 |
| I | V | −3.96035 | −1.43962 | −3.549799 | −3.02217 |
| I | W | −2.94878 | −2.10208 | −2.869622 | −2.56945 |
| I | Y | −3.37080 | −2.25773 | −3.311373 | −3.03863 |
| I | A | −2.99546 | −1.48696 | −2.680603 | −2.58416 |
| I | G | −2.99546 | −1.38644 | −2.680603 | −2.58416 |
| I | T | −2.99553 | −1.56220 | −2.508868 | −2.39389 |
| I | S | −2.76449 | −1.66610 | −2.117991 | −1.96492 |
| I | N | −2.52536 | −2.55811 | −2.366421 | −2.44933 |
| I | Q | −3.00896 | −2.01845 | −2.837965 | −2.23577 |
| I | D | −1.01717 | −1.73345 | −0.869899 | −1.58885 |
| I | E | −1.40244 | −1.59699 | −1.15502 | −1.31885 |
| I | H | −2.97079 | −1.62433 | −1.857744 | −2.14395 |
| I | R | −3.89904 | −1.94227 | −3.711743 | −4.06611 |
| I | K | −2.13992 | −1.58615 | −2.100267 | −1.994 |
| I | P | −3.42980 | −1.52216 |  |  |
| L | L | −3.82304 | −0.93259 | −3.797816 | −3.41904 |
| L | V | −3.86267 | −1.63149 | −3.413925 | −2.94842 |
| L | W | −3.02414 | −1.88308 | −2.551283 | −2.67175 |
| L | Y | −3.15649 | −1.93806 | −3.33328 | −2.76316 |
| L | A | −2.83483 | −1.54687 | −3.105816 | −2.7695 |
| L | G | −2.83483 | −1.84977 | −3.105816 | −2.7695 |
| L | T | −2.91129 | −2.23461 | −2.62828 | −2.33968 |
| L | S | −2.48819 | −2.40301 | −2.507219 | −2.30776 |
| L | N | −2.77837 | −2.27150 | −2.857905 | −2.69932 |
| L | Q | −3.07365 | −2.07635 | −3.039278 | −2.80626 |
| L | D | −0.92955 | −1.98325 | −0.599739 | −1.82729 |
| L | E | −1.67601 | −1.06194 | −1.110057 | −2.6239 |
| L | H | −3.17764 | −1.63835 | −2.241073 | −2.5665 |
| L | R | −3.66126 | −2.18864 | −3.291334 | −3.95931 |
| L | K | −2.07002 | −1.67308 | −2.22999 | −2.1836 |
| L | P | −3.35522 | −1.63923 |  |  |
| V | V | −3.79238 | −2.26125 | −3.535987 | −3.36649 |
| V | W | −2.73813 | −1.80166 | −2.702126 | −2.42766 |
| V | Y | −2.96476 | −1.73793 | −2.93205 | −2.65881 |
| V | A | −3.11309 | −1.53896 | −2.876268 | −2.87604 |
| V | G | −3.11309 | −1.38517 | −2.876268 | −2.87604 |
| V | T | −2.86107 | −1.78412 | −2.591535 | −2.40214 |
| V | S | −2.68595 | −2.46446 | −2.169688 | −2.31589 |
| V | N | −2.72058 | −2.57377 | −2.659178 | −2.73813 |
| V | Q | −2.88787 | −1.95542 | −2.707297 | −2.38059 |
| V | D | −1.03742 | −1.70581 | −0.473288 | −0.81049 |
| V | E | −1.33513 | −1.56714 | −1.359756 | −1.28176 |
| V | H | −2.62850 | −2.32664 | −1.963301 | −2.57758 |
| V | R | −3.13778 | −1.82820 | −3.444016 | −4.02032 |
| V | K | −2.37129 | −1.54127 | −2.006928 | −2.25351 |
| V | P | −3.15348 | −0.98615 |  |  |
| W | W | −4.39703 | −3.99217 | −3.693555 | −3.52389 |
| W | Y | −3.58668 | −3.25044 | −3.551122 | −3.34811 |
| W | A | −1.47902 | −1.22216 | −1.959697 | −1.63383 |
| W | G | −1.47902 | −1.64207 | −1.959697 | −1.63383 |
| W | T | −2.10644 | −2.18168 | −1.266968 | −1.60441 |
| W | S | −1.55757 | −2.16924 | −1.493567 | −1.14983 |
| W | N | −2.34506 | −2.34652 | −2.351505 | −2.02956 |
| W | Q | −3.05325 | −3.04111 | −2.51261 | −2.11853 |
| W | D | −1.02100 | −1.37952 | −1.147489 | −1.53821 |
| W | E | −1.02386 | −1.30757 | −1.306437 | −1.56034 |
| W | H | −2.93602 | −2.83422 | −2.616986 | −2.80999 |
| W | R | −7.09368 | −6.16511 | −7.165475 | −5.0276 |
| W | K | −4.77708 | −3.43548 | −3.236233 | −3.79971 |
| W | P | −3.08244 | −2.15874 |  |  |
| Y | Y | −3.63958 | −3.20608 | −3.891154 | −3.3876 |
| Y | A | −1.74571 | −1.60138 | −2.266947 | −2.04908 |
| Y | G | −1.74571 | −1.26081 | −2.266947 | −2.04908 |
| Y | T | −2.17124 | −1.85248 | −2.06998 | −2.06972 |
| Y | S | −1.82703 | −1.77031 | −1.65838 | −1.66973 |
| Y | N | −2.51450 | −1.88370 | −2.909798 | −2.5164 |
| Y | Q | −2.80451 | −1.91097 | −2.659253 | −2.70957 |
| Y | D | −1.05010 | −1.67571 | −0.855842 | −1.57189 |
| Y | E | −1.26683 | −1.53305 | −1.062287 | −1.7597 |
| Y | H | −3.04387 | −2.15948 | −2.66469 | −2.99429 |
| Y | R | −5.72831 | −4.74134 | −6.402685 | −4.65287 |
| Y | K | −3.47647 | −1.54497 | −2.989831 | −3.02447 |
| Y | P | −2.99753 | −2.07329 |  |  |
| A | A | −3.01226 | −1.29831 | −3.103191 | −2.74856 |
| A | G | −3.01226 | −1.77891 | −3.103191 | −2.74856 |
| A | T | −2.39220 | −2.00108 | −2.41223 | −2.33963 |
| A | S | −2.09699 | −2.20592 | −2.176326 | −2.04148 |
| A | N | −2.06177 | −2.63429 | −1.875014 | −2.00766 |
| A | Q | −2.00413 | −1.70793 | −2.079929 | −1.97062 |
| A | D | −0.50172 | −2.44411 | −0.788571 | −1.00016 |
| A | E | −0.84664 | −1.35144 | −1.132324 | −1.60551 |
| A | H | −1.94311 | −1.93409 | −1.506489 | −1.71573 |
| A | R | −1.43226 | −1.23631 | −1.862098 | −2.24881 |
| A | K | −1.70043 | −1.48560 | −1.543294 | −1.71495 |
| A | P | −2.80274 | −1.78663 |  |  |
| G | G | −3.01226 | −1.87863 | −3.103191 | −2.74856 |
| G | T | −2.39220 | −1.93278 | −2.41223 | −2.33963 |
| G | S | −2.09699 | −2.14923 | −2.176326 | −2.04148 |
| G | N | −2.06177 | −2.92082 | −1.875014 | −2.00766 |
| G | Q | −2.00413 | −1.71393 | −2.079929 | −1.97062 |
| G | D | −0.50172 | −2.52797 | −0.788571 | −1.00016 |
| G | E | −0.84664 | −0.89530 | −1.132324 | −1.60551 |
| G | H | −1.94311 | −1.80133 | −1.506489 | −1.71573 |
| G | R | −1.43226 | −1.75894 | −1.862098 | −2.24881 |
| G | K | −1.70043 | −1.60783 | −1.543294 | −1.71495 |
| G | P | −2.80274 | −1.42944 |  |  |
| T | T | −2.17639 | −2.27205 | −1.503483 | −1.53485 |
| T | S | −2.15427 | −2.44856 | −1.236477 | −1.58958 |
| T | N | −2.02294 | −3.12074 | −1.612397 | −1.85041 |
| T | Q | −2.33920 | −2.30994 | −1.918683 | −2.1228 |
| T | D | −0.63305 | −3.37199 | −0.979696 | −0.99971 |
| T | E | −1.13169 | −2.30207 | −0.741976 | −1.30149 |
| T | H | −1.99020 | −1.99645 | −1.434797 | −1.91254 |
| T | R | −1.89405 | −2.12535 | −1.873744 | −2.07694 |
| T | K | −1.59374 | −2.00369 | −1.298625 | −1.24358 |
| T | P | −2.45346 | −1.77473 |  |  |
| S | S | −1.80919 | −2.71935 | −1.480965 | −1.48758 |
| S | N | −1.97778 | −2.88679 | −1.610935 | −1.52195 |
| S | Q | −2.37238 | −2.31760 | −1.449491 | −1.63632 |
| S | D | −0.57952 | −3.35909 | −0.944666 | −0.35543 |
| S | E | −1.32432 | −2.12226 | −1.092381 | −1.20436 |
| S | H | −2.04149 | −2.21046 | −1.172208 | −1.49837 |
| S | R | −2.07649 | −2.59047 | −1.257122 | −1.52234 |
| S | K | −1.48734 | −1.96942 | −1.543335 | −1.23687 |
| S | P | −2.14859 | −1.92283 |  |  |
| N | N | −2.19598 | −3.68717 | −1.860401 | −2.24805 |
| N | Q | −2.28060 | −2.67566 | −1.887453 | −2.58722 |
| N | D | −1.04591 | −3.64227 | −1.163453 | −0.82437 |
| N | E | −1.29822 | −2.36536 | −1.195822 | −0.9502 |
| N | H | −1.87940 | −2.59329 | −1.607374 | −1.968 |
| N | R | −1.92194 | −3.00288 | −2.028265 | −2.43188 |
| N | K | −1.33357 | −2.47627 | −1.127236 | −1.84962 |
| N | P | −2.25369 | −2.60250 |  |  |
| Q | Q | −2.33722 | −2.15872 | −1.843439 | −2.33122 |
| Q | D | −1.29546 | −1.98926 | −1.039706 | −1.02828 |
| Q | E | −1.09240 | −2.27565 | −0.813265 | −0.98915 |
| Q | H | −1.96400 | −1.90415 | −1.432575 | −2.11094 |
| Q | R | −2.26601 | −2.06515 | −2.455156 | −2.71656 |
| Q | K | −1.62957 | −1.60899 | −1.191231 | −1.94533 |
| Q | P | −2.79666 | −1.72891 |  |  |
| D | D | −0.61225 | −2.14122 | −0.657565 | −0.5481 |
| D | E | −0.65366 | −1.30591 | −0.452093 | −1.24952 |
| D | H | −1.41218 | −2.01424 | −0.990453 | −2.50768 |
| D | R | −7.79878 | −5.52904 | −9.174487 | −14.8289 |
| D | K | −3.25082 | −5.02955 | −4.134389 | −4.96536 |
| D | P | −1.06597 | −2.36134 |  |  |
| E | E | −0.80181 | −1.29384 | −1.088523 | −1.62034 |
| E | H | −1.61068 | −1.50644 | −1.045448 | −2.65955 |
| E | R | −7.45705 | −4.42694 | −9.041451 | −10.6337 |
| E | K | −2.76222 | −2.61801 | −4.6303 | −5.07152 |
| E | P | −1.63794 | −1.27329 |  |  |
| H | H | −2.83985 | −2.70975 | −1.369693 | −2.58349 |
| H | R | −3.41747 | −3.47470 | −2.341182 | −2.46081 |
| H | K | −1.94223 | −2.32972 | −1.240025 | −1.76755 |
| H | P | −2.57331 | −1.41680 |  |  |
| R | R | −1.39272 | −1.83495 | −4.750505 | −3.46035 |
| R | K | −0.90115 | −1.45082 | −1.720581 | −1.60461 |
| R | P | −2.74562 | −1.45422 |  |  |
| K | K | −0.39426 | −0.59997 | −0.790318 | −0.5868 |
| K | P | −1.88178 | −1.74852 |  |  |
| P | P | −3.02585 | −1.50787 |  |  |

**Table S2.** Correlation coefficient between residue–residue contact energy and PSAS^*^

| Residue–residue contact energy | F | W | Y |
| --- | --- | --- | --- |
| MD-based energy (whole amino acid) | −0.52 | −0.57 | −0.39 |
| MD-based energy (side chain) | −0.45 | −0.38 | −0.40 |
| 3D structure based energy^**^ | −0.18 | −0.32 | 0.17 |

*Correlation coefficients between the relative contact energies (e_ij_ + e_rr_ − e_ir_ − e_jr_) and PSAS (1) were calculated for three amino acids (F, W, and Y).

**3D structure-based energies are referred from the paper by Miyazawa and Jernigan (2).

**References**

1. A. Nomoto, S. Nishinami, K. Shiraki, Solubility parameters of amino acids on liquid-liquid phase separation and aggregation of proteins. *Front. Cell Dev. Biol.* 9, 691052 (2021).

2. S. Miyazawa, R. L. Jernigan, Residue-residue potentials with a favorable contact pair term and an unfavorable high packing density term, for simulation and threading. *J. Mol. Biol.* 256, 623-644 (1996).

**Supplementary Text 1**

The atom type from amber force field (AMBER 99SB force field) and charge of the amino acid and amino acid analog molecules modeled using quantum chemical calculations is described below in Tripos mol2 format.

**Amino acids:**

@<TRIPOS>MOLECULE

ALA

13 12 1 0 0

SMALL

No Charge or Current Charge

@<TRIPOS>ATOM

1 N 3.5400 1.4200 0.0000 N3 1 ALA -0.515277

2 H1 2.6290 0.9400 -0.2710 H 1 ALA 0.313843

3 H2 3.9930 0.9560 0.7680 H 1 ALA 0.313843

4 H3 4.1710 1.4400 -0.7830 H 1 ALA 0.313843

5 CA 2.9590 2.7740 0.3380 CT 1 ALA 0.256852

6 HA 3.0400 2.8960 1.4090 HP 1 ALA 0.029945

7 CB 3.6700 3.9010 -0.3900 CT 1 ALA -0.311260

8 HB1 4.7080 3.9980 -0.0850 HC 1 ALA 0.094268

9 HB2 3.6300 3.7550 -1.4660 HC 1 ALA 0.094268

10 HB3 3.1500 4.8210 -0.1660 HC 1 ALA 0.094268

11 C 1.4410 2.6380 -0.0440 C 1 ALA 0.717019

12 O 1.1510 1.4830 -0.4100 O2 1 ALA -0.700807

13 OXT 0.7900 3.6470 0.0760 O2 1 ALA -0.700807

@<TRIPOS>BOND

1 1 2 1

2 1 3 1

3 1 4 1

4 1 5 1

5 5 6 1

6 5 7 1

7 5 11 1

8 7 8 1

9 7 9 1

10 7 10 1

11 11 12 1

12 11 13 1

@<TRIPOS>SUBSTRUCTURE

1 ALA 1 TEMP 0 **** **** 0 ROOT

@<TRIPOS>MOLECULE

ARG

27 26 1 0 0

SMALL

No Charge or Current Charge

@<TRIPOS>ATOM

1 N 3.5400 1.4200 0.0000 N3 1 ARG -0.611770

2 H1 4.2670 0.9590 0.5260 H 1 ARG 0.374033

3 H2 3.1440 0.7160 -0.6300 H 1 ARG 0.374033

4 H3 3.9580 2.1800 -0.5130 H 1 ARG 0.374033

5 CA 2.3790 1.8130 0.8840 CT 1 ARG 0.192343

6 HA 2.7760 2.0940 1.8480 HP 1 ARG 0.056317

7 CB 1.6380 2.9850 0.2120 CT 1 ARG -0.259857

8 HB1 2.3820 3.7250 -0.0710 HC 1 ARG 0.095967

9 HB2 1.1930 2.6260 -0.7110 HC 1 ARG 0.095967

10 CG 0.5800 3.7140 1.0810 CT 1 ARG 0.054325

11 HG1 0.7530 3.5210 2.1340 HC 1 ARG 0.038065

12 HG2 0.7130 4.7790 0.9480 HC 1 ARG 0.038065

13 CD -0.8890 3.4180 0.7540 CT 1 ARG 0.117976

14 HD1 -1.5120 4.1300 1.2830 H1 1 ARG 0.043674

15 HD2 -1.0670 3.5420 -0.3080 H1 1 ARG 0.043674

16 NE -1.2280 2.0600 1.1580 N2 1 ARG -0.576932

17 HE -0.5740 1.6110 1.7780 H 1 ARG 0.342767

18 CZ -2.0490 1.2200 0.5600 CA 1 ARG 0.941863

19 NH2 -2.9940 1.6550 -0.2720 N2 1 ARG -0.959313

20 HH3 -3.5980 1.0140 -0.7380 H 1 ARG 0.459605

21 HH4 -3.2750 2.6100 -0.2640 H 1 ARG 0.459605

22 NH1 -1.9110 -0.0730 0.8010 N2 1 ARG -0.959313

23 HH1 -1.0650 -0.3950 1.2390 H 1 ARG 0.459605

24 HH2 -2.5550 -0.7450 0.4490 H 1 ARG 0.459605

25 C 1.5180 0.5180 0.9680 C 1 ARG 0.770041

26 O 1.6450 -0.2250 0.0050 O2 1 ARG -0.712189

27 OXT 0.7780 0.4370 1.9500 O2 1 ARG -0.712189

@<TRIPOS>BOND

1 2 1 1

2 3 1 1

3 4 1 1

4 5 1 1

5 6 5 1

6 7 5 1

7 8 7 1

8 9 7 1

9 10 7 1

10 11 10 1

11 12 10 1

12 13 10 1

13 14 13 1

14 15 13 1

15 16 13 1

16 17 16 1

17 18 16 2

18 19 18 1

19 20 19 1

20 21 19 1

21 22 18 1

22 23 22 1

23 24 22 1

24 25 5 1

25 26 25 1

26 27 25 1

@<TRIPOS>SUBSTRUCTURE

1 ARG 1 TEMP 0 **** **** 0 ROOT

@<TRIPOS>MOLECULE

ASN

17 16 1 0 0

SMALL

No Charge or Current Charge

@<TRIPOS>ATOM

1 N 3.5400 1.4200 0.0000 N3 1 ASN -0.500242

2 H1 3.3990 2.1480 0.6780 H 1 ASN 0.328123

3 H2 4.4590 0.9430 0.1030 H 1 ASN 0.328123

4 H3 3.5060 1.8270 -0.9210 H 1 ASN 0.328123

5 CA 2.6020 0.2450 0.1370 CT 1 ASN 0.176960

6 HA 2.3140 0.2220 1.1800 HP 1 ASN 0.055040

7 CB 1.3760 0.4050 -0.7690 CT 1 ASN -0.225519

8 HB1 1.1410 1.4520 -0.9140 HC 1 ASN 0.078633

9 HB2 1.5800 -0.0410 -1.7370 HC 1 ASN 0.078633

10 CG 0.0960 -0.2060 -0.1970 C 1 ASN 0.817283

11 ND1 0.1640 -1.5090 0.1030 N 1 ASN -1.079277

12 HD1 1.0050 -2.0320 -0.0550 H 1 ASN 0.465077

13 HD2 -0.6520 -1.9510 0.4610 H 1 ASN 0.465077

14 OD2 -0.8810 0.4780 -0.0430 O 1 ASN -0.639670

15 C 3.5170 -1.0030 -0.1490 C 1 ASN 0.728739

16 O 2.9260 -2.0420 -0.3720 O2 1 ASN -0.702552

17 OXT 4.7190 -0.7320 -0.0950 O2 1 ASN -0.702552

@<TRIPOS>BOND

1 2 1 1

2 3 1 1

3 4 1 1

4 5 1 1

5 6 5 1

6 7 5 1

7 8 7 1

8 9 7 1

9 10 7 1

10 11 10 1

11 12 11 1

12 13 11 1

13 14 10 2

14 15 5 1

15 16 15 1

16 17 15 1

@<TRIPOS>SUBSTRUCTURE

1 ASN 1 TEMP 0 **** **** 0 ROOT

@<TRIPOS>MOLECULE

ASP

15 14 1 0 0

SMALL

No Charge or Current Charge

@<TRIPOS>ATOM

1 N 3.5400 1.4200 0.0000 N3 1 ASP -0.387282

2 H1 3.2110 1.9610 -0.7780 H 1 ASP 0.268918

3 H2 2.9400 1.6340 0.7900 H 1 ASP 0.268918

4 H3 4.5800 1.5400 0.1480 H 1 ASP 0.268918

5 CA 3.3410 -0.0480 -0.2210 CT 1 ASP 0.095915

6 HA 3.4560 -0.2410 -1.2790 HP 1 ASP 0.034641

7 CB 4.4170 -0.7980 0.5730 CT 1 ASP -0.153166

8 HB1 4.2060 -0.6810 1.6350 HC 1 ASP 0.052982

9 HB2 4.3350 -1.8510 0.3480 HC 1 ASP 0.052982

10 CG 5.8730 -0.3200 0.3320 C 1 ASP 0.801659

11 OG1 6.0150 0.9280 0.1940 O2 1 ASP -0.763508

12 OG2 6.7430 -1.1670 0.3350 O2 1 ASP -0.763508

13 C 1.8940 -0.3940 0.2390 C 1 ASP 0.741770

14 O 1.4740 -1.4740 -0.1400 O2 1 ASP -0.759619

15 OXT 1.3750 0.4810 0.9490 O2 1 ASP -0.759619

@<TRIPOS>BOND

1 2 1 1

2 3 1 1

3 4 1 1

4 5 1 1

5 6 5 1

6 7 5 1

7 8 7 1

8 9 7 1

9 10 7 1

10 11 10 1

11 12 10 1

12 13 5 1

13 14 13 1

14 15 13 1

@<TRIPOS>SUBSTRUCTURE

1 ASP 1 TEMP 0 **** **** 0 ROOT

@<TRIPOS>MOLECULE

CYS

14 13 1 0 0

SMALL

No Charge or Current Charge

@<TRIPOS>ATOM

1 N 3.5400 1.4200 0.0000 N3 1 CYS -0.349791

2 H1 3.4740 2.1680 -0.6670 H 1 CYS 0.262307

3 H2 2.7080 0.7690 -0.0180 H 1 CYS 0.262307

4 H3 3.6140 1.8120 0.9240 H 1 CYS 0.262307

5 CA 4.6170 0.4000 -0.2900 CT 1 CYS 0.345931

6 HA 4.8200 0.4630 -1.3490 HP 1 CYS -0.015686

7 CB 5.8790 0.6670 0.5200 CT 1 CYS -0.271377

8 HB1 5.7000 0.4410 1.5640 H1 1 CYS 0.118029

9 HB2 6.1650 1.7090 0.4390 H1 1 CYS 0.118029

10 SG 7.2810 -0.3420 -0.0750 SH 1 CYS -0.222499

11 HG 8.1540 0.6560 -0.1270 HS 1 CYS 0.152092

12 C 3.9090 -0.9700 0.0210 C 1 CYS 0.683376

13 O 4.6490 -1.9110 0.1690 O2 1 CYS -0.672512

14 OXT 2.6710 -0.8500 0.0660 O2 1 CYS -0.672512

@<TRIPOS>BOND

1 2 1 1

2 3 1 1

3 4 1 1

4 5 1 1

5 6 5 1

6 7 5 1

7 8 7 1

8 9 7 1

9 10 7 1

10 11 10 1

11 12 5 1

12 13 12 1

13 14 12 1

@<TRIPOS>SUBSTRUCTURE

1 CYS 1 TEMP 0 **** **** 0 ROOT

@<TRIPOS>MOLECULE

GLN

20 19 1 0 0

SMALL

No Charge or Current Charge

@<TRIPOS>ATOM

1 N 3.5400 1.4200 0.0000 N3 1 GLN -0.535580

2 H1 3.1320 1.8840 -0.7920 H 1 GLN 0.326737

3 H2 2.8130 0.8570 0.5230 H 1 GLN 0.326737

4 H3 3.9590 2.1130 0.5980 H 1 GLN 0.326737

5 CA 4.4780 0.2900 -0.3640 CT 1 GLN 0.125473

6 HA 4.4970 0.2200 -1.4430 HP 1 GLN 0.057638

7 CB 5.8730 0.5780 0.1880 CT 1 GLN 0.004715

8 HB2 5.8320 0.5930 1.2740 HC 1 GLN 0.036964

9 HB3 6.1980 1.5640 -0.1280 HC 1 GLN 0.036964

10 CG 6.9150 -0.4410 -0.2650 CT 1 GLN -0.426915

11 HG2 6.6450 -1.4300 0.0760 HC 1 GLN 0.142599

12 HG3 6.9470 -0.4890 -1.3510 HC 1 GLN 0.142599

13 CD 8.3040 -0.0540 0.2090 C 1 GLN 0.968959

14 OE1 8.5870 1.0600 0.5580 O 1 GLN -0.655132

15 NE2 9.2160 -1.0530 0.1660 N 1 GLN -1.121305

16 HE21 9.0170 -1.9240 -0.2670 H 1 GLN 0.452525

17 HE22 9.9970 -0.9990 0.7810 H 1 GLN 0.452525

18 C 3.7780 -0.9860 0.2280 C 1 GLN 0.774925

19 O 4.3510 -2.0330 0.0260 O2 1 GLN -0.718583

20 OXT 2.7200 -0.7160 0.8220 O2 1 GLN -0.718583

@<TRIPOS>BOND

1 2 1 1

2 3 1 1

3 4 1 1

4 5 1 1

5 6 5 1

6 7 5 1

7 8 7 1

8 9 7 1

9 10 7 1

10 11 10 1

11 12 10 1

12 13 10 1

13 14 13 2

14 15 13 1

15 16 15 1

16 17 15 1

17 18 5 1

18 19 18 1

19 20 18 1

@<TRIPOS>SUBSTRUCTURE

1 GLN 1 TEMP 0 **** **** 0 ROOT

@<TRIPOS>MOLECULE

GLU

18 17 1 0 0

SMALL

No Charge or Current Charge

@<TRIPOS>ATOM

1 N 3.5400 1.4200 0.0000 N3 1 GLU -0.535768

2 H1 3.0720 1.7760 -0.8130 H 1 GLU 0.312497

3 H2 2.8280 1.0550 0.6800 H 1 GLU 0.312497

4 H3 4.1290 2.1380 0.3860 H 1 GLU 0.312497

5 CA 4.2930 0.1190 -0.2560 CT 1 GLU 0.073854

6 HA 4.4060 0.0100 -1.3250 HP 1 GLU 0.055971

7 CB 5.6560 0.2280 0.4300 CT 1 GLU 0.046167

8 HB1 6.0880 1.1970 0.2100 HC 1 GLU 0.019193

9 HB2 5.5110 0.1840 1.5080 HC 1 GLU 0.019193

10 CG 6.7040 -0.7920 -0.0040 CT 1 GLU -0.285108

11 HG1 6.5300 -1.7640 0.4360 HC 1 GLU 0.067773

12 HG2 6.6500 -0.9320 -1.0830 HC 1 GLU 0.067773

13 CD 8.1560 -0.3090 0.3140 C 1 GLU 0.875986

14 OD1 8.9970 -1.2030 0.3580 O2 1 GLU -0.822582

15 OD2 8.2970 0.9120 0.4480 O2 1 GLU -0.822582

16 C 3.3190 -0.9680 0.3040 C 1 GLU 0.799141

17 O 3.5520 -2.1110 -0.0130 O2 1 GLU -0.748251

18 OXT 2.4080 -0.4820 1.0130 O2 1 GLU -0.748251

@<TRIPOS>BOND

1 2 1 1

2 3 1 1

3 4 1 1

4 5 1 1

5 6 5 1

6 7 5 1

7 8 7 1

8 9 7 1

9 10 7 1

10 11 10 1

11 12 10 1

12 13 10 1

13 14 13 1

14 15 13 1

15 16 5 1

16 17 16 1

17 18 16 1

@<TRIPOS>SUBSTRUCTURE

1 GLU 1 TEMP 0 **** **** 0 ROOT

@<TRIPOS>MOLECULE

GLY

10 9 1 0 0

SMALL

No Charge or Current Charge

@<TRIPOS>ATOM

1 N1 3.5400 1.4200 0.0000 N3 1 GLY -0.487586

2 H1 4.1050 2.3340 0.0030 H 1 GLY 0.324087

3 H2 2.9560 1.3500 0.8140 H 1 GLY 0.324087

4 H3 2.9640 1.3530 -0.8200 H 1 GLY 0.324087

5 C1 4.7180 0.4880 0.0030 CT 1 GLY -0.042396

6 H4 4.6940 -0.1310 0.8860 HP 1 GLY 0.083854

7 H5 4.6950 -0.1360 -0.8760 HP 1 GLY 0.083854

8 C2 5.9700 1.4340 0.0010 C 1 GLY 0.851416

9 O2 5.6310 2.6350 0.0020 O2 1 GLY -0.730702

10 O1 7.0380 0.8800 0.0000 O2 1 GLY -0.730702

@<TRIPOS>BOND

1 1 2 1

2 1 3 1

3 1 4 1

4 1 5 1

5 5 6 1

6 5 7 1

7 5 8 1

8 8 9 1

9 8 10 1

@<TRIPOS>SUBSTRUCTURE

1 GLY 1 TEMP 0 **** **** 0 ROOT

@<TRIPOS>MOLECULE

HIS

20 20 1 0 0

SMALL

No Charge or Current Charge

@<TRIPOS>ATOM

1 N 3.5400 1.4200 0.0000 N3 1 HIS -0.442330

2 H1 3.6150 0.6670 0.6610 H 1 HIS 0.306917

3 H2 2.7160 2.0380 0.1700 H 1 HIS 0.306917

4 H3 3.4540 1.0310 -0.9240 H 1 HIS 0.306917

5 CA 4.6510 2.4440 0.0820 CT 1 HIS 0.097643

6 HA 5.0120 2.4050 1.1010 HP 1 HIS 0.059248

7 CB 5.7780 2.1230 -0.9020 CT 1 HIS -0.045261

8 HB1 5.5010 2.4900 -1.8850 HC 1 HIS 0.035900

9 HB2 5.9050 1.0460 -0.9740 HC 1 HIS 0.035900

10 CG 7.1170 2.6660 -0.4890 CC 1 HIS 0.338279

11 ND 8.1500 1.8020 -0.2090 NB 1 HIS -0.629989

12 CE 9.1540 2.5500 0.1010 CR 1 HIS 0.264974

13 HE1 10.1290 2.2050 0.3750 H5 1 HIS 0.103696

14 NE 8.8400 3.8590 0.0450 NA 1 HIS -0.324103

15 HE2 9.4450 4.6220 0.2460 H 1 HIS 0.344369

16 CD 7.5260 3.9460 -0.3340 CW 1 HIS -0.355934

17 HD 7.0010 4.8680 -0.4400 H4 1 HIS 0.280863

18 C 3.9050 3.8100 -0.1380 C 1 HIS 0.773130

19 O 4.6070 4.7530 -0.4280 O2 1 HIS -0.728568

20 OXT 2.6790 3.7080 0.0310 O2 1 HIS -0.728568

@<TRIPOS>BOND

1 2 1 1

2 3 1 1

3 4 1 1

4 5 1 1

5 6 5 1

6 7 5 1

7 8 7 1

8 9 7 1

9 10 7 1

10 11 10 2

11 12 11 1

12 13 12 1

13 14 12 1

14 15 14 1

15 16 14 3

16 17 16 1

17 18 5 1

18 19 18 1

19 20 18 1

20 12 10 1

@<TRIPOS>SUBSTRUCTURE

1 HIS 1 TEMP 0 **** **** 0 ROOT

@<TRIPOS>MOLECULE

ILE

22 21 1 0 0

SMALL

No Charge or Current Charge

@<TRIPOS>ATOM

1 N1 3.5400 1.4200 0.0000 N3 1 ILE -0.656942

2 H1 3.3780 1.9920 -0.8110 H 1 ILE 0.358980

3 H2 2.6490 1.3430 0.5790 H 1 ILE 0.358980

4 H3 4.2940 1.8180 0.5290 H 1 ILE 0.358980

5 CA 3.6980 -0.0530 -0.3170 CT 1 ILE 0.020991

6 HA 3.7600 -0.1600 -1.3910 HP 1 ILE 0.085867

7 CB 4.9730 -0.6020 0.3510 CT 1 ILE 0.088855

8 HB 4.9110 -0.3530 1.4120 HC 1 ILE 0.034745

9 CG1 5.0540 -2.1290 0.2290 CT 1 ILE -0.288744

10 HG11 4.1850 -2.6060 0.6490 HC 1 ILE 0.096008

11 HG12 5.1190 -2.4280 -0.8130 HC 1 ILE 0.096008

12 HG13 5.9350 -2.4980 0.7420 HC 1 ILE 0.096008

13 CG2 6.2340 0.0670 -0.2390 CT 1 ILE -0.042014

14 HG21 6.3440 -0.2530 -1.2730 HC 1 ILE 0.021296

15 HG22 6.1130 1.1480 -0.2770 HC 1 ILE 0.021296

16 CD 7.5260 -0.2210 0.5270 CT 1 ILE -0.189910

17 HD1 5.2190 1.4250 -0.8290 HC 1 ILE 0.054389

18 HD2 6.0400 1.5850 0.7130 HC 1 ILE 0.054389

19 HD3 6.9620 1.6060 -0.7730 HC 1 ILE 0.054389

20 C 2.3330 -0.6620 0.1880 C 1 ILE 0.839960

21 O 2.0950 -1.7920 -0.1610 O2 1 ILE -0.731765

22 OXT 1.6840 0.1500 0.8770 O2 1 ILE -0.731765

@<TRIPOS>BOND

1 2 1 1

2 3 1 1

3 4 1 1

4 5 1 1

5 6 5 1

6 7 5 1

7 8 7 1

8 9 7 1

9 10 9 1

10 11 9 1

11 12 9 1

12 13 7 1

13 14 13 1

14 15 13 1

15 16 13 1

16 17 15 1

17 18 15 1

18 19 15 1

19 20 5 1

20 21 20 1

21 22 20 1

@<TRIPOS>SUBSTRUCTURE

1 ILE 1 TEMP 0 **** **** 0 ROOT

@<TRIPOS>MOLECULE

LEU

22 21 1 0 0

SMALL

No Charge or Current Charge

@<TRIPOS>ATOM

1 N 3.5400 1.4200 0.0000 N3 1 LEU -0.503795

2 H1 3.8840 0.9960 0.8420 H 1 LEU 0.306971

3 H2 2.4810 1.3560 -0.0730 H 1 LEU 0.306971

4 H3 3.9690 0.9660 -0.7870 H 1 LEU 0.306971

5 CA 3.6550 2.9280 -0.0430 CT 1 LEU 0.260941

6 HA 3.9270 3.2490 0.9520 HP 1 LEU 0.024546

7 CB 4.6440 3.4310 -1.0870 CT 1 LEU -0.368385

8 HB1 4.4490 2.9150 -2.0270 HC 1 LEU 0.105942

9 HB2 4.3700 4.4640 -1.2550 HC 1 LEU 0.105942

10 CG 6.1480 3.3590 -0.7640 CT 1 LEU 0.444506

11 HG 6.6480 3.7690 -1.6400 HC 1 LEU -0.033365

12 CD1 6.5350 4.2400 0.4280 CT 1 LEU -0.416375

13 HD11 6.1760 5.2560 0.2980 HC 1 LEU 0.093662

14 HD12 6.1280 3.8630 1.3620 HC 1 LEU 0.093662

15 HD13 7.6140 4.2800 0.5410 HC 1 LEU 0.093662

16 CD2 6.6800 1.9310 -0.5810 CT 1 LEU -0.416375

17 HD21 6.3080 1.4730 0.3320 HC 1 LEU 0.093662

18 HD22 6.4140 1.2920 -1.4190 HC 1 LEU 0.093662

19 HD23 7.7620 1.9310 -0.5090 HC 1 LEU 0.093662

20 C 2.1770 3.4030 -0.3130 C 1 LEU 0.714961

21 O 2.0380 4.5910 -0.4720 O2 1 LEU -0.700714

22 OXT 1.3680 2.4550 -0.3050 O2 1 LEU -0.700714

@<TRIPOS>BOND

1 2 1 1

2 3 1 1

3 4 1 1

4 5 1 1

5 6 5 1

6 7 5 1

7 8 7 1

8 9 7 1

9 10 7 1

10 11 10 1

11 12 10 1

12 13 12 1

13 14 12 1

14 15 12 1

15 16 10 1

16 17 16 1

17 18 16 1

18 19 16 1

19 20 5 1

20 21 20 1

21 22 20 1

@<TRIPOS>SUBSTRUCTURE

1 LEU 1 TEMP 0 **** **** 0 ROOT

@<TRIPOS>MOLECULE

LYS

25 24 1 0 0

SMALL

No Charge or Current Charge

@<TRIPOS>ATOM

1 N 3.5400 1.4200 0.0000 N3 1 LYS -0.606886

2 H1 2.9020 0.7710 -0.4370 H 1 LYS 0.371535

3 H2 3.9060 0.9600 0.8330 H 1 LYS 0.371535

4 H3 3.0210 2.2490 0.2480 H 1 LYS 0.371535

5 CA 4.7500 1.6830 -0.8630 CT 1 LYS 0.250913

6 HA 4.4320 1.6990 -1.8940 H1 1 LYS 0.046956

7 CB 5.3840 3.0250 -0.4420 CT 1 LYS -0.221549

8 HB1 4.8210 3.8280 -0.9070 HC 1 LYS 0.082035

9 HB2 5.2750 3.1460 0.6330 HC 1 LYS 0.082035

10 CG 6.8770 3.1630 -0.8120 CT 1 LYS -0.004370

11 HG1 7.0550 2.6770 -1.7620 HC 1 LYS 0.050694

12 HG2 7.0860 4.2150 -0.9590 HC 1 LYS 0.050694

13 CD 7.8380 2.6220 0.2800 CT 1 LYS -0.057954

14 HD1 8.1870 3.4570 0.8750 HC 1 LYS 0.053687

15 HD2 7.3140 1.9710 0.9710 HC 1 LYS 0.053687

16 CE 9.0840 1.8840 -0.2320 CT 1 LYS 0.035789

17 HE1 9.3820 2.2200 -1.2150 HP 1 LYS 0.075606

18 HE2 9.9190 2.0160 0.4400 HP 1 LYS 0.075606

19 NZ 8.8490 0.4050 -0.3360 N3 1 LYS -0.263625

20 HZ1 9.6400 -0.0710 -0.7430 H 1 LYS 0.287970

21 HZ2 8.6810 -0.0040 0.5720 H 1 LYS 0.287970

22 HZ3 7.9930 0.1950 -0.9020 H 1 LYS 0.287970

23 C 5.7050 0.5060 -0.5970 C 1 LYS 0.722530

24 O 6.5030 0.2790 -1.5130 O2 1 LYS -0.702181

25 OXT 5.6280 0.0140 0.5210 O2 1 LYS -0.702181

@<TRIPOS>BOND

1 2 1 1

2 3 1 1

3 4 1 1

4 5 1 1

5 6 5 1

6 7 5 1

7 8 7 1

8 9 7 1

9 10 7 1

10 11 10 1

11 12 10 1

12 13 10 1

13 14 13 1

14 15 13 1

15 16 13 1

16 17 16 1

17 18 16 1

18 19 16 1

19 20 19 1

20 21 19 1

21 22 19 1

22 23 5 1

23 24 23 1

24 25 23 1

@<TRIPOS>SUBSTRUCTURE

1 LYS 1 TEMP 0 **** **** 0 ROOT

@<TRIPOS>MOLECULE

MET

20 19 1 0 0

SMALL

No Charge or Current Charge

@<TRIPOS>ATOM

1 N 3.5400 1.4200 0.0000 N3 1 MET -0.464024

2 H1 3.9610 0.9840 -0.8010 H 1 MET 0.312025

3 H2 4.2290 2.0680 0.4880 H 1 MET 0.312025

4 H3 3.2140 0.7020 0.6250 H 1 MET 0.312025

5 CA 2.4910 2.4550 -0.3420 CT 1 MET 0.023562

6 HA 2.4230 2.5050 -1.4200 HP 1 MET 0.078581

7 CB 1.1460 2.0580 0.2670 CT 1 MET -0.008332

8 HB1 1.2240 2.0740 1.3510 HC 1 MET 0.074006

9 HB2 0.9140 1.0360 -0.0260 HC 1 MET 0.074006

10 CG 0.0020 2.9710 -0.1720 CT 1 MET -0.395018

11 HG1 0.1750 3.9740 0.1820 H1 1 MET 0.206604

12 HG2 -0.0570 3.0010 -1.2540 H1 1 MET 0.206604

13 SD -1.5650 2.3320 0.4930 S 1 MET -0.254237

14 CE -2.6780 3.6740 0.0100 CT 1 MET -0.135428

15 HE1 -2.3760 4.6080 0.4650 H1 1 MET 0.096729

16 HE2 -2.7090 3.7870 -1.0660 H1 1 MET 0.096729

17 HE3 -3.6680 3.4160 0.3630 H1 1 MET 0.096729

18 C 3.1020 3.7970 0.2050 C 1 MET 0.835144

19 O 2.4390 4.7880 0.0090 O2 1 MET -0.733865

20 OXT 4.2000 3.6210 0.7640 O2 1 MET -0.733865

@<TRIPOS>BOND

1 2 1 1

2 3 1 1

3 4 1 1

4 5 1 1

5 6 5 1

6 7 5 1

7 8 7 1

8 9 7 1

9 10 7 1

10 11 10 1

11 12 10 1

12 13 10 1

13 14 13 1

14 15 14 1

15 16 14 1

16 17 14 1

17 18 5 1

18 19 18 1

19 20 18 1

@<TRIPOS>SUBSTRUCTURE

1 MET 1 TEMP 0 **** **** 0 ROOT

@<TRIPOS>MOLECULE

PHE

23 23 1 0 0

SMALL

No Charge or Current Charge

@<TRIPOS>ATOM

1 N -2.4750 1.6130 -0.3290 N3 1 PHE -0.684514

2 H1 -2.2700 2.3160 -1.0170 H 1 PHE 0.357221

3 H2 -2.6100 2.0620 0.5620 H 1 PHE 0.357221

4 H3 -3.3370 1.0580 -0.5480 H 1 PHE 0.357221

5 CA -1.4720 0.4840 -0.2570 CT 1 PHE 0.323339

6 HA -1.0240 0.4310 -1.2400 HP 1 PHE 0.025340

7 CB -0.3990 0.7210 0.8190 CT 1 PHE -0.092398

8 HB2 -0.3730 1.7760 1.0790 HC 1 PHE 0.042934

9 HB3 -0.6830 0.1760 1.7110 HC 1 PHE 0.042934

10 CG 0.9990 0.3120 0.3890 CA 1 PHE 0.043353

11 CD1 1.3210 -1.0270 0.1840 CA 1 PHE -0.201583

12 HD1 0.5630 -1.7750 0.3270 HA 1 PHE 0.166230

13 CE1 2.5990 -1.3830 -0.2100 CA 1 PHE -0.139827

14 HE1 2.8350 -2.4210 -0.3650 HA 1 PHE 0.138293

15 CZ 3.5730 -0.4140 -0.4040 CA 1 PHE -0.122231

16 HZ 4.5650 -0.6970 -0.7080 HA 1 PHE 0.130464

17 CE2 3.2600 0.9180 -0.2010 CA 1 PHE -0.139827

18 HE2 4.0080 1.6780 -0.3450 HA 1 PHE 0.138293

19 CD2 1.9790 1.2760 0.1930 CA 1 PHE -0.201583

20 HD2 1.7500 2.3160 0.3550 HA 1 PHE 0.166230

21 C -2.3780 -0.7890 -0.0620 C 1 PHE 0.655266

22 O -1.8090 -1.7840 0.3240 O2 1 PHE -0.681189

23 OXT -3.5650 -0.5660 -0.3530 O2 1 PHE -0.681189

@<TRIPOS>BOND

1 1 2 1

2 1 3 1

3 1 4 1

4 1 5 1

5 5 6 1

6 5 7 1

7 5 21 1

8 7 8 1

9 7 9 1

10 7 10 1

11 10 11 1

12 10 19 2

13 11 12 1

14 11 13 2

15 13 14 1

16 13 15 1

17 15 16 1

18 15 17 2

19 17 18 1

20 17 19 1

21 19 20 1

22 21 22 1

23 21 23 1

@<TRIPOS>SUBSTRUCTURE

1 PHE 1 TEMP 0 **** **** 0 ROOT

@<TRIPOS>MOLECULE

PRO

17 17 1 0 0

SMALL

No Charge or Current Charge

@<TRIPOS>ATOM

1 N 3.5400 1.4200 0.0000 N3 1 PRO -0.186594

2 H1 2.7880 0.7860 -0.3310 H 1 PRO 0.252506

3 H2 3.9180 1.1050 0.8760 H 1 PRO 0.252506

4 CD 4.6030 1.6250 -1.0220 CT 1 PRO -0.013725

5 HD2 4.1390 1.4630 -1.9850 HP 1 PRO 0.088809

6 HD3 5.3990 0.9080 -0.8830 HP 1 PRO 0.088809

7 CG 4.9950 3.0840 -0.8260 CT 1 PRO 0.012026

8 HG2 5.5120 3.4800 -1.6900 HC 1 PRO 0.014985

9 HG3 5.6610 3.1740 0.0280 HC 1 PRO 0.014985

10 CB 3.6530 3.7800 -0.5520 CT 1 PRO -0.054014

11 HB2 3.1840 4.1080 -1.4700 HC 1 PRO 0.063366

12 HB3 3.7690 4.6520 0.0780 HC 1 PRO 0.063366

13 CA 2.7680 2.7130 0.1150 CT 1 PRO -0.010823

14 HA 2.5870 2.9080 1.1620 HP 1 PRO 0.080611

15 C 1.4030 2.4460 -0.6110 C 1 PRO 0.761073

16 O 1.2600 1.2550 -0.9360 O2 1 PRO -0.713943

17 OXT 0.7110 3.4270 -0.7510 O2 1 PRO -0.713943

@<TRIPOS>BOND

1 2 1 1

2 3 1 1

3 4 1 1

4 5 4 1

5 6 4 1

6 7 4 1

7 8 7 1

8 9 7 1

9 10 7 1

10 11 10 1

11 12 10 1

12 13 1 1

13 14 13 1

14 15 13 1

15 16 15 1

16 17 15 1

17 10 13 1

@<TRIPOS>SUBSTRUCTURE

1 PRO 1 TEMP 0 **** **** 0 ROOT

@<TRIPOS>MOLECULE

SER

14 13 1 0 0

SMALL

No Charge or Current Charge

@<TRIPOS>ATOM

1 N 3.5400 1.4200 0.0000 N3 1 SER -0.490804

2 H1 3.4490 2.1540 -0.6810 H 1 SER 0.324328

3 H2 2.6730 0.8390 0.0810 H 1 SER 0.324328

4 H3 3.7270 1.8360 0.8990 H 1 SER 0.324328

5 CA 4.5320 0.3450 -0.3610 CT 1 SER -0.000534

6 HA 4.6930 0.3990 -1.4280 HP 1 SER 0.098578

7 CB 5.8650 0.5110 0.3700 CT 1 SER 0.223782

8 HB2 5.6980 0.4190 1.4440 H1 1 SER 0.025961

9 HB3 6.2810 1.4960 0.1840 H1 1 SER 0.025961

10 OG 6.7740 -0.4180 -0.0930 OH 1 SER -0.680545

11 HG 6.3520 -1.2750 -0.0390 HO 1 SER 0.459711

12 C 3.7930 -0.9920 -0.0100 C 1 SER 0.781504

13 O 4.5070 -1.9760 0.0250 O2 1 SER -0.708299

14 OXT 2.5820 -0.8330 0.1860 O2 1 SER -0.708299

@<TRIPOS>BOND

1 2 1 1

2 3 1 1

3 4 1 1

4 5 1 1

5 6 5 1

6 7 5 1

7 8 7 1

8 9 7 1

9 10 7 1

10 11 10 1

11 12 5 1

12 13 12 1

13 14 12 1

@<TRIPOS>SUBSTRUCTURE

1 SER 1 TEMP 0 **** **** 0 ROOT

@<TRIPOS>MOLECULE

THR

17 16 1 0 0

SMALL

No Charge or Current Charge

@<TRIPOS>ATOM

1 N 3.5400 1.4200 0.0000 N3 1 THR -0.441066

2 H1 2.5310 1.2280 -0.0970 H 1 THR 0.320024

3 H2 3.9000 1.6680 -0.9110 H 1 THR 0.320024

4 H3 4.0510 0.6340 0.3620 H 1 THR 0.320024

5 CA 3.5330 2.6500 0.8640 CT 1 THR -0.072950

6 HA 3.5450 2.3210 1.8950 HP 1 THR 0.092114

7 CB 4.7160 3.5440 0.5260 CT 1 THR 0.459577

8 HB 4.6040 4.4310 1.1340 H1 1 THR 0.004595

9 CG2 6.0780 2.9160 0.7790 CT 1 THR -0.369473

10 HG21 6.1840 2.6220 1.8190 HC 1 THR 0.096136

11 HG22 6.2400 2.0420 0.1540 HC 1 THR 0.096136

12 HG23 6.8570 3.6280 0.5420 HC 1 THR 0.096136

13 OG1 4.6400 3.8720 -0.8390 OH 1 THR -0.712151

14 HG1 3.9270 4.4970 -0.9400 HO 1 THR 0.448805

15 C 2.1460 3.3170 0.5330 C 1 THR 0.774912

16 O 2.1200 4.5260 0.6480 O2 1 THR -0.716421

17 OXT 1.3020 2.4900 0.1740 O2 1 THR -0.716421

@<TRIPOS>BOND

1 2 1 1

2 3 1 1

3 4 1 1

4 5 1 1

5 6 5 1

6 7 5 1

7 8 7 1

8 9 7 1

9 10 9 1

10 11 9 1

11 12 9 1

12 13 7 1

13 14 13 1

14 15 5 1

15 16 15 1

16 17 15 1

@<TRIPOS>SUBSTRUCTURE

1 THR 1 TEMP 0 **** **** 0 ROOT

@<TRIPOS>MOLECULE

TRP

27 28 1 0 0

SMALL

No Charge or Current Charge

@<TRIPOS>ATOM

1 N 1.9500 0.6100 1.4600 N3 1 TRP -0.473274

2 H1 1.3000 1.3700 1.3600 H 1 TRP 0.309361

3 H2 1.7500 0.1300 2.3200 H 1 TRP 0.309361

4 H3 2.9500 0.9000 1.4600 H 1 TRP 0.309361

5 CA 1.9600 -0.3100 0.2600 CT 1 TRP 0.116681

6 HA 1.5100 -1.2500 0.5600 HP 1 TRP 0.063548

7 CB 1.2100 0.3200 -0.9100 CT 1 TRP -0.301562

8 HB1 1.7700 1.1800 -1.2600 HC 1 TRP 0.145974

9 HB2 1.2500 -0.4100 -1.7100 HC 1 TRP 0.145974

10 CG -0.2000 0.7200 -0.5900 C* 1 TRP -0.066098

11 CD1 -0.6800 1.9900 -0.5400 CW 1 TRP -0.100263

12 HD1 -0.1800 2.9100 -0.7500 H4 1 TRP 0.183873

13 NE1 -2.0100 1.9900 -0.1900 NA 1 TRP -0.558186

14 HE1 -2.6000 2.7900 -0.1600 H 1 TRP 0.415878

15 CE2 -2.4200 0.6900 -0.0300 CN 1 TRP 0.286183

16 CD2 -1.3200 -0.1400 -0.2700 CB 1 TRP 0.059009

17 CE3 -1.4800 -1.5300 -0.1800 CA 1 TRP -0.190311

18 HE3 -0.6600 -2.1900 -0.3800 HA 1 TRP 0.168086

19 CZ3 -2.7100 -2.0400 0.1500 CA 1 TRP -0.223693

20 HZ3 -2.8500 -3.1000 0.2200 HA 1 TRP 0.158358

21 CH2 -3.8000 -1.1900 0.3900 CA 1 TRP -0.090340

22 HH2 -4.7600 -1.6100 0.6500 HA 1 TRP 0.144102

23 CZ -3.6800 0.1800 0.3100 CA 1 TRP -0.325188

24 HZ2 -4.5100 0.8300 0.4900 HA 1 TRP 0.176673

25 C 3.4800 -0.5100 -0.0700 C 1 TRP 0.778903

26 O 3.7000 -1.3200 -0.9400 O2 1 TRP -0.721206

27 OXT 4.2200 0.2300 0.5900 O2 1 TRP -0.721206

@<TRIPOS>BOND

1 1 2 1

2 1 3 1

3 1 4 1

4 1 5 1

5 5 6 1

6 5 7 1

7 5 25 1

8 7 8 1

9 7 9 1

10 7 10 1

11 10 11 2

12 10 16 1

13 11 12 1

14 11 13 1

15 13 14 1

16 13 15 1

17 15 16 1

18 15 23 2

19 16 17 2

20 17 18 1

21 17 19 1

22 19 20 1

23 19 21 2

24 21 22 1

25 21 23 1

26 23 24 1

27 25 26 1

28 25 27 1

@<TRIPOS>SUBSTRUCTURE

1 TRP 1 TEMP 0 **** **** 0 ROOT

@<TRIPOS>MOLECULE

TYR

24 24 1 0 0

SMALL

No Charge or Current Charge

@<TRIPOS>ATOM

1 N -3.0060 1.4980 -0.3880 N3 1 TYR -0.594139

2 H1 -2.8410 2.2180 -1.0690 H 1 TYR 0.338577

3 H2 -3.2050 1.9290 0.4990 H 1 TYR 0.338577

4 H3 -3.8110 0.8740 -0.6340 H 1 TYR 0.338577

5 CA -1.9110 0.4590 -0.2860 CT 1 TYR 0.228627

6 HA -1.4340 0.4440 -1.2560 HP 1 TYR 0.041362

7 CB -0.8920 0.7960 0.8160 CT 1 TYR -0.066563

8 HB2 -0.9270 1.8640 1.0200 HC 1 TYR 0.039314

9 HB3 -1.1900 0.2870 1.7250 HC 1 TYR 0.039314

10 CG 0.5400 0.4410 0.4620 CA 1 TYR -0.008434

11 CD1 0.9580 -0.8820 0.3650 CA 1 TYR -0.164105

12 HD1 0.2470 -1.6690 0.5280 HA 1 TYR 0.183054

13 CE1 2.2680 -1.1890 0.0430 CA 1 TYR -0.347142

14 HE1 2.5730 -2.2200 -0.0290 HA 1 TYR 0.185577

15 CZ 3.1880 -0.1770 -0.1860 CA 1 TYR 0.474831

16 OH 4.4790 -0.4240 -0.5030 OH 1 TYR -0.618585

17 HH 4.6410 -1.3560 -0.5330 HO 1 TYR 0.422977

18 CE2 2.7900 1.1470 -0.0930 CA 1 TYR -0.347142

19 HE2 3.5090 1.9250 -0.2690 HA 1 TYR 0.185577

20 CD2 1.4770 1.4410 0.2290 CA 1 TYR -0.164105

21 HD2 1.1860 2.4750 0.3050 HA 1 TYR 0.183054

22 C -2.7100 -0.8860 -0.1060 C 1 TYR 0.703978

23 O -2.0710 -1.8260 0.3100 O2 1 TYR -0.696592

24 OXT -3.9010 -0.7700 -0.4360 O2 1 TYR -0.696592

@<TRIPOS>BOND

1 1 2 1

2 1 3 1

3 1 4 1

4 1 5 1

5 5 6 1

6 5 7 1

7 5 22 1

8 7 8 1

9 7 9 1

10 7 10 1

11 10 11 1

12 10 20 2

13 11 12 1

14 11 13 2

15 13 14 1

16 13 15 1

17 15 16 1

18 15 18 2

19 16 17 1

20 18 19 1

21 18 20 1

22 20 21 1

23 22 23 1

24 22 24 1

@<TRIPOS>SUBSTRUCTURE

1 TYR 1 TEMP 0 **** **** 0 ROOT

@<TRIPOS>MOLECULE

VAL

19 18 1 0 0

SMALL

No Charge or Current Charge

@<TRIPOS>ATOM

1 N 3.5400 1.4200 0.0000 N3 1 VAL -0.500554

2 H1 3.8440 2.0630 -0.7090 H 1 VAL 0.315299

3 H2 2.4870 1.3420 0.0790 H 1 VAL 0.315299

4 H3 3.9000 1.7240 0.8890 H 1 VAL 0.315299

5 CA 3.8430 -0.0360 -0.2810 CT 1 VAL 0.027771

6 HA 3.9700 -0.1090 -1.3550 HP 1 VAL 0.082458

7 CB 5.1190 -0.5080 0.4300 CT 1 VAL 0.367144

8 HB 4.9040 -0.5600 1.4960 HC 1 VAL -0.015019

9 CG1 5.5210 -1.9090 -0.0440 CT 1 VAL -0.427697

10 HG12 4.7210 -2.6140 0.1060 HC 1 VAL 0.101460

11 H4 5.7750 -1.8940 -1.1020 HC 1 VAL 0.101460

12 H5 6.4000 -2.2400 0.5000 HC 1 VAL 0.101460

13 CG2 6.2910 0.4620 0.2130 CT 1 VAL -0.427697

14 HG21 7.1860 0.0780 0.6870 HC 1 VAL 0.101460

15 HG22 6.5070 0.5820 -0.8460 HC 1 VAL 0.101460

16 HG23 6.1190 1.4520 0.6320 HC 1 VAL 0.101460

17 C 2.4870 -0.7540 0.1000 C 1 VAL 0.751854

18 O 2.5080 -1.9590 0.1490 O2 1 VAL -0.706459

19 OXT 1.5640 0.0660 0.2740 O2 1 VAL -0.706459

@<TRIPOS>BOND

1 2 1 1

2 3 1 1

3 4 1 1

4 5 1 1

5 6 5 1

6 7 5 1

7 8 7 1

8 9 7 1

9 10 9 1

10 11 9 1

11 12 9 1

12 13 7 1

13 14 13 1

14 15 13 1

15 16 13 1

16 17 5 1

17 18 17 1

18 19 17 1

@<TRIPOS>SUBSTRUCTURE

1 VAL 1 TEMP 0 **** **** 0 ROOT

**Amino acids analogs:**

@<TRIPOS>MOLECULE

ALA

5 4 1 0 0

SMALL

No Charge or Current Charge

@<TRIPOS>ATOM

1 C1 3.5400 1.4200 0.0000 CT 1 ALA -0.452671

2 H1 2.6910 1.4680 -0.6710 HC 1 ALA 0.113168

3 H2 4.4570 1.5030 -0.5710 HC 1 ALA 0.113168

4 H3 3.4830 2.2350 0.7120 HC 1 ALA 0.113168

5 H4 3.5290 0.4740 0.5300 HC 1 ALA 0.113168

@<TRIPOS>BOND

1 2 1 1

2 3 1 1

3 4 1 1

4 5 1 1

@<TRIPOS>SUBSTRUCTURE

1 ALA 1 TEMP 0 **** **** 0 ROOT

@<TRIPOS>MOLECULE

ARG

19 18 1 0 0

SMALL

No Charge or Current Charge

@<TRIPOS>ATOM

1 C1 3.5400 1.4200 0.0000 CT 1 ARG -0.350982

2 H1 2.6890 0.7500 -0.0000 HC 1 ARG 0.107790

3 H2 3.4640 2.0510 0.8790 HC 1 ARG 0.107790

4 H3 3.4640 2.0510 -0.8790 HC 1 ARG 0.107790

5 C2 4.8410 0.6190 -0.0000 CT 1 ARG 0.077416

6 H4 4.8770 -0.0240 0.8760 HC 1 ARG 0.043630

7 H5 4.8770 -0.0240 -0.8760 HC 1 ARG 0.043630

8 C3 6.0640 1.5310 0.0000 CT 1 ARG 0.126610

9 H6 6.0570 2.1620 -0.8830 H1 1 ARG 0.054297

10 H7 6.0570 2.1620 0.8820 H1 1 ARG 0.054297

11 N1 7.2920 0.7200 -0.0000 N2 1 ARG -0.665107

12 H8 7.1790 -0.2690 0.0000 H 1 ARG 0.350059

13 C4 8.5230 1.1890 0.0000 CA 1 ARG 1.089700

14 N3 9.5560 0.3550 -0.0000 N2 1 ARG -1.052447

15 H9 9.4280 -0.6320 0.0000 H 1 ARG 0.489494

16 H10 10.4950 0.6870 0.0000 H 1 ARG 0.489494

17 N2 8.7480 2.4950 -0.0000 N2 1 ARG -1.052447

18 H11 9.6750 2.8610 0.0000 H 1 ARG 0.489494

19 H12 8.0030 3.1530 -0.0010 H 1 ARG 0.489494

@<TRIPOS>BOND

1 2 1 1

2 3 1 1

3 4 1 1

4 5 1 1

5 6 5 1

6 7 5 1

7 8 5 1

8 9 8 1

9 10 8 1

10 11 8 1

11 12 11 1

12 13 11 2

13 14 13 1

14 15 14 1

15 16 14 1

16 17 13 1

17 18 17 1

18 19 17 1

@<TRIPOS>SUBSTRUCTURE

1 ARG 1 TEMP 0 **** **** 0 ROOT

@<TRIPOS>MOLECULE

ASP

10 9 1 0 0

SMALL

No Charge or Current Charge

@<TRIPOS>ATOM

1 C1 3.5400 1.4200 0.0000 CT 1 ASP -0.055980

2 H1 2.5240 1.4200 0.3960 HC 1 ASP -0.010284

3 H2 3.6570 2.2990 -0.6280 HC 1 ASP -0.010284

4 H3 3.6570 0.5420 -0.6290 HC 1 ASP -0.010284

5 C2 4.5970 1.4200 1.1080 CT 1 ASP 0.106280

6 H4 4.4690 2.3030 1.7290 HC 1 ASP -0.085975

7 H5 4.4690 0.5360 1.7290 HC 1 ASP -0.085975

8 C3 6.0210 1.4200 0.4870 C 1 ASP 0.792598

9 O2 6.4930 0.3030 0.2540 O2 1 ASP -0.820049

10 O1 6.4930 2.5370 0.2540 O2 1 ASP -0.820049

@<TRIPOS>BOND

1 2 1 1

2 3 1 1

3 4 1 1

4 5 1 1

5 6 5 1

6 7 5 1

7 8 5 1

8 9 8 1

9 10 8 1

@<TRIPOS>SUBSTRUCTURE

1 ASP 1 TEMP 0 **** **** 0 ROOT

@<TRIPOS>MOLECULE

CYS

9 8 1 0 0

SMALL

No Charge or Current Charge

@<TRIPOS>ATOM

1 C1 3.5400 1.4200 0.0000 CT 1 CYS -0.092561

2 H1 2.5940 1.9500 0.0000 HC 1 CYS 0.048395

3 H2 3.5710 0.7850 0.8790 HC 1 CYS 0.048395

4 H3 3.5710 0.7850 -0.8790 HC 1 CYS 0.048395

5 C2 4.6940 2.4180 0.0000 CT 1 CYS 0.056653

6 H4 4.6440 3.0490 -0.8780 H1 1 CYS 0.035667

7 H5 4.6440 3.0490 0.8780 H1 1 CYS 0.035667

8 S1 6.2850 1.5200 0.0000 SH 1 CYS -0.380403

9 H6 7.0930 2.5730 0.0000 HS 1 CYS 0.199792

@<TRIPOS>BOND

1 2 1 1

2 3 1 1

3 4 1 1

4 5 1 1

5 6 5 1

6 7 5 1

7 8 5 1

8 9 8 1

@<TRIPOS>SUBSTRUCTURE

1 CYS 1 TEMP 0 **** **** 0 ROOT

@<TRIPOS>MOLECULE

GLU

13 12 1 0 0

SMALL

No Charge or Current Charge

@<TRIPOS>ATOM

1 C1 3.5400 1.4200 0.0000 CT 1 GLU -0.283622

2 H1 2.8350 1.4210 -0.8310 HC 1 GLU 0.039306

3 H2 3.3300 0.5430 0.6090 HC 1 GLU 0.039306

4 H3 3.3300 2.2970 0.6100 HC 1 GLU 0.039306

5 C2 4.9920 1.4200 -0.4830 CT 1 GLU 0.277800

6 H4 5.1660 0.5480 -1.1080 HC 1 GLU -0.052004

7 H5 5.1670 2.2930 -1.1070 HC 1 GLU -0.052004

8 C3 6.0200 1.4200 0.6520 CT 1 GLU -0.178394

9 H6 5.8730 2.3030 1.2700 HC 1 GLU -0.002910

10 H7 5.8730 0.5350 1.2690 HC 1 GLU -0.002910

11 C4 7.4610 1.4200 0.0720 C 1 GLU 0.824468

12 O2 7.9390 0.3020 -0.1470 O2 1 GLU -0.824171

13 O1 7.9400 2.5370 -0.1470 O2 1 GLU -0.824171

@<TRIPOS>BOND

1 2 1 1

2 3 1 1

3 4 1 1

4 5 1 1

5 6 5 1

6 7 5 1

7 8 5 1

8 9 8 1

9 10 8 1

10 11 8 1

11 12 11 1

12 13 11 1

@<TRIPOS>SUBSTRUCTURE

1 GLU 1 TEMP 0 **** **** 0 ROOT

@<TRIPOS>MOLECULE

HIS

12 12 1 0 0

SMALL

No Charge or Current Charge

@<TRIPOS>ATOM

1 C1 3.5400 1.4200 0.0000 CT 1 HIS -0.441210

2 H1 3.9090 0.8970 -0.8780 HC 1 HIS 0.136179

3 H2 3.9700 2.4130 0.0000 HC 1 HIS 0.136179

4 H3 3.9090 0.8970 0.8780 HC 1 HIS 0.136179

5 C2 2.0490 1.5140 0.0000 CC 1 HIS 0.142915

6 N1 1.2280 0.4110 0.0000 NA 1 HIS -0.394422

7 H5 1.5160 -0.5420 0.0000 H 1 HIS 0.349362

8 C4 -0.0470 0.8610 0.0000 CR 1 HIS 0.214213

9 H6 -0.8870 0.1960 0.0000 H5 1 HIS 0.117663

10 N2 -0.1070 2.1450 0.0000 NB 1 HIS -0.541298

11 C3 1.2010 2.5660 0.0000 CV 1 HIS 0.015838

12 H4 1.4500 3.6070 0.0000 H4 1 HIS 0.128403

@<TRIPOS>BOND

1 2 1 1

2 3 1 1

3 4 1 1

4 5 1 1

5 6 5 1

6 7 6 1

7 8 6 1

8 9 8 1

9 10 8 2

10 11 10 1

11 12 11 1

12 11 5 2

@<TRIPOS>SUBSTRUCTURE

1 HIS 1 TEMP 0 **** **** 0 ROOT

@<TRIPOS>MOLECULE

ILE

17 16 1 0 0

SMALL

No Charge or Current Charge

@<TRIPOS>ATOM

1 C1 3.5400 1.4200 0.0000 CT 1 ILE -0.452063

2 H1 2.6590 1.8520 -0.4640 HC 1 ILE 0.103611

3 H2 3.6330 0.3970 -0.3530 HC 1 ILE 0.103611

4 H3 3.3640 1.3850 1.0730 HC 1 ILE 0.103611

5 C2 4.7950 2.2370 -0.3220 CT 1 ILE 0.379631

6 H4 4.9220 2.2370 -1.4040 HC 1 ILE -0.037201

7 C3 4.6140 3.6900 0.1310 CT 1 ILE -0.452063

8 H7 3.7170 4.1210 -0.3030 HC 1 ILE 0.103611

9 H8 4.5190 3.7490 1.2130 HC 1 ILE 0.103611

10 H9 5.4490 4.3160 -0.1640 HC 1 ILE 0.103611

11 C4 6.0370 1.5770 0.2960 CT 1 ILE -0.067084

12 H5 5.9420 1.5860 1.3800 HC 1 ILE 0.016026

13 H6 6.0510 0.5300 0.0020 HC 1 ILE 0.016026

14 C5 7.3710 2.2100 -0.1030 CT 1 ILE -0.100304

15 H10 7.4830 2.2410 -1.1830 HC 1 ILE 0.025122

16 H11 7.4690 3.2250 0.2690 HC 1 ILE 0.025122

17 H12 8.2020 1.6370 0.2980 HC 1 ILE 0.025122

@<TRIPOS>BOND

1 2 1 1

2 3 1 1

3 4 1 1

4 5 1 1

5 6 5 1

6 7 5 1

7 8 7 1

8 9 7 1

9 10 7 1

10 11 5 1

11 12 11 1

12 13 11 1

13 14 11 1

14 15 14 1

15 16 14 1

16 17 14 1

@<TRIPOS>SUBSTRUCTURE

1 ILE 1 TEMP 0 **** **** 0 ROOT

@<TRIPOS>MOLECULE

LYS

20 19 1 0 0

SMALL

No Charge or Current Charge

@<TRIPOS>ATOM

1 C1 3.5400 1.4200 0.0000 CT 1 LYS -0.256254

2 H1 4.3760 0.7300 0.0010 HC 1 LYS 0.073721

3 H2 3.6320 2.0510 -0.8780 HC 1 LYS 0.073721

4 H3 3.6320 2.0520 0.8780 HC 1 LYS 0.073721

5 C2 2.2140 0.6610 -0.0000 CT 1 LYS 0.158255

6 H4 2.1670 0.0120 -0.8710 HC 1 LYS -0.006555

7 H5 2.1670 0.0130 0.8720 HC 1 LYS -0.006555

8 C3 1.0050 1.5990 0.0000 CT 1 LYS -0.073182

9 H6 1.0470 2.2460 0.8720 HC 1 LYS 0.032774

10 H7 1.0480 2.2450 -0.8730 HC 1 LYS 0.032774

11 C4 -0.3250 0.8340 -0.0010 CT 1 LYS -0.099923

12 H8 -0.3750 0.1920 -0.8770 HC 1 LYS 0.046170

13 H9 -0.3750 0.1910 0.8750 HC 1 LYS 0.046170

14 C5 -1.5100 1.7890 0.0000 CT 1 LYS 0.219143

15 H10 -1.5360 2.4150 0.8810 HP 1 LYS 0.058160

16 H11 -1.5370 2.4150 -0.8800 HP 1 LYS 0.058160

17 N1 -2.8160 1.0110 0.0000 N3 1 LYS -0.487261

18 H12 -2.8870 0.4140 -0.8120 H 1 LYS 0.352321

19 H13 -2.8890 0.4170 0.8150 H 1 LYS 0.352321

20 H14 -3.6200 1.6230 -0.0020 H 1 LYS 0.352321

@<TRIPOS>BOND

1 2 1 1

2 3 1 1

3 4 1 1

4 5 1 1

5 6 5 1

6 7 5 1

7 8 5 1

8 9 8 1

9 10 8 1

10 11 8 1

11 12 11 1

12 13 11 1

13 14 11 1

14 15 14 1

15 16 14 1

16 17 14 1

17 18 17 1

18 19 17 1

19 20 17 1

@<TRIPOS>SUBSTRUCTURE

1 LYS 1 TEMP 0 **** **** 0 ROOT

@<TRIPOS>MOLECULE

PHE

18 18 1 0 0

SMALL

No Charge or Current Charge

@<TRIPOS>ATOM

1 C1 3.5400 1.4200 0.0000 CT 1 PHE -0.046666

2 H1 4.6020 1.4200 -0.2290 HC 1 PHE 0.013122

3 H2 3.3250 2.2950 0.6040 HC 1 PHE 0.013122

4 H3 3.3250 0.5420 0.6020 HC 1 PHE 0.013122

5 C2 2.7050 1.4210 -1.2860 CT 1 PHE 0.056395

6 H4 2.9650 2.2910 -1.8810 HC 1 PHE 0.003327

7 H5 2.9650 0.5510 -1.8820 HC 1 PHE 0.003327

8 C3 1.2140 1.4200 -1.0180 CA 1 PHE 0.026163

9 C4 0.5140 2.6140 -0.8770 CA 1 PHE -0.163452

10 H8 1.0340 3.5490 -0.9880 HA 1 PHE 0.138114

11 C7 -0.8430 2.6160 -0.6000 CA 1 PHE -0.160698

12 H9 -1.3660 3.5510 -0.4970 HA 1 PHE 0.137084

13 C8 -1.5270 1.4200 -0.4590 CA 1 PHE -0.108616

14 H10 -2.5810 1.4200 -0.2470 HA 1 PHE 0.124608

15 C6 -0.8430 0.2240 -0.6000 CA 1 PHE -0.160698

16 H7 -1.3650 -0.7110 -0.4970 HA 1 PHE 0.137084

17 C5 0.5150 0.2270 -0.8770 CA 1 PHE -0.163452

18 H6 1.0350 -0.7100 -0.9890 HA 1 PHE 0.138114

@<TRIPOS>BOND

1 2 1 1

2 3 1 1

3 4 1 1

4 5 1 1

5 6 5 1

6 7 5 1

7 8 5 1

8 9 8 1

9 10 9 1

10 11 9 2

11 12 11 1

12 13 11 1

13 14 13 1

14 15 13 2

15 16 15 1

16 17 15 1

17 18 17 1

18 17 8 2

@<TRIPOS>SUBSTRUCTURE

1 PHE 1 TEMP 0 **** **** 0 ROOT

@<TRIPOS>MOLECULE

SER

9 8 1 0 0

SMALL

No Charge or Current Charge

@<TRIPOS>ATOM

1 C1 3.5400 1.4200 0.0000 CT 1 SER -0.249450

2 H1 4.3810 2.1070 0.0000 HC 1 SER 0.063834

3 H2 3.6090 0.7890 0.8790 HC 1 SER 0.063834

4 H3 3.6090 0.7880 -0.8790 HC 1 SER 0.063834

5 C2 2.2290 2.1810 0.0000 CT 1 SER 0.518296

6 H4 2.1700 2.8220 0.8780 H1 1 SER -0.081960

7 H5 2.1700 2.8220 -0.8780 H1 1 SER -0.081960

8 O1 1.1830 1.2440 0.0000 OH 1 SER -0.711178

9 H6 0.3510 1.6960 -0.0000 HO 1 SER 0.414749

@<TRIPOS>BOND

1 2 1 1

2 3 1 1

3 4 1 1

4 5 1 1

5 6 5 1

6 7 5 1

7 8 5 1

8 9 8 1

@<TRIPOS>SUBSTRUCTURE

1 SER 1 TEMP 0 **** **** 0 ROOT

@<TRIPOS>MOLECULE

TRP

22 23 1 0 0

SMALL

No Charge or Current Charge

@<TRIPOS>ATOM

1 C1 3.5400 1.4200 0.0000 CT 1 TRP -0.100471

2 H1 2.6170 0.8920 -0.2220 HC 1 TRP 0.021958

3 H2 4.2710 0.6910 0.3320 HC 1 TRP 0.021958

4 H3 3.3520 2.0980 0.8260 HC 1 TRP 0.021958

5 C2 4.0370 2.1890 -1.2300 CT 1 TRP 0.161878

6 H4 4.1600 1.4980 -2.0600 HC 1 TRP -0.010611

7 H5 3.2740 2.8990 -1.5380 HC 1 TRP -0.010611

8 C3 5.3270 2.9260 -0.9970 C* 1 TRP -0.233858

9 C4 5.4840 4.2550 -0.8330 CW 1 TRP -0.103744

10 H6 4.7450 5.0310 -0.8520 H4 1 TRP 0.186510

11 N1 6.8060 4.5660 -0.6180 NA 1 TRP -0.500822

12 H11 7.1680 5.4810 -0.4910 H 1 TRP 0.385016

13 C5 7.5410 3.4120 -0.6500 CN 1 TRP 0.273486

14 C6 6.6530 2.3520 -0.8840 CB 1 TRP 0.138092

15 C9 7.1590 1.0510 -0.9720 CA 1 TRP -0.233189

16 H9 6.5020 0.2210 -1.1570 HA 1 TRP 0.178165

17 C10 8.5110 0.8450 -0.8240 CA 1 TRP -0.218018

18 H10 8.9110 -0.1510 -0.8900 HA 1 TRP 0.146168

19 C8 9.3800 1.9200 -0.5860 CA 1 TRP -0.094291

20 H8 10.4340 1.7310 -0.4730 HA 1 TRP 0.136465

21 C7 8.9120 3.2100 -0.4970 CA 1 TRP -0.341550

22 H7 9.5810 4.0340 -0.3180 HA 1 TRP 0.175511

@<TRIPOS>BOND

1 2 1 1

2 3 1 1

3 4 1 1

4 5 1 1

5 6 5 1

6 7 5 1

7 8 5 1

8 9 8 2

9 10 9 1

10 11 9 1

11 12 11 1

12 13 11 1

13 14 13 1

14 15 14 2

15 16 15 1

16 17 15 1

17 18 17 1

18 19 17 2

19 20 19 1

20 21 19 1

21 22 21 1

22 14 8 1

23 21 13 2

@<TRIPOS>SUBSTRUCTURE

1 TRP 1 TEMP 0 **** **** 0 ROOT

@<TRIPOS>MOLECULE

VAL

14 13 1 0 0

SMALL

No Charge or Current Charge

@<TRIPOS>ATOM

1 C1 3.5400 1.4200 0.0000 CT 1 VAL -0.449954

2 H1 3.8770 0.4520 -0.3590 HC 1 VAL 0.099238

3 H2 4.2410 2.1690 -0.3590 HC 1 VAL 0.099238

4 H3 3.5970 1.4070 1.0860 HC 1 VAL 0.099238

5 C2 2.1150 1.7220 -0.4690 CT 1 VAL 0.523104

6 H4 2.1150 1.7220 -1.5580 HC 1 VAL -0.066384

7 C4 1.6640 3.1070 0.0000 CT 1 VAL -0.449954

8 H5 1.6450 3.1630 1.0860 HC 1 VAL 0.099238

9 H6 2.3340 3.8830 -0.3580 HC 1 VAL 0.099238

10 H7 0.6660 3.3400 -0.3590 HC 1 VAL 0.099238

11 C3 1.1410 0.6390 0.0000 CT 1 VAL -0.449954

12 H8 1.4390 -0.3420 -0.3590 HC 1 VAL 0.099238

13 H9 1.1010 0.5950 1.0860 HC 1 VAL 0.099238

14 H10 0.1350 0.8310 -0.3590 HC 1 VAL 0.099238

@<TRIPOS>BOND

1 2 1 1

2 3 1 1

3 4 1 1

4 5 1 1

5 6 5 1

6 7 5 1

7 8 7 1

8 9 7 1

9 10 7 1

10 11 5 1

11 12 11 1

12 13 11 1

13 14 11 1

@<TRIPOS>SUBSTRUCTURE

1 VAL 1 TEMP 0 **** **** 0 ROOT

@<TRIPOS>MOLECULE

ASN

12 11 1 0 0

SMALL

No Charge or Current Charge

@<TRIPOS>ATOM

1 C1 3.5400 1.4200 0.0000 CT 1 ASN -0.148415

2 H1 2.6900 0.7480 0.0460 HC 1 ASN 0.046500

3 H2 3.4880 1.9770 -0.9270 HC 1 ASN 0.046500

4 H3 3.4640 2.1290 0.8140 HC 1 ASN 0.046500

5 C2 4.8410 0.6300 0.0880 CT 1 ASN -0.078582

6 H4 4.8750 0.0540 1.0120 HC 1 ASN 0.032423

7 H5 4.9080 -0.0920 -0.7210 HC 1 ASN 0.032423

8 C3 6.0740 1.5160 0.0470 C 1 ASN 0.855648

9 O1 6.0230 2.7120 0.0640 O 1 ASN -0.613542

10 N1 7.2540 0.8470 -0.0250 N 1 ASN -1.091051

11 H6 7.3080 -0.1360 0.1130 H 1 ASN 0.435799

12 H7 8.0930 1.3760 0.0650 H 1 ASN 0.435799

@<TRIPOS>BOND

1 2 1 1

2 3 1 1

3 4 1 1

4 5 1 1

5 6 5 1

6 7 5 1

7 8 5 1

8 9 8 2

9 10 8 1

10 11 10 1

11 12 10 1

@<TRIPOS>SUBSTRUCTURE

1 ASN 1 TEMP 0 **** **** 0 ROOT

@<TRIPOS>MOLECULE

GLN

15 14 1 0 0

SMALL

No Charge or Current Charge

@<TRIPOS>ATOM

1 C1 3.5400 1.4200 0.0000 CT 1 GLN -0.242969

2 H7 2.7390 2.1430 -0.1090 HC 1 GLN 0.052029

3 H8 3.4200 0.6740 -0.7800 HC 1 GLN 0.052029

4 H9 3.4060 0.9220 0.9560 HC 1 GLN 0.052029

5 C2 4.9050 2.1030 -0.0850 CT 1 GLN 0.276560

6 H1 5.0010 2.6190 -1.0340 HC 1 GLN -0.034639

7 H2 4.9910 2.8620 0.6820 HC 1 GLN -0.034639

8 C3 6.0550 1.1100 0.0640 CT 1 GLN -0.346252

9 H3 5.9800 0.5880 1.0160 HC 1 GLN 0.080902

10 H4 6.0020 0.3450 -0.7070 HC 1 GLN 0.080902

11 C4 7.4250 1.7670 0.0080 C 1 GLN 0.926915

12 O1 7.5870 2.9520 0.0350 O 1 GLN -0.639854

13 N1 8.4680 0.9010 -0.0860 N 1 GLN -1.105144

14 H5 8.3490 -0.0770 0.0410 H 1 GLN 0.441066

15 H6 9.3870 1.2730 0.0010 H 1 GLN 0.441066

@<TRIPOS>BOND

1 2 1 1

2 3 1 1

3 4 1 1

4 5 1 1

5 6 5 1

6 7 5 1

7 8 5 1

8 9 8 1

9 10 8 1

10 11 8 1

11 12 11 2

12 13 11 1

13 14 13 1

14 15 13 1

@<TRIPOS>SUBSTRUCTURE

1 GLN 1 TEMP 0 **** **** 0 ROOT

@<TRIPOS>MOLECULE

LEU

17 16 1 0 0

SMALL

No Charge or Current Charge

@<TRIPOS>ATOM

1 C1 3.5400 1.4200 0.0000 CT 1 LEU -0.100315

2 H1 4.3710 0.8470 0.4010 HC 1 LEU 0.025124

3 H2 3.6520 1.4510 -1.0800 HC 1 LEU 0.025124

4 H3 3.6380 2.4340 0.3720 HC 1 LEU 0.025124

5 C2 2.2060 0.7870 0.3990 CT 1 LEU -0.067072

6 H4 2.2200 -0.2610 0.1050 HC 1 LEU 0.016023

7 H5 2.1110 0.7960 1.4830 HC 1 LEU 0.016023

8 C3 0.9640 1.4470 -0.2190 CT 1 LEU 0.379640

9 H6 1.0920 1.4470 -1.3010 HC 1 LEU -0.037204

10 C5 -0.2910 0.6290 0.1030 CT 1 LEU -0.452072

11 H7 -0.1990 -0.3930 -0.2500 HC 1 LEU 0.103613

12 H8 -0.4670 0.5930 1.1760 HC 1 LEU 0.103613

13 H9 -1.1720 1.0610 -0.3610 HC 1 LEU 0.103613

14 C4 0.7830 2.9000 0.2340 CT 1 LEU -0.452072

15 H10 0.6880 2.9590 1.3170 HC 1 LEU 0.103613

16 H11 1.6170 3.5260 -0.0600 HC 1 LEU 0.103613

17 H12 -0.1140 3.3310 -0.2000 HC 1 LEU 0.103613

@<TRIPOS>BOND

1 2 1 1

2 3 1 1

3 4 1 1

4 5 1 1

5 6 5 1

6 7 5 1

7 8 5 1

8 9 8 1

9 10 8 1

10 11 10 1

11 12 10 1

12 13 10 1

13 14 8 1

14 15 14 1

15 16 14 1

16 17 14 1

@<TRIPOS>SUBSTRUCTURE

1 LEU 1 TEMP 0 **** **** 0 ROOT

@<TRIPOS>MOLECULE

MET

15 14 1 0 0

SMALL

No Charge or Current Charge

@<TRIPOS>ATOM

1 C1 3.5400 1.4200 0.0000 CT 1 MET -0.296724

2 H1 2.6610 0.7830 -0.0000 HC 1 MET 0.067212

3 H2 3.4870 2.0570 0.8780 HC 1 MET 0.067212

4 H3 3.4870 2.0570 -0.8780 HC 1 MET 0.067212

5 C2 4.8190 0.5820 -0.0000 CT 1 MET 0.248934

6 H4 4.8320 -0.0660 0.8720 HC 1 MET -0.000589

7 H5 4.8320 -0.0660 -0.8710 HC 1 MET -0.000589

8 C3 6.0740 1.4540 0.0000 CT 1 MET -0.270132

9 H6 6.0850 2.0930 -0.8770 H1 1 MET 0.125714

10 H7 6.0850 2.0930 0.8770 H1 1 MET 0.125714

11 S1 7.5730 0.4270 -0.0000 S 1 MET -0.281885

12 C4 8.8430 1.7150 0.0000 CT 1 MET -0.071094

13 H8 8.7710 2.3360 -0.8840 H1 1 MET 0.073005

14 H9 8.7710 2.3360 0.8840 H1 1 MET 0.073005

15 H10 9.8060 1.2220 -0.0000 H1 1 MET 0.073005

@<TRIPOS>BOND

1 2 1 1

2 3 1 1

3 4 1 1

4 5 1 1

5 6 5 1

6 7 5 1

7 8 5 1

8 9 8 1

9 10 8 1

10 11 8 1

11 12 11 1

12 13 12 1

13 14 12 1

14 15 12 1

@<TRIPOS>SUBSTRUCTURE

1 MET 1 TEMP 0 **** **** 0 ROOT

@<TRIPOS>MOLECULE

PRO

14 14 1 0 0

SMALL

No Charge or Current Charge

@<TRIPOS>ATOM

1 C1 3.5400 1.4200 0.0000 CT 1 PRO -0.085141

2 H4 3.9180 1.7690 -0.9530 HC 1 PRO 0.035683

3 H5 3.9550 2.0610 0.7690 HC 1 PRO 0.035683

4 C2 1.9900 1.4200 -0.0010 CT 1 PRO -0.085141

5 H6 1.5730 2.0610 0.7670 HC 1 PRO 0.035683

6 H7 1.6120 1.7670 -0.9550 HC 1 PRO 0.035683

7 C4 1.6080 -0.0520 0.2200 CT 1 PRO 0.249529

8 H8 1.4390 -0.2390 1.2840 H1 1 PRO -0.018669

9 H9 0.7100 -0.3400 -0.3120 H1 1 PRO -0.018669

10 N1 2.7650 -0.7690 -0.2890 NT 1 PRO -0.745333

11 H1 2.7650 -1.7340 -0.0260 H 1 PRO 0.348499

12 C3 3.9220 -0.0520 0.2200 CT 1 PRO 0.249529

13 H2 4.8190 -0.3390 -0.3140 H1 1 PRO -0.018669

14 H3 4.0930 -0.2400 1.2830 H1 1 PRO -0.018669

@<TRIPOS>BOND

1 2 1 1

2 3 1 1

3 4 1 1

4 5 4 1

5 6 4 1

6 7 4 1

7 8 7 1

8 9 7 1

9 10 7 1

10 11 10 1

11 12 10 1

12 13 12 1

13 14 12 1

14 12 1 1

@<TRIPOS>SUBSTRUCTURE

1 PRO 1 TEMP 0 **** **** 0 ROOT

@<TRIPOS>MOLECULE

THR

12 11 1 0 0

SMALL

No Charge or Current Charge

@<TRIPOS>ATOM

1 C1 3.5400 1.4200 0.0000 CT 1 THR -0.428223

2 H1 3.6700 0.4210 0.4050 HC 1 THR 0.102698

3 H2 3.5770 1.3620 -1.0830 HC 1 THR 0.102698

4 H3 4.3640 2.0390 0.3350 HC 1 THR 0.102698

5 C2 2.2170 2.0180 0.4480 CT 1 THR 0.637953

6 H4 2.2090 2.0700 1.5370 H1 1 THR -0.075361

7 O1 2.1670 3.3220 -0.0830 OH 1 THR -0.742492

8 H5 1.3750 3.7530 0.2090 HO 1 THR 0.420161

9 C3 1.0210 1.1940 -0.0140 CT 1 THR -0.428223

10 H6 1.0060 1.1260 -1.0970 HC 1 THR 0.102698

11 H7 1.0550 0.1890 0.3960 HC 1 THR 0.102698

12 H8 0.0880 1.6500 0.3080 HC 1 THR 0.102698

@<TRIPOS>BOND

1 2 1 1

2 3 1 1

3 4 1 1

4 5 1 1

5 6 5 1

6 7 5 1

7 8 7 1

8 9 5 1

9 10 9 1

10 11 9 1

11 12 9 1

@<TRIPOS>SUBSTRUCTURE

1 THR 1 TEMP 0 **** **** 0 ROOT

@<TRIPOS>MOLECULE

TYR

19 19 1 0 0

SMALL

No Charge or Current Charge

@<TRIPOS>ATOM

1 C1 3.5400 1.4200 0.0000 CT 1 TYR -0.082247

2 H1 4.6110 1.4350 -0.1790 HC 1 TYR 0.019609

3 H2 3.2940 2.2610 0.6410 HC 1 TYR 0.019609

4 H3 3.3030 0.5100 0.5440 HC 1 TYR 0.019609

5 C2 2.7620 1.4910 -1.3190 CT 1 TYR 0.113315

6 H4 3.0460 2.3940 -1.8530 HC 1 TYR -0.009448

7 H5 3.0560 0.6580 -1.9520 HC 1 TYR -0.009448

8 C3 1.2610 1.4720 -1.1260 CA 1 TYR -0.043917

9 C4 0.5330 2.6450 -1.0070 CA 1 TYR -0.132134

10 H8 1.0370 3.5940 -1.0810 HA 1 TYR 0.152367

11 C7 -0.8390 2.6320 -0.7990 CA 1 TYR -0.346322

12 H9 -1.3800 3.5600 -0.7140 HA 1 TYR 0.184520

13 C8 -1.5100 1.4250 -0.7050 CA 1 TYR 0.437979

14 O1 -2.8450 1.3440 -0.5050 OH 1 TYR -0.561879

15 H10 -3.2250 2.2090 -0.4410 HO 1 TYR 0.379958

16 C6 -0.8030 0.2370 -0.8230 CA 1 TYR -0.346322

17 H7 -1.3340 -0.6950 -0.7540 HA 1 TYR 0.184520

18 C5 0.5620 0.2700 -1.0300 CA 1 TYR -0.132134

19 H6 1.0950 -0.6610 -1.1240 HA 1 TYR 0.152367

@<TRIPOS>BOND

1 2 1 1

2 3 1 1

3 4 1 1

4 5 1 1

5 6 5 1

6 7 5 1

7 8 5 1

8 9 8 1

9 10 9 1

10 11 9 2

11 12 11 1

12 13 11 1

13 14 13 1

14 15 14 1

15 16 13 2

16 17 16 1

17 18 16 1

18 19 18 1

19 18 8 2

@<TRIPOS>SUBSTRUCTURE

1 TYR 1 TEMP 0 **** **** 0 ROOT

**Supplementary Text 2**

The atom type from CHARMM force field (CHARMM22 force field) and charge of the amino acid analog molecules modeled using quantum chemical calculations is described below in GROMACS rtp format.

[ bondedtypes ]

; Column 1 : default bondtype

; Column 2 : default angletype

; Column 3 : default proper dihedraltype

; Column 4 : default improper dihedraltype

; Column 5 : This controls the generation of dihedrals from the bonding.

; All possible dihedrals are generated automatically. A value of

; 1 here means that all these are retained. A value of

; 0 here requires generated dihedrals be removed if

; * there are any dihedrals on the same central atoms

; specified in the residue topology, or

; * there are other identical generated dihedrals

; sharing the same central atoms, or

; * there are other generated dihedrals sharing the

; same central bond that have fewer hydrogen atoms

; Column 6 : number of neighbors to exclude from non-bonded interactions

; Column 7 : 1 = generate 1,4 interactions between pairs of hydrogen atoms

; 0 = do not generate such

; Column 8 : 1 = remove proper dihedrals if found centered on the same

; bond as an improper dihedral

; 0 = do not generate such

; bondtype angletype dihedraltype impropertype all_dih nrexcl HH14 bRemoveDih

1 5 9 2 1 3 1 0

[ ALA ]

[ atoms ]

CB CT3 -0.36 0

HB1 HA 0.09 1

HB2 HA 0.09 2

HB3 HA 0.09 3

HB4 HA 0.09 4

[ bonds ]

CB HB1

CB HB2

CB HB3

CB HB4

[ ARG ]

[ atoms ]

HB3 HA 0.09 0

CB CT3 -0.27 1

HB1 HA 0.09 2

HB2 HA 0.09 3

CG CT2 -0.18 4

HG1 HA 0.09 5

HG2 HA 0.09 6

CD CT2 0.20 7

HD1 HA 0.09 8

HD2 HA 0.09 9

NE NC2 -0.70 10

HE HC 0.44 11

CZ C 0.64 12

NH1 NC2 -0.80 13

HH11 HC 0.46 14

HH12 HC 0.46 15

NH2 NC2 -0.80 16

HH21 HC 0.46 17

HH22 HC 0.46 18

[ bonds ]

CG CB

CD CG

NE CD

CZ NE

NH2 CZ

CB HB1

CB HB2

CB HB3

CG HG1

CG HG2

CD HD1

CD HD2

NE HE

NH1 HH11

NH1 HH12

NH2 HH21

NH2 HH22

CZ NH1

[ impropers ]

CZ NH1 NH2 NE

[ cmap ]

[ ASN ]

[ atoms ]

HB3 HA 0.09 0

CB CT3 -0.27 1

HB1 HA 0.09 2

HB2 HA 0.09 3

CG CC 0.55 4

OD1 O -0.55 5

ND2 NH2 -0.62 6

HD21 H 0.31 7

HD22 H 0.31 8

[ bonds ]

CG CB

ND2 CG

CB HB1

CB HB2

CB HB3

ND2 HD21

ND2 HD22

CG OD1

[ impropers ]

N -C CA HN

C CA +N O

CG ND2 CB OD1

CG CB ND2 OD1

ND2 CG HD21 HD22

ND2 CG HD22 HD21

[ cmap ]

-C N CA C +N

[ ASP ]

[ atoms ]

HB3 HA 0.09 0

CB CT2 -0.37 1

HB1 HA 0.09 2

HB2 HA 0.09 3

CG CC 0.62 4

OD1 OC -0.76 5

OD2 OC -0.76 6

[ bonds ]

CG CB

OD2 CG

CB HB1

CB HB2

CB HB3

CG OD1

[ impropers ]

CG CB OD2 OD1

[ cmap ]

[ CYS ]

[ atoms ]

HB3 HA 0.09 0

CB CT3 -0.20 1

HB1 HA 0.09 2

HB2 HA 0.09 3

SG S -0.23 4

HG1 HS 0.16 5

[ bonds ]

SG CB

CB HB1

CB HB2

CB HB3

SG HG1

[ impropers ]

[ cmap ]

[ GLN ]

[ atoms ]

HB3 HA 0.09 0

CB CT3 -0.27 1

HB1 HA 0.09 2

HB2 HA 0.09 3

CG CT2 -0.18 4

HG1 HA 0.09 5

HG2 HA 0.09 6

CD CC 0.55 7

OE1 O -0.55 8

NE2 NH2 -0.62 9

HE21 H 0.31 10

HE22 H 0.31 11

[ bonds ]

CG CB

CD CG

NE2 CD

CB HB1

CB HB2

CB HB3

CG HG1

CG HG2

NE2 HE21

NE2 HE22

CD OE1

[ impropers ]

CD NE2 CG OE1

CD CG NE2 OE1

NE2 CD HE21 HE22

NE2 CD HE22 HE21

[ cmap ]

[ GLU ]

[ atoms ]

HB3 HA 0.09 1

CB CT3 -0.27 2

HB1 HA 0.09 3

HB2 HA 0.09 4

CG CT2 -0.28 5

HG1 HA 0.09 6

HG2 HA 0.09 7

CD CC 0.62 8

OE1 OC -0.76 9

OE2 OC -0.76 10

[ bonds ]

CG CB

CD CG

OE2 CD

CB HB1

CB HB2

CB HB3

CG HG1

CG HG2

CD OE1

[ impropers ]

CD CG OE2 OE1

[ cmap ]

; coresponding to HSE

[ HIS ]

[ atoms ]

HB3 HA 0.09 0

CB CT3 -0.17 1

HB1 HA 0.09 2

HB2 HA 0.09 3

ND1 NR2 -0.70 4

CG CPH1 0.22 5

CE1 CPH2 0.25 6

HE1 HR1 0.13 7

NE2 NR1 -0.36 8

HE2 H 0.32 9

CD2 CPH1 -0.05 10

HD2 HR3 0.09 11

[ bonds ]

CG CB

ND1 CG

NE2 CD2

NE2 CE1

CB HB1

CB HB2

CB HB3

NE2 HE2

CD2 HD2

CE1 HE1

CD2 CG

CE1 ND1

[ impropers ]

NE2 CD2 CE1 HE2

CD2 CG NE2 HD2

CE1 ND1 NE2 HE1

NE2 CE1 CD2 HE2

CD2 NE2 CG HD2

CE1 NE2 ND1 HE1

[ cmap ]

[ ILE ]

[ atoms ]

HB2 HA 0.09 0

CB CT2 -0.18 1

HB1 HA 0.09 2

CG2 CT3 -0.27 3

HG21 HA 0.09 4

HG22 HA 0.09 5

HG23 HA 0.09 6

CG1 CT2 -0.18 7

HG11 HA 0.09 8

HG12 HA 0.09 9

CD CT3 -0.27 10

HD1 HA 0.09 11

HD2 HA 0.09 12

HD3 HA 0.09 13

[ bonds ]

CG1 CB

CG2 CB

CD CG1

CB HB1

CB HB2

CG1 HG11

CG1 HG12

CG2 HG21

CG2 HG22

CG2 HG23

CD HD1

CD HD2

CD HD3

[ impropers ]

[ cmap ]

[ LEU ]

[ atoms ]

HB3 HA 0.09 0

CB CT3 -0.27 1

HB1 HA 0.09 2

HB2 HA 0.09 3

CG CT1 -0.09 4

HG HA 0.09 5

CD1 CT3 -0.27 6

HD11 HA 0.09 7

HD12 HA 0.09 8

HD13 HA 0.09 9

CD2 CT3 -0.27 10

HD21 HA 0.09 11

HD22 HA 0.09 12

HD23 HA 0.09 13

[ bonds ]

CG CB

CD1 CG

CD2 CG

CB HB1

CB HB2

CB HB3

CG HG

CD1 HD11

CD1 HD12

CD1 HD13

CD2 HD21

CD2 HD22

CD2 HD23

[ impropers ]

[ cmap ]

[ LYS ]

[ atoms ]

HB3 HA 0.09 3

CB CT3 -0.27 4

HB1 HA 0.09 5

HB2 HA 0.09 6

CG CT2 -0.18 7

HG1 HA 0.09 8

HG2 HA 0.09 9

CD CT2 -0.18 10

HD1 HA 0.09 11

HD2 HA 0.09 12

CE CT2 0.21 13

HE1 HA 0.05 14

HE2 HA 0.05 15

NZ NH3 -0.30 16

HZ1 HC 0.33 17

HZ2 HC 0.33 18

HZ3 HC 0.33 19

[ bonds ]

CG CB

CD CG

CE CD

NZ CE

CB HB1

CB HB2

CB HB3

CG HG1

CG HG2

CD HD1

CD HD2

CE HE1

CE HE2

NZ HZ1

NZ HZ2

NZ HZ3

[ impropers ]

[ cmap ]

[ MET ]

[ atoms ]

HB3 HA 0.09 0

CB CT3 -0.27 1

HB1 HA 0.09 2

HB2 HA 0.09 3

CG CT2 -0.14 4

HG1 HA 0.09 5

HG2 HA 0.09 6

SD S -0.09 7

CE CT3 -0.22 8

HE1 HA 0.09 9

HE2 HA 0.09 10

HE3 HA 0.09 11

[ bonds ]

CG CB

SD CG

CE SD

CB HB1

CB HB2

CB HB3

CG HG1

CG HG2

CE HE1

CE HE2

CE HE3

[ impropers ]

[ cmap ]

[ PHE ]

[ atoms ]

HB3 HA 0.09 0

CB CT3 -0.27 1

HB1 HA 0.09 2

HB2 HA 0.09 3

CG CA 0.00 4

CD1 CA -0.115 5

HD1 HP 0.115 6

CE1 CA -0.115 7

HE1 HP 0.115 8

CZ CA -0.115 9

HZ HP 0.115 10

CD2 CA -0.115 11

HD2 HP 0.115 12

CE2 CA -0.115 13

HE2 HP 0.115 14

[ bonds ]

CG CB

CD2 CG

CE1 CD1

CZ CE2

CB HB1

CB HB2

CB HB3

CD1 HD1

CD2 HD2

CE1 HE1

CD1 CG

CZ CE1

CE2 CD2

CE2 HE2

CZ HZ

[ impropers ]

[ cmap ]

[ SER ]

[ atoms ]

HB3 HA 0.09 0

CB CT3 -0.04 1

HB1 HA 0.09 2

HB2 HA 0.09 3

OG OH1 -0.66 4

HG1 H 0.43 5

[ bonds ]

OG CB

CB HB1

CB HB2

CB HB3

OG HG1

[ impropers ]

[ cmap ]

[ THR ]

[ atoms ]

HB2 HA 0.09 0

CB CT2 0.05 1

HB1 HA 0.09 2

OG1 OH1 -0.66 3

HG1 H 0.43 4

CG2 CT3 -0.27 5

HG21 HA 0.09 6

HG22 HA 0.09 7

HG23 HA 0.09 8

[ bonds ]

OG1 CB

CG2 CB

CB HB1

CB HB2

OG1 HG1

CG2 HG21

CG2 HG22

CG2 HG23

[ impropers ]

[ cmap ]

[ TRP ]

[ atoms ]

HB3 HA 0.09 0

CB CT2 -0.27 1

HB1 HA 0.09 2

HB2 HA 0.09 3

CG CY -0.03 4

CD1 CA 0.035 5

HD1 HP 0.115 6

NE1 NY -0.61 7

HE1 H 0.38 8

CE2 CPT 0.13 9

CD2 CPT -0.02 10

CE3 CA -0.115 11

HE3 HP 0.115 12

CZ3 CA -0.115 13

HZ3 HP 0.115 14

CZ2 CA -0.115 15

HZ2 HP 0.115 16

CH2 CA -0.115 17

HH2 HP 0.115 18

[ bonds ]

CG CB

CD2 CG

NE1 CD1

CZ2 CE2

CZ3 CH2

CD2 CE3

NE1 CE2

CB HB1

CB HB2

CB HB3

CD1 HD1

NE1 HE1

CE3 HE3

CZ2 HZ2

CZ3 HZ3

CH2 HH2

CD1 CG

CE2 CD2

CZ3 CE3

CH2 CZ2

[ impropers ]

[ cmap ]

[ TYR ]

[ atoms ]

HB3 HA 0.09 0

CB CT3 -0.27 1

HB1 HA 0.09 2

HB2 HA 0.09 3

CG CA 0.00 4

CD1 CA -0.115 5

HD1 HP 0.115 6

CE1 CA -0.115 7

HE1 HP 0.115 8

CZ CA 0.11 9

OH OH1 -0.54 10

HH H 0.43 11

CD2 CA -0.115 12

HD2 HP 0.115 13

CE2 CA -0.115 14

HE2 HP 0.115 15

[ bonds ]

CG CB

CD2 CG

CE1 CD1

CZ CE2

OH CZ

CB HB1

CB HB2

CB HB3

CD1 HD1

CD2 HD2

CE1 HE1

CE2 HE2

OH HH

CD1 CG

CE1 CZ

CE2 CD2

[ impropers ]

[ cmap ]

[ VAL ]

[ atoms ]

HB2 HA 0.09 0

CB CT2 -0.18 1

HB1 HA 0.09 2

CG1 CT3 -0.27 3

HG11 HA 0.09 4

HG12 HA 0.09 5

HG13 HA 0.09 6

CG2 CT3 -0.27 7

HG21 HA 0.09 8

HG22 HA 0.09 9

HG23 HA 0.09 10

[ bonds ]

CG1 CB

CG2 CB

CB HB1

CB HB2

CG1 HG11

CG1 HG12

CG1 HG13

CG2 HG21

CG2 HG22

CG2 HG23

[ impropers ]

[ cmap ]

**Supplementary text3**

The atom type and charge of the amino acid analog molecules from OPLS-AA/L force field are described below in the GROMACS rtp format.

[ moleculetype ]

; Name nrexcl

ALA 3

[ atoms ]

; nr type resnr residue atom cgnr charge mass typeB chargeB massB

; residue 1 ALA rtp ALA q 0.0

1 opls_140 1 ALA HB4 1 0.06 1.008 ; qtot 0.06

2 opls_135 1 ALA CB 1 -0.24 12.011 ; qtot -0.18

3 opls_140 1 ALA HB1 1 0.06 1.008 ; qtot -0.12

4 opls_140 1 ALA HB2 1 0.06 1.008 ; qtot -0.06

5 opls_140 1 ALA HB3 1 0.06 1.008 ; qtot 0

[ bonds ]

; ai aj funct c0 c1 c2 c3

1 2 1

2 3 1

2 4 1

2 5 1

[ angles ]

; ai aj ak funct c0 c1 c2 c3

1 2 3 1

1 2 4 1

1 2 5 1

3 2 4 1

3 2 5 1

4 2 5 1

[ moleculetype ]

; Name nrexcl

ARG 3

[ atoms ]

; nr type resnr residue atom cgnr charge mass typeB chargeB massB

; residue 1 ARG rtp ARG q +1.0

1 opls_140 1 ARG HB3 1 0.06 1.008 ; qtot 0.06

2 opls_136 1 ARG CB 2 -0.18 12.011 ; qtot -0.12

3 opls_140 1 ARG HB1 2 0.06 1.008 ; qtot -0.06

4 opls_140 1 ARG HB2 2 0.06 1.008 ; qtot 0

5 opls_308 1 ARG CG 3 -0.05 12.011 ; qtot -0.05

6 opls_140 1 ARG HG1 3 0.06 1.008 ; qtot 0.01

7 opls_140 1 ARG HG2 3 0.06 1.008 ; qtot 0.07

8 opls_307 1 ARG CD 4 0.19 12.011 ; qtot 0.26

9 opls_140 1 ARG HD1 4 0.06 1.008 ; qtot 0.32

10 opls_140 1 ARG HD2 4 0.06 1.008 ; qtot 0.38

11 opls_303 1 ARG NE 5 -0.7 14.0067 ; qtot -0.32

12 opls_304 1 ARG HE 5 0.44 1.008 ; qtot 0.12

13 opls_302 1 ARG CZ 5 0.64 12.011 ; qtot 0.76

14 opls_300 1 ARG NH1 6 -0.8 14.0067 ; qtot -0.04

15 opls_301 1 ARG HH11 6 0.46 1.008 ; qtot 0.42

16 opls_301 1 ARG HH12 6 0.46 1.008 ; qtot 0.88

17 opls_300 1 ARG NH2 7 -0.8 14.0067 ; qtot 0.08

18 opls_301 1 ARG HH21 7 0.46 1.008 ; qtot 0.54

19 opls_301 1 ARG HH22 7 0.46 1.008 ; qtot 1

[ bonds ]

; ai aj funct c0 c1 c2 c3

1 2 1

2 3 1

2 4 1

2 5 1

5 6 1

5 7 1

5 8 1

8 9 1

8 10 1

8 11 1

11 12 1

11 13 1

13 14 1

13 17 1

14 15 1

14 16 1

17 18 1

17 19 1

[ pairs ]

; ai aj funct c0 c1 c2 c3

1 6 1

1 7 1

1 8 1

2 9 1

2 10 1

2 11 1

3 6 1

3 7 1

3 8 1

4 6 1

4 7 1

4 8 1

5 12 1

5 13 1

6 9 1

6 10 1

6 11 1

7 9 1

7 10 1

7 11 1

8 14 1

8 17 1

9 12 1

9 13 1

10 12 1

10 13 1

11 15 1

11 16 1

11 18 1

11 19 1

12 14 1

12 17 1

14 18 1

14 19 1

15 17 1

16 17 1

[ angles ]

; ai aj ak funct c0 c1 c2 c3

1 2 3 1

1 2 4 1

1 2 5 1

3 2 4 1

3 2 5 1

4 2 5 1

2 5 6 1

2 5 7 1

2 5 8 1

6 5 7 1

6 5 8 1

7 5 8 1

5 8 9 1

5 8 10 1

5 8 11 1

9 8 10 1

9 8 11 1

10 8 11 1

8 11 12 1

8 11 13 1

12 11 13 1

11 13 14 1

11 13 17 1

14 13 17 1

13 14 15 1

13 14 16 1

15 14 16 1

13 17 18 1

13 17 19 1

18 17 19 1

[ dihedrals ]

; ai aj ak al funct c0 c1 c2 c3 c4 c5

1 2 5 6 3

1 2 5 7 3

1 2 5 8 3

3 2 5 6 3

3 2 5 7 3

3 2 5 8 3

4 2 5 6 3

4 2 5 7 3

4 2 5 8 3

2 5 8 9 3

2 5 8 10 3

2 5 8 11 3

6 5 8 9 3

6 5 8 10 3

6 5 8 11 3

7 5 8 9 3

7 5 8 10 3

7 5 8 11 3

5 8 11 12 3

5 8 11 13 3

9 8 11 12 3

9 8 11 13 3

10 8 11 12 3

10 8 11 13 3

8 11 13 14 3

8 11 13 17 3

12 11 13 14 3

12 11 13 17 3

11 13 14 15 3

11 13 14 16 3

17 13 14 15 3

17 13 14 16 3

11 13 17 18 3

11 13 17 19 3

14 13 17 18 3

14 13 17 19 3

[ dihedrals ]

; ai aj ak al funct c0 c1 c2 c3

8 13 11 12 1 improper_Z_N_X_Y

11 14 13 17 1 improper_O_C_X_Y

13 15 14 16 1 improper_Z_N_X_Y

13 18 17 19 1 improper_Z_N_X_Y

[ moleculetype ]

; Name nrexcl

ASN 3

[ atoms ]

; nr type resnr residue atom cgnr charge mass typeB chargeB massB

; residue 1 ASN rtp ASN q 0.0

1 opls_140 1 ASN HB3 1 0.06 1.008 ; qtot 0.06

2 opls_136 1 ASN CB 2 -0.18 12.011 ; qtot -0.12

3 opls_140 1 ASN HB1 2 0.06 1.008 ; qtot -0.06

4 opls_140 1 ASN HB2 2 0.06 1.008 ; qtot 0

5 opls_235 1 ASN CG 3 0.5 12.011 ; qtot 0.5

6 opls_236 1 ASN OD1 3 -0.5 15.9994 ; qtot 0

7 opls_237 1 ASN ND2 4 -0.76 14.0067 ; qtot -0.76

8 opls_240 1 ASN HD21 4 0.38 1.008 ; qtot -0.38

9 opls_240 1 ASN HD22 4 0.38 1.008 ; qtot 0

[ bonds ]

; ai aj funct c0 c1 c2 c3

1 2 1

2 3 1

2 4 1

2 5 1

5 6 1

5 7 1

7 8 1

7 9 1

[ pairs ]

; ai aj funct c0 c1 c2 c3

1 6 1

1 7 1

2 8 1

2 9 1

3 6 1

3 7 1

4 6 1

4 7 1

6 8 1

6 9 1

[ angles ]

; ai aj ak funct c0 c1 c2 c3

1 2 3 1

1 2 4 1

1 2 5 1

3 2 4 1

3 2 5 1

4 2 5 1

2 5 6 1

2 5 7 1

6 5 7 1

5 7 8 1

5 7 9 1

8 7 9 1

[ dihedrals ]

; ai aj ak al funct c0 c1 c2 c3 c4 c5

1 2 5 6 3

1 2 5 7 3

3 2 5 6 3

3 2 5 7 3

4 2 5 6 3

4 2 5 7 3

2 5 7 8 3

2 5 7 9 3

6 5 7 8 3

6 5 7 9 3

[ dihedrals ]

; ai aj ak al funct c0 c1 c2 c3

2 7 5 6 1 improper_O_C_X_Y

5 8 7 9 1 improper_Z_N_X_Y

[ moleculetype ]

; Name nrexcl

ASP 3

[ atoms ]

; nr type resnr residue atom cgnr charge mass typeB chargeB massB

; residue 1 ASP rtp ASP q -1.0

1 opls_140 1 ASP HB3 1 0.06 1.008 ; qtot 0.06

2 opls_274 1 ASP CB 2 -0.28 12.011 ; qtot -0.22

3 opls_140 1 ASP HB1 2 0.06 1.008 ; qtot -0.16

4 opls_140 1 ASP HB2 2 0.06 1.008 ; qtot -0.1

5 opls_271 1 ASP CG 3 0.7 12.011 ; qtot 0.6

6 opls_272 1 ASP OD1 3 -0.8 15.9994 ; qtot -0.2

7 opls_272 1 ASP OD2 3 -0.8 15.9994 ; qtot -1

[ bonds ]

; ai aj funct c0 c1 c2 c3

1 2 1

2 3 1

2 4 1

2 5 1

5 6 1

5 7 1

[ pairs ]

; ai aj funct c0 c1 c2 c3

1 6 1

1 7 1

3 6 1

3 7 1

4 6 1

4 7 1

[ angles ]

; ai aj ak funct c0 c1 c2 c3

1 2 3 1

1 2 4 1

1 2 5 1

3 2 4 1

3 2 5 1

4 2 5 1

2 5 6 1

2 5 7 1

6 5 7 1

[ dihedrals ]

; ai aj ak al funct c0 c1 c2 c3 c4 c5

1 2 5 6 3

1 2 5 7 3

3 2 5 6 3

3 2 5 7 3

4 2 5 6 3

4 2 5 7 3

[ dihedrals ]

; ai aj ak al funct c0 c1 c2 c3

2 6 5 7 1 improper_O_C_X_Y

[ moleculetype ]

; Name nrexcl

CYS 3

[ atoms ]

; nr type resnr residue atom cgnr charge mass typeB chargeB massB

; residue 1 CYS rtp CYSH q 0.0

1 opls_140 1 CYS HB3 1 0.06 1.008 ; qtot 0.06

2 opls_206 1 CYS CB 2 0 12.011 ; qtot 0.06

3 opls_140 1 CYS HB1 2 0.06 1.008 ; qtot 0.12

4 opls_140 1 CYS HB2 2 0.06 1.008 ; qtot 0.18

5 opls_200 1 CYS SG 3 -0.335 32.06 ; qtot -0.155

6 opls_204 1 CYS HG 3 0.155 1.008 ; qtot 0

[ bonds ]

; ai aj funct c0 c1 c2 c3

1 2 1

2 3 1

2 4 1

2 5 1

5 6 1

[ pairs ]

; ai aj funct c0 c1 c2 c3

1 6 1

3 6 1

4 6 1

[ angles ]

; ai aj ak funct c0 c1 c2 c3

1 2 3 1

1 2 4 1

1 2 5 1

3 2 4 1

3 2 5 1

4 2 5 1

2 5 6 1

[ dihedrals ]

; ai aj ak al funct c0 c1 c2 c3 c4 c5

1 2 5 6 3

3 2 5 6 3

4 2 5 6 3

[ moleculetype ]

; Name nrexcl

GLN 3

[ atoms ]

; nr type resnr residue atom cgnr charge mass typeB chargeB massB

; residue 1 GLN rtp GLN q 0.0

1 opls_140 1 GLN HB3 1 0.06 1.008 ; qtot 0.06

2 opls_136 1 GLN CB 2 -0.18 12.011 ; qtot -0.12

3 opls_140 1 GLN HB1 2 0.06 1.008 ; qtot -0.06

4 opls_140 1 GLN HB2 2 0.06 1.008 ; qtot 0

5 opls_136 1 GLN CG 3 -0.12 12.011 ; qtot -0.12

6 opls_140 1 GLN HG1 3 0.06 1.008 ; qtot -0.06

7 opls_140 1 GLN HG2 3 0.06 1.008 ; qtot 0

8 opls_235 1 GLN CD 4 0.5 12.011 ; qtot 0.5

9 opls_236 1 GLN OE1 4 -0.5 15.9994 ; qtot 0

10 opls_237 1 GLN NE2 5 -0.76 14.0067 ; qtot -0.76

11 opls_240 1 GLN HE21 5 0.38 1.008 ; qtot -0.38

12 opls_240 1 GLN HE22 5 0.38 1.008 ; qtot 0

[ bonds ]

; ai aj funct c0 c1 c2 c3

1 2 1

2 3 1

2 4 1

2 5 1

5 6 1

5 7 1

5 8 1

8 9 1

8 10 1

10 11 1

10 12 1

[ pairs ]

; ai aj funct c0 c1 c2 c3

1 6 1

1 7 1

1 8 1

2 9 1

2 10 1

3 6 1

3 7 1

3 8 1

4 6 1

4 7 1

4 8 1

5 11 1

5 12 1

6 9 1

6 10 1

7 9 1

7 10 1

9 11 1

9 12 1

[ angles ]

; ai aj ak funct c0 c1 c2 c3

1 2 3 1

1 2 4 1

1 2 5 1

3 2 4 1

3 2 5 1

4 2 5 1

2 5 6 1

2 5 7 1

2 5 8 1

6 5 7 1

6 5 8 1

7 5 8 1

5 8 9 1

5 8 10 1

9 8 10 1

8 10 11 1

8 10 12 1

11 10 12 1

[ dihedrals ]

; ai aj ak al funct c0 c1 c2 c3 c4 c5

1 2 5 6 3

1 2 5 7 3

1 2 5 8 3

3 2 5 6 3

3 2 5 7 3

3 2 5 8 3

4 2 5 6 3

4 2 5 7 3

4 2 5 8 3

2 5 8 9 3

2 5 8 10 3

6 5 8 9 3

6 5 8 10 3

7 5 8 9 3

7 5 8 10 3

5 8 10 11 3

5 8 10 12 3

9 8 10 11 3

9 8 10 12 3

[ dihedrals ]

; ai aj ak al funct c0 c1 c2 c3

5 10 8 9 1 improper_O_C_X_Y

8 11 10 12 1 improper_Z_N_X_Y

[ moleculetype ]

; Name nrexcl

GLU 3

[ atoms ]

; nr type resnr residue atom cgnr charge mass typeB chargeB massB

; residue 1 GLU rtp GLU q -1.0

1 opls_140 1 GLU HB3 1 0.06 1.008 ; qtot 0.06

2 opls_136 1 GLU CB 2 -0.18 12.011 ; qtot -0.12

3 opls_140 1 GLU HB1 2 0.06 1.008 ; qtot -0.06

4 opls_140 1 GLU HB2 2 0.06 1.008 ; qtot 0

5 opls_274 1 GLU CG 3 -0.22 12.011 ; qtot -0.22

6 opls_140 1 GLU HG1 3 0.06 1.008 ; qtot -0.16

7 opls_140 1 GLU HG2 3 0.06 1.008 ; qtot -0.1

8 opls_271 1 GLU CD 4 0.7 12.011 ; qtot 0.6

9 opls_272 1 GLU OE1 4 -0.8 15.9994 ; qtot -0.2

10 opls_272 1 GLU OE2 4 -0.8 15.9994 ; qtot -1

[ bonds ]

; ai aj funct c0 c1 c2 c3

1 2 1

2 3 1

2 4 1

2 5 1

5 6 1

5 7 1

5 8 1

8 9 1

8 10 1

[ pairs ]

; ai aj funct c0 c1 c2 c3

1 6 1

1 7 1

1 8 1

2 9 1

2 10 1

3 6 1

3 7 1

3 8 1

4 6 1

4 7 1

4 8 1

6 9 1

6 10 1

7 9 1

7 10 1

[ angles ]

; ai aj ak funct c0 c1 c2 c3

1 2 3 1

1 2 4 1

1 2 5 1

3 2 4 1

3 2 5 1

4 2 5 1

2 5 6 1

2 5 7 1

2 5 8 1

6 5 7 1

6 5 8 1

7 5 8 1

5 8 9 1

5 8 10 1

9 8 10 1

[ dihedrals ]

; ai aj ak al funct c0 c1 c2 c3 c4 c5

1 2 5 6 3

1 2 5 7 3

1 2 5 8 3

3 2 5 6 3

3 2 5 7 3

3 2 5 8 3

4 2 5 6 3

4 2 5 7 3

4 2 5 8 3

2 5 8 9 3

2 5 8 10 3

6 5 8 9 3

6 5 8 10 3

7 5 8 9 3

7 5 8 10 3

[ dihedrals ]

; ai aj ak al funct c0 c1 c2 c3

5 9 8 10 1 improper_O_C_X_Y

[ moleculetype ]

; Name nrexcl

HISE 3

[ atoms ]

; nr type resnr residue atom cgnr charge mass typeB chargeB massB

; residue 1 HISE rtp HISE q 0.0

1 opls_140 1 HISE HB3 1 0.06 1.008 ; qtot 0.06

2 opls_505 1 HISE CB 2 -0.065 12.011 ; qtot -0.005

3 opls_140 1 HISE HB1 2 0.06 1.008 ; qtot 0.055

4 opls_140 1 HISE HB2 2 0.06 1.008 ; qtot 0.115

5 opls_507 1 HISE CG 3 -0.015 12.011 ; qtot 0.1

6 opls_511 1 HISE ND1 3 -0.49 14.0067 ; qtot -0.39

7 opls_508 1 HISE CD2 4 0.015 12.011 ; qtot -0.375

8 opls_146 1 HISE HD2 4 0.115 1.008 ; qtot -0.26

9 opls_506 1 HISE CE1 5 0.295 12.011 ; qtot 0.035

10 opls_146 1 HISE HE1 5 0.115 1.008 ; qtot 0.15

11 opls_503 1 HISE NE2 6 -0.57 14.0067 ; qtot -0.42

12 opls_504 1 HISE HE2 6 0.42 1.008 ; qtot 0

[ bonds ]

; ai aj funct c0 c1 c2 c3

1 2 1

2 3 1

2 4 1

2 5 1

5 6 1

5 7 1

6 9 1

7 8 1

7 11 1

9 10 1

9 11 1

11 12 1

[ pairs ]

; ai aj funct c0 c1 c2 c3

1 6 1

1 7 1

2 8 1

2 9 1

2 11 1

3 6 1

3 7 1

4 6 1

4 7 1

5 10 1

5 12 1

6 8 1

6 12 1

7 10 1

8 9 1

8 12 1

10 12 1

[ angles ]

; ai aj ak funct c0 c1 c2 c3

1 2 3 1

1 2 4 1

1 2 5 1

3 2 4 1

3 2 5 1

4 2 5 1

2 5 6 1

2 5 7 1

6 5 7 1

5 6 9 1

5 7 8 1

5 7 11 1

8 7 11 1

6 9 10 1

6 9 11 1

10 9 11 1

7 11 9 1

7 11 12 1

9 11 12 1

[ dihedrals ]

; ai aj ak al funct c0 c1 c2 c3 c4 c5

1 2 5 6 3

1 2 5 7 3

3 2 5 6 3

3 2 5 7 3

4 2 5 6 3

4 2 5 7 3

2 5 6 9 3

7 5 6 9 3

2 5 7 8 3

2 5 7 11 3

6 5 7 8 3

6 5 7 11 3

5 6 9 10 3

5 6 9 11 3

5 7 11 9 3

5 7 11 12 3

8 7 11 9 3

8 7 11 12 3

6 9 11 7 3

6 9 11 12 3

10 9 11 7 3

10 9 11 12 3

[ dihedrals ]

; ai aj ak al funct c0 c1 c2 c3

2 5 7 6 1 improper_Z_CA_X_Y

5 11 7 8 1 improper_Z_CA_X_Y

6 11 9 10 1 improper_Z_CA_X_Y

9 7 11 12 1 improper_Z_N_X_Y

[ moleculetype ]

; Name nrexcl

ILE 3

[ atoms ]

; nr type resnr residue atom cgnr charge mass typeB chargeB massB

; residue 1 ILE rtp ILE q 0.0

1 opls_140 1 ILE HB1 1 0.06 1.008 ; qtot 0.06

2 opls_137 1 ILE CB 2 -0.12 12.011 ; qtot -0.06

3 opls_140 1 ILE HB2 2 0.06 1.008 ; qtot 0

4 opls_136 1 ILE CG1 3 -0.12 12.011 ; qtot -0.12

5 opls_140 1 ILE HG11 3 0.06 1.008 ; qtot -0.06

6 opls_140 1 ILE HG12 3 0.06 1.008 ; qtot 0

7 opls_135 1 ILE CG2 4 -0.18 12.011 ; qtot -0.18

8 opls_140 1 ILE HG21 4 0.06 1.008 ; qtot -0.12

9 opls_140 1 ILE HG22 4 0.06 1.008 ; qtot -0.06

10 opls_140 1 ILE HG23 4 0.06 1.008 ; qtot 0

11 opls_135 1 ILE CD 5 -0.18 12.011 ; qtot -0.18

12 opls_140 1 ILE HD1 5 0.06 1.008 ; qtot -0.12

13 opls_140 1 ILE HD2 5 0.06 1.008 ; qtot -0.06

14 opls_140 1 ILE HD3 5 0.06 1.008 ; qtot 0

[ bonds ]

; ai aj funct c0 c1 c2 c3

1 2 1

2 3 1

2 4 1

2 7 1

4 5 1

4 6 1

4 11 1

7 8 1

7 9 1

7 10 1

11 12 1

11 13 1

11 14 1

[ pairs ]

; ai aj funct c0 c1 c2 c3

1 5 1

1 6 1

1 8 1

1 9 1

1 10 1

1 11 1

2 12 1

2 13 1

2 14 1

3 5 1

3 6 1

3 8 1

3 9 1

3 10 1

3 11 1

4 8 1

4 9 1

4 10 1

5 7 1

5 12 1

5 13 1

5 14 1

6 7 1

6 12 1

6 13 1

6 14 1

7 11 1

[ angles ]

; ai aj ak funct c0 c1 c2 c3

1 2 3 1

1 2 4 1

1 2 7 1

3 2 4 1

3 2 7 1

4 2 7 1

2 4 5 1

2 4 6 1

2 4 11 1

5 4 6 1

5 4 11 1

6 4 11 1

2 7 8 1

2 7 9 1

2 7 10 1

8 7 9 1

8 7 10 1

9 7 10 1

4 11 12 1

4 11 13 1

4 11 14 1

12 11 13 1

12 11 14 1

13 11 14 1

[ dihedrals ]

; ai aj ak al funct c0 c1 c2 c3 c4 c5

1 2 4 5 3

1 2 4 6 3

1 2 4 11 3

3 2 4 5 3

3 2 4 6 3

3 2 4 11 3

7 2 4 5 3

7 2 4 6 3

7 2 4 11 3

1 2 7 8 3

1 2 7 9 3

1 2 7 10 3

3 2 7 8 3

3 2 7 9 3

3 2 7 10 3

4 2 7 8 3

4 2 7 9 3

4 2 7 10 3

2 4 11 12 3

2 4 11 13 3

2 4 11 14 3

5 4 11 12 3

5 4 11 13 3

5 4 11 14 3

6 4 11 12 3

6 4 11 13 3

6 4 11 14 3

[ moleculetype ]

; Name nrexcl

LEU 3

[ atoms ]

; nr type resnr residue atom cgnr charge mass typeB chargeB massB

; residue 1 LEU rtp LEU q 0.0

1 opls_140 1 LEU HB3 1 0.06 1.008 ; qtot 0.06

2 opls_136 1 LEU CB 2 -0.18 12.011 ; qtot -0.12

3 opls_140 1 LEU HB1 2 0.06 1.008 ; qtot -0.06

4 opls_140 1 LEU HB2 2 0.06 1.008 ; qtot 0

5 opls_137 1 LEU CG 3 -0.06 12.011 ; qtot -0.06

6 opls_140 1 LEU HG 3 0.06 1.008 ; qtot 0

7 opls_135 1 LEU CD1 4 -0.18 12.011 ; qtot -0.18

8 opls_140 1 LEU HD11 4 0.06 1.008 ; qtot -0.12

9 opls_140 1 LEU HD12 4 0.06 1.008 ; qtot -0.06

10 opls_140 1 LEU HD13 4 0.06 1.008 ; qtot 0

11 opls_135 1 LEU CD2 5 -0.18 12.011 ; qtot -0.18

12 opls_140 1 LEU HD21 5 0.06 1.008 ; qtot -0.12

13 opls_140 1 LEU HD22 5 0.06 1.008 ; qtot -0.06

14 opls_140 1 LEU HD23 5 0.06 1.008 ; qtot 0

[ bonds ]

; ai aj funct c0 c1 c2 c3

2 3 1

2 4 1

2 5 1

5 6 1

5 7 1

5 11 1

7 8 1

7 9 1

7 10 1

11 12 1

11 13 1

11 14 1

[ pairs ]

; ai aj funct c0 c1 c2 c3

2 8 1

2 9 1

2 10 1

2 12 1

2 13 1

2 14 1

3 6 1

3 7 1

3 11 1

4 6 1

4 7 1

4 11 1

6 8 1

6 9 1

6 10 1

6 12 1

6 13 1

6 14 1

7 12 1

7 13 1

7 14 1

8 11 1

9 11 1

10 11 1

[ angles ]

; ai aj ak funct c0 c1 c2 c3

3 2 4 1

3 2 5 1

4 2 5 1

2 5 6 1

2 5 7 1

2 5 11 1

6 5 7 1

6 5 11 1

7 5 11 1

5 7 8 1

5 7 9 1

5 7 10 1

8 7 9 1

8 7 10 1

9 7 10 1

5 11 12 1

5 11 13 1

5 11 14 1

12 11 13 1

12 11 14 1

13 11 14 1

[ dihedrals ]

; ai aj ak al funct c0 c1 c2 c3 c4 c5

3 2 5 6 3

3 2 5 7 3

3 2 5 11 3

4 2 5 6 3

4 2 5 7 3

4 2 5 11 3

2 5 7 8 3

2 5 7 9 3

2 5 7 10 3

6 5 7 8 3

6 5 7 9 3

6 5 7 10 3

11 5 7 8 3

11 5 7 9 3

11 5 7 10 3

2 5 11 12 3

2 5 11 13 3

2 5 11 14 3

6 5 11 12 3

6 5 11 13 3

6 5 11 14 3

7 5 11 12 3

7 5 11 13 3

7 5 11 14 3

[ moleculetype ]

; Name nrexcl

LYS 3

[ atoms ]

; nr type resnr residue atom cgnr charge mass typeB chargeB massB

; residue 1 LYS rtp LYS q +1.0

1 opls_140 1 LYS HB3 1 0.06 1.008 ; qtot 0.06

2 opls_136 1 LYS CB 2 -0.18 12.011 ; qtot -0.12

3 opls_140 1 LYS HB1 2 0.06 1.008 ; qtot -0.06

4 opls_140 1 LYS HB2 2 0.06 1.008 ; qtot 0

5 opls_136 1 LYS CG 3 -0.12 12.011 ; qtot -0.12

6 opls_140 1 LYS HG1 3 0.06 1.008 ; qtot -0.06

7 opls_140 1 LYS HG2 3 0.06 1.008 ; qtot 0

8 opls_136 1 LYS CD 4 -0.12 12.011 ; qtot -0.12

9 opls_140 1 LYS HD1 4 0.06 1.008 ; qtot -0.06

10 opls_140 1 LYS HD2 4 0.06 1.008 ; qtot 0

11 opls_292 1 LYS CE 5 0.19 12.011 ; qtot 0.19

12 opls_140 1 LYS HE1 5 0.06 1.008 ; qtot 0.25

13 opls_140 1 LYS HE2 5 0.06 1.008 ; qtot 0.31

14 opls_287 1 LYS NZ 6 -0.3 14.0067 ; qtot 0.01

15 opls_290 1 LYS HZ1 6 0.33 1.008 ; qtot 0.34

16 opls_290 1 LYS HZ2 6 0.33 1.008 ; qtot 0.67

17 opls_290 1 LYS HZ3 6 0.33 1.008 ; qtot 1

[ bonds ]

; ai aj funct c0 c1 c2 c3

1 2 1

2 3 1

2 4 1

2 5 1

5 6 1

5 7 1

5 8 1

8 9 1

8 10 1

8 11 1

11 12 1

11 13 1

11 14 1

14 15 1

14 16 1

14 17 1

[ pairs ]

; ai aj funct c0 c1 c2 c3

1 6 1

1 7 1

1 8 1

2 9 1

2 10 1

2 11 1

3 6 1

3 7 1

3 8 1

4 6 1

4 7 1

4 8 1

5 12 1

5 13 1

5 14 1

6 9 1

6 10 1

6 11 1

7 9 1

7 10 1

7 11 1

8 15 1

8 16 1

8 17 1

9 12 1

9 13 1

9 14 1

10 12 1

10 13 1

10 14 1

12 15 1

12 16 1

12 17 1

13 15 1

13 16 1

13 17 1

[ angles ]

; ai aj ak funct c0 c1 c2 c3

1 2 3 1

1 2 4 1

1 2 5 1

3 2 4 1

3 2 5 1

4 2 5 1

2 5 6 1

2 5 7 1

2 5 8 1

6 5 7 1

6 5 8 1

7 5 8 1

5 8 9 1

5 8 10 1

5 8 11 1

9 8 10 1

9 8 11 1

10 8 11 1

8 11 12 1

8 11 13 1

8 11 14 1

12 11 13 1

12 11 14 1

13 11 14 1

11 14 15 1

11 14 16 1

11 14 17 1

15 14 16 1

15 14 17 1

16 14 17 1

[ dihedrals ]

; ai aj ak al funct c0 c1 c2 c3 c4 c5

1 2 5 6 3

1 2 5 7 3

1 2 5 8 3

3 2 5 6 3

3 2 5 7 3

3 2 5 8 3

4 2 5 6 3

4 2 5 7 3

4 2 5 8 3

2 5 8 9 3

2 5 8 10 3

2 5 8 11 3

6 5 8 9 3

6 5 8 10 3

6 5 8 11 3

7 5 8 9 3

7 5 8 10 3

7 5 8 11 3

5 8 11 12 3

5 8 11 13 3

5 8 11 14 3

9 8 11 12 3

9 8 11 13 3

9 8 11 14 3

10 8 11 12 3

10 8 11 13 3

10 8 11 14 3

8 11 14 15 3 dih_LYS_chi5_C_C_N_H

8 11 14 16 3 dih_LYS_chi5_C_C_N_H

8 11 14 17 3 dih_LYS_chi5_C_C_N_H

12 11 14 15 3

12 11 14 16 3

12 11 14 17 3

13 11 14 15 3

13 11 14 16 3

13 11 14 17 3

[ moleculetype ]

; Name nrexcl

MET 3

[ atoms ]

; nr type resnr residue atom cgnr charge mass typeB chargeB massB

; residue 1 MET rtp MET q +0.1

1 opls_140 1 MET HB3 1 0.06 1.008 ; qtot 0.06

2 opls_136 1 MET CB 2 -0.12 12.011 ; qtot -0.06

3 opls_140 1 MET HB1 2 0.06 1.008 ; qtot 0

4 opls_140 1 MET HB2 2 0.06 1.008 ; qtot 0.06

5 opls_210 1 MET CG 3 0.048 12.011 ; qtot 0.108

6 opls_140 1 MET HG1 3 0.06 1.008 ; qtot 0.168

7 opls_140 1 MET HG2 3 0.06 1.008 ; qtot 0.228

8 opls_202 1 MET SD 4 -0.335 32.06 ; qtot -0.107

9 opls_209 1 MET CE 5 -0.013 12.011 ; qtot -0.12

10 opls_140 1 MET HE1 5 0.06 1.008 ; qtot -0.06

11 opls_140 1 MET HE2 5 0.06 1.008 ; qtot 0

12 opls_140 1 MET HE3 5 0.06 1.008 ; qtot 0.06

[ bonds ]

; ai aj funct c0 c1 c2 c3

1 2 1

2 3 1

2 4 1

2 5 1

5 6 1

5 7 1

5 8 1

8 9 1

9 10 1

9 11 1

9 12 1

[ pairs ]

; ai aj funct c0 c1 c2 c3

1 6 1

1 7 1

1 8 1

2 9 1

3 6 1

3 7 1

3 8 1

4 6 1

4 7 1

4 8 1

5 10 1

5 11 1

5 12 1

6 9 1

7 9 1

[ angles ]

; ai aj ak funct c0 c1 c2 c3

1 2 3 1

1 2 4 1

1 2 5 1

3 2 4 1

3 2 5 1

4 2 5 1

2 5 6 1

2 5 7 1

2 5 8 1

6 5 7 1

6 5 8 1

7 5 8 1

5 8 9 1

8 9 10 1

8 9 11 1

8 9 12 1

10 9 11 1

10 9 12 1

11 9 12 1

[ dihedrals ]

; ai aj ak al funct c0 c1 c2 c3 c4 c5

1 2 5 6 3

1 2 5 7 3

1 2 5 8 3

3 2 5 6 3

3 2 5 7 3

3 2 5 8 3

4 2 5 6 3

4 2 5 7 3

4 2 5 8 3

2 5 8 9 3

6 5 8 9 3

7 5 8 9 3

5 8 9 10 3

5 8 9 11 3

5 8 9 12 3

[ moleculetype ]

; Name nrexcl

PHE 3

[ atoms ]

; nr type resnr residue atom cgnr charge mass typeB chargeB massB

; residue 1 PHE rtp PHE q 0.0

1 opls_140 1 PHE HB3 1 0.06 1.008 ; qtot 0.06

2 opls_149 1 PHE CB 2 -0.065 12.011 ; qtot -0.005

3 opls_140 1 PHE HB1 2 0.06 1.008 ; qtot 0.055

4 opls_140 1 PHE HB2 2 0.06 1.008 ; qtot 0.115

5 opls_145 1 PHE CG 2 -0.115 12.011 ; qtot 0

6 opls_145 1 PHE CD1 3 -0.115 12.011 ; qtot -0.115

7 opls_146 1 PHE HD1 3 0.115 1.008 ; qtot 0

8 opls_145 1 PHE CD2 4 -0.115 12.011 ; qtot -0.115

9 opls_146 1 PHE HD2 4 0.115 1.008 ; qtot 0

10 opls_145 1 PHE CE1 5 -0.115 12.011 ; qtot -0.115

11 opls_146 1 PHE HE1 5 0.115 1.008 ; qtot 0

12 opls_145 1 PHE CE2 6 -0.115 12.011 ; qtot -0.115

13 opls_146 1 PHE HE2 6 0.115 1.008 ; qtot 0

14 opls_145 1 PHE CZ 7 -0.115 12.011 ; qtot -0.115

15 opls_146 1 PHE HZ 7 0.115 1.008 ; qtot 0

[ bonds ]

; ai aj funct c0 c1 c2 c3

1 2 1

2 3 1

2 4 1

2 5 1

5 6 1

5 8 1

6 7 1

6 10 1

8 9 1

8 12 1

10 11 1

10 14 1

12 13 1

12 14 1

14 15 1

[ pairs ]

; ai aj funct c0 c1 c2 c3

1 6 1

1 8 1

2 7 1

2 9 1

2 10 1

2 12 1

3 6 1

3 8 1

4 6 1

4 8 1

5 11 1

5 13 1

5 14 1

6 9 1

6 12 1

6 15 1

7 8 1

7 11 1

7 14 1

8 10 1

8 15 1

9 13 1

9 14 1

10 13 1

11 12 1

11 15 1

13 15 1

[ angles ]

; ai aj ak funct c0 c1 c2 c3

1 2 3 1

1 2 4 1

1 2 5 1

3 2 4 1

3 2 5 1

4 2 5 1

2 5 6 1

2 5 8 1

6 5 8 1

5 6 7 1

5 6 10 1

7 6 10 1

5 8 9 1

5 8 12 1

9 8 12 1

6 10 11 1

6 10 14 1

11 10 14 1

8 12 13 1

8 12 14 1

13 12 14 1

10 14 12 1

10 14 15 1

12 14 15 1

[ dihedrals ]

; ai aj ak al funct c0 c1 c2 c3 c4 c5

1 2 5 6 3

1 2 5 8 3

3 2 5 6 3

3 2 5 8 3

4 2 5 6 3

4 2 5 8 3

2 5 6 7 3

2 5 6 10 3

8 5 6 7 3

8 5 6 10 3

2 5 8 9 3

2 5 8 12 3

6 5 8 9 3

6 5 8 12 3

5 6 10 11 3

5 6 10 14 3

7 6 10 11 3

7 6 10 14 3

5 8 12 13 3

5 8 12 14 3

9 8 12 13 3

9 8 12 14 3

6 10 14 12 3

6 10 14 15 3

11 10 14 12 3

11 10 14 15 3

8 12 14 10 3

8 12 14 15 3

13 12 14 10 3

13 12 14 15 3

[ dihedrals ]

; ai aj ak al funct c0 c1 c2 c3

2 5 8 6 1 improper_Z_CA_X_Y

5 10 6 7 1 improper_Z_CA_X_Y

5 12 8 9 1 improper_Z_CA_X_Y

6 14 10 11 1 improper_Z_CA_X_Y

8 14 12 13 1 improper_Z_CA_X_Y

10 12 14 15 1 improper_Z_CA_X_Y

[ moleculetype ]

; Name nrexcl

SER 3

[ atoms ]

; nr type resnr residue atom cgnr charge mass typeB chargeB massB

; residue 1 SER rtp SER q 0.0

1 opls_140 1 SER HB3 1 0.06 1.008 ; qtot 0.06

2 opls_157 1 SER CB 2 0.085 12.011 ; qtot 0.145

3 opls_140 1 SER HB1 2 0.06 1.008 ; qtot 0.205

4 opls_140 1 SER HB2 2 0.06 1.008 ; qtot 0.265

5 opls_154 1 SER OG 3 -0.683 15.9994 ; qtot -0.418

6 opls_155 1 SER HG 3 0.418 1.008 ; qtot 0

[ bonds ]

; ai aj funct c0 c1 c2 c3

1 2 1

2 3 1

2 4 1

2 5 1

5 6 1

[ pairs ]

; ai aj funct c0 c1 c2 c3

1 6 1

3 6 1

4 6 1

[ angles ]

; ai aj ak funct c0 c1 c2 c3

1 2 3 1

1 2 4 1

1 2 5 1

3 2 4 1

3 2 5 1

4 2 5 1

2 5 6 1

[ dihedrals ]

; ai aj ak al funct c0 c1 c2 c3 c4 c5

1 2 5 6 3

3 2 5 6 3

4 2 5 6 3

[ moleculetype ]

; Name nrexcl

THR 3

[ atoms ]

; nr type resnr residue atom cgnr charge mass typeB chargeB massB

; residue 1 THR rtp THR q 0.0

1 opls_140 1 THR HB1 1 0.06 1.008 ; qtot 0.06

2 opls_158 1 THR CB 2 0.145 12.011 ; qtot 0.205

3 opls_140 1 THR HB2 2 0.06 1.008 ; qtot 0.265

4 opls_154 1 THR OG1 2 -0.683 15.9994 ; qtot -0.418

5 opls_155 1 THR HG1 2 0.418 1.008 ; qtot 0

6 opls_135 1 THR CG2 3 -0.18 12.011 ; qtot -0.18

7 opls_140 1 THR HG21 3 0.06 1.008 ; qtot -0.12

8 opls_140 1 THR HG22 3 0.06 1.008 ; qtot -0.06

9 opls_140 1 THR HG23 3 0.06 1.008 ; qtot 0

[ bonds ]

; ai aj funct c0 c1 c2 c3

1 2 1

2 3 1

2 4 1

2 6 1

4 5 1

6 7 1

6 8 1

6 9 1

[ pairs ]

; ai aj funct c0 c1 c2 c3

1 5 1

1 7 1

1 8 1

1 9 1

3 5 1

3 7 1

3 8 1

3 9 1

4 7 1

4 8 1

4 9 1

5 6 1

[ angles ]

; ai aj ak funct c0 c1 c2 c3

1 2 3 1

1 2 4 1

1 2 6 1

3 2 4 1

3 2 6 1

4 2 6 1

2 4 5 1

2 6 7 1

2 6 8 1

2 6 9 1

7 6 8 1

7 6 9 1

8 6 9 1

[ dihedrals ]

; ai aj ak al funct c0 c1 c2 c3 c4 c5

1 2 4 5 3

3 2 4 5 3

6 2 4 5 3

1 2 6 7 3

1 2 6 8 3

1 2 6 9 3

3 2 6 7 3

3 2 6 8 3

3 2 6 9 3

4 2 6 7 3

4 2 6 8 3

4 2 6 9 3

[ moleculetype ]

; Name nrexcl

TRP 3

[ atoms ]

; nr type resnr residue atom cgnr charge mass typeB chargeB massB

; residue 1 TRP rtp TRP q 0.0

1 opls_140 1 TRP HB3 1 0.06 1.008 ; qtot 0.06

2 opls_136 1 TRP CB 2 -0.18 12.011 ; qtot -0.12

3 opls_140 1 TRP HB1 2 0.06 1.008 ; qtot -0.06

4 opls_140 1 TRP HB2 2 0.06 1.008 ; qtot 0

5 opls_500 1 TRP CG 3 0.075 12.011 ; qtot 0.075

6 opls_514 1 TRP CD1 4 -0.115 12.011 ; qtot -0.04

7 opls_146 1 TRP HD1 4 0.115 1.008 ; qtot 0.075

8 opls_501 1 TRP CD2 5 -0.055 12.011 ; qtot 0.02

9 opls_503 1 TRP NE1 6 -0.57 14.0067 ; qtot -0.55

10 opls_504 1 TRP HE1 6 0.42 1.008 ; qtot -0.13

11 opls_502 1 TRP CE2 6 0.13 12.011 ; qtot 0

12 opls_145 1 TRP CE3 7 -0.115 12.011 ; qtot -0.115

13 opls_146 1 TRP HE3 7 0.115 1.008 ; qtot 0

14 opls_145 1 TRP CZ2 8 -0.115 12.011 ; qtot -0.115

15 opls_146 1 TRP HZ2 8 0.115 1.008 ; qtot 0

16 opls_145 1 TRP CZ3 9 -0.115 12.011 ; qtot -0.115

17 opls_146 1 TRP HZ3 9 0.115 1.008 ; qtot 0

18 opls_145 1 TRP CH2 10 -0.115 12.011 ; qtot -0.115

19 opls_146 1 TRP HH2 10 0.115 1.008 ; qtot 0

[ bonds ]

; ai aj funct c0 c1 c2 c3

1 2 1

2 3 1

2 4 1

2 5 1

5 6 1

5 8 1

6 7 1

6 9 1

8 11 1

8 12 1

9 10 1

9 11 1

11 14 1

12 13 1

12 16 1

14 15 1

14 18 1

16 17 1

16 18 1

18 19 1

[ pairs ]

; ai aj funct c0 c1 c2 c3

1 6 1

1 8 1

2 7 1

2 9 1

2 11 1

2 12 1

3 6 1

3 8 1

4 6 1

4 8 1

5 10 1

5 13 1

5 14 1

5 16 1

6 12 1

6 14 1

7 8 1

7 10 1

7 11 1

8 10 1

8 15 1

8 17 1

8 18 1

9 12 1

9 15 1

9 18 1

10 14 1

11 13 1

11 16 1

11 19 1

12 14 1

12 19 1

13 17 1

13 18 1

14 17 1

15 16 1

15 19 1

17 19 1

[ angles ]

; ai aj ak funct c0 c1 c2 c3

1 2 3 1

1 2 4 1

1 2 5 1

3 2 4 1

3 2 5 1

4 2 5 1

2 5 6 1

2 5 8 1

6 5 8 1

5 6 7 1

5 6 9 1

7 6 9 1

5 8 11 1

5 8 12 1

11 8 12 1

6 9 10 1

6 9 11 1

10 9 11 1

8 11 9 1

8 11 14 1

9 11 14 1

8 12 13 1

8 12 16 1

13 12 16 1

11 14 15 1

11 14 18 1

15 14 18 1

12 16 17 1

12 16 18 1

17 16 18 1

14 18 16 1

14 18 19 1

16 18 19 1

[ dihedrals ]

; ai aj ak al funct c0 c1 c2 c3 c4 c5

1 2 5 6 3

1 2 5 8 3

3 2 5 6 3

3 2 5 8 3

4 2 5 6 3

4 2 5 8 3

2 5 6 7 3

2 5 6 9 3

8 5 6 7 3

8 5 6 9 3

2 5 8 11 3

2 5 8 12 3

6 5 8 11 3

6 5 8 12 3

5 6 9 10 3

5 6 9 11 3

7 6 9 10 3

7 6 9 11 3

5 8 11 9 3

5 8 11 14 3

12 8 11 9 3

12 8 11 14 3

5 8 12 13 3

5 8 12 16 3

11 8 12 13 3

11 8 12 16 3

6 9 11 8 3

6 9 11 14 3

10 9 11 8 3

10 9 11 14 3

8 11 14 15 3

8 11 14 18 3

9 11 14 15 3

9 11 14 18 3

8 12 16 17 3

8 12 16 18 3

13 12 16 17 3

13 12 16 18 3

11 14 18 16 3

11 14 18 19 3

15 14 18 16 3

15 14 18 19 3

12 16 18 14 3

12 16 18 19 3

17 16 18 14 3

17 16 18 19 3

[ dihedrals ]

; ai aj ak al funct c0 c1 c2 c3

2 5 8 6 1 improper_Z_CA_X_Y

5 9 6 7 1 improper_Z_CA_X_Y

6 11 9 10 1 improper_Z_N_X_Y

11 18 14 15 1 improper_Z_CA_X_Y

13 12 8 16 1 improper_Z_CA_X_Y

14 16 18 19 1 improper_Z_CA_X_Y

17 16 12 18 1 improper_Z_CA_X_Y

[ moleculetype ]

; Name nrexcl

TYR 3

[ atoms ]

; nr type resnr residue atom cgnr charge mass typeB chargeB massB

; residue 1 TYR rtp TYR q 0.0

1 opls_140 1 TYR HB3 1 0.06 1.008 ; qtot 0.06

2 opls_149 1 TYR CB 2 -0.065 12.011 ; qtot -0.005

3 opls_140 1 TYR HB1 2 0.06 1.008 ; qtot 0.055

4 opls_140 1 TYR HB2 2 0.06 1.008 ; qtot 0.115

5 opls_145 1 TYR CG 2 -0.115 12.011 ; qtot 0

6 opls_145 1 TYR CD1 3 -0.115 12.011 ; qtot -0.115

7 opls_146 1 TYR HD1 3 0.115 1.008 ; qtot 0

8 opls_145 1 TYR CD2 4 -0.115 12.011 ; qtot -0.115

9 opls_146 1 TYR HD2 4 0.115 1.008 ; qtot 0

10 opls_145 1 TYR CE1 5 -0.115 12.011 ; qtot -0.115

11 opls_146 1 TYR HE1 5 0.115 1.008 ; qtot 0

12 opls_145 1 TYR CE2 6 -0.115 12.011 ; qtot -0.115

13 opls_146 1 TYR HE2 6 0.115 1.008 ; qtot 0

14 opls_166 1 TYR CZ 7 0.15 12.011 ; qtot 0.15

15 opls_167 1 TYR OH 7 -0.585 15.9994 ; qtot -0.435

16 opls_168 1 TYR HH 7 0.435 1.008 ; qtot 0

[ bonds ]

; ai aj funct c0 c1 c2 c3

1 2 1

2 3 1

2 4 1

2 5 1

5 6 1

5 8 1

6 7 1

6 10 1

8 9 1

8 12 1

10 11 1

10 14 1

12 13 1

12 14 1

14 15 1

15 16 1

[ pairs ]

; ai aj funct c0 c1 c2 c3

1 6 1

1 8 1

2 7 1

2 9 1

2 10 1

2 12 1

3 6 1

3 8 1

4 6 1

4 8 1

5 11 1

5 13 1

5 14 1

6 9 1

6 12 1

6 15 1

7 8 1

7 11 1

7 14 1

8 10 1

8 15 1

9 13 1

9 14 1

10 13 1

10 16 1

11 12 1

11 15 1

12 16 1

13 15 1

[ angles ]

; ai aj ak funct c0 c1 c2 c3

1 2 3 1

1 2 4 1

1 2 5 1

3 2 4 1

3 2 5 1

4 2 5 1

2 5 6 1

2 5 8 1

6 5 8 1

5 6 7 1

5 6 10 1

7 6 10 1

5 8 9 1

5 8 12 1

9 8 12 1

6 10 11 1

6 10 14 1

11 10 14 1

8 12 13 1

8 12 14 1

13 12 14 1

10 14 12 1

10 14 15 1

12 14 15 1

14 15 16 1

[ dihedrals ]

; ai aj ak al funct c0 c1 c2 c3 c4 c5

1 2 5 6 3

1 2 5 8 3

3 2 5 6 3

3 2 5 8 3

4 2 5 6 3

4 2 5 8 3

2 5 6 7 3

2 5 6 10 3

8 5 6 7 3

8 5 6 10 3

2 5 8 9 3

2 5 8 12 3

6 5 8 9 3

6 5 8 12 3

5 6 10 11 3

5 6 10 14 3

7 6 10 11 3

7 6 10 14 3

5 8 12 13 3

5 8 12 14 3

9 8 12 13 3

9 8 12 14 3

6 10 14 12 3

6 10 14 15 3

11 10 14 12 3

11 10 14 15 3

8 12 14 10 3

8 12 14 15 3

13 12 14 10 3

13 12 14 15 3

10 14 15 16 3

12 14 15 16 3

[ dihedrals ]

; ai aj ak al funct c0 c1 c2 c3

2 5 8 6 1 improper_Z_CA_X_Y

5 10 6 7 1 improper_Z_CA_X_Y

5 12 8 9 1 improper_Z_CA_X_Y

6 14 10 11 1 improper_Z_CA_X_Y

8 14 12 13 1 improper_Z_CA_X_Y

10 12 14 15 1 improper_Z_CA_X_Y

[ moleculetype ]

; Name nrexcl

VAL 3

[ atoms ]

; nr type resnr residue atom cgnr charge mass typeB chargeB massB

; residue 1 VAL rtp VAL q 0.0

1 opls_140 1 VAL HB1 1 0.06 1.008 ; qtot 0.06

2 opls_137 1 VAL CB 2 -0.12 12.011 ; qtot -0.06

3 opls_140 1 VAL HB2 2 0.06 1.008 ; qtot 0

4 opls_135 1 VAL CG1 3 -0.18 12.011 ; qtot -0.18

5 opls_140 1 VAL HG11 3 0.06 1.008 ; qtot -0.12

6 opls_140 1 VAL HG12 3 0.06 1.008 ; qtot -0.06

7 opls_140 1 VAL HG13 3 0.06 1.008 ; qtot 0

8 opls_135 1 VAL CG2 4 -0.18 12.011 ; qtot -0.18

9 opls_140 1 VAL HG21 4 0.06 1.008 ; qtot -0.12

10 opls_140 1 VAL HG22 4 0.06 1.008 ; qtot -0.06

11 opls_140 1 VAL HG23 4 0.06 1.008 ; qtot 0

[ bonds ]

; ai aj funct c0 c1 c2 c3

1 2 1

2 3 1

2 4 1

2 8 1

4 5 1

4 6 1

4 7 1

8 9 1

8 10 1

8 11 1

[ pairs ]

; ai aj funct c0 c1 c2 c3

1 5 1

1 6 1

1 7 1

1 9 1

1 10 1

1 11 1

3 5 1

3 6 1

3 7 1

3 9 1

3 10 1

3 11 1

4 9 1

4 10 1

4 11 1

5 8 1

6 8 1

7 8 1

[ angles ]

; ai aj ak funct c0 c1 c2 c3

1 2 3 1

1 2 4 1

1 2 8 1

3 2 4 1

3 2 8 1

4 2 8 1

2 4 5 1

2 4 6 1

2 4 7 1

5 4 6 1

5 4 7 1

6 4 7 1

2 8 9 1

2 8 10 1

2 8 11 1

9 8 10 1

9 8 11 1

10 8 11 1

[ dihedrals ]

; ai aj ak al funct c0 c1 c2 c3 c4 c5

1 2 4 5 3

1 2 4 6 3

1 2 4 7 3

3 2 4 5 3

3 2 4 6 3

3 2 4 7 3

8 2 4 5 3

8 2 4 6 3

8 2 4 7 3

1 2 8 9 3

1 2 8 10 3

1 2 8 11 3

3 2 8 9 3

3 2 8 10 3

3 2 8 11 3

4 2 8 9 3

4 2 8 10 3

4 2 8 11 3
